# Safer and efficient base editing and prime editing via ribonucleoproteins delivered through optimized lipid-nanoparticle formulations

**DOI:** 10.1038/s41551-024-01296-2

**Published:** 2024-11-28

**Authors:** Rafał Hołubowicz, Samuel W. Du, Jiin Felgner, Roman Smidak, Elliot H. Choi, Grazyna Palczewska, Carolline Rodrigues Menezes, Zhiqian Dong, Fangyuan Gao, Omar Medani, Alexander L. Yan, Maria W. Hołubowicz, Paul Z. Chen, Marco Bassetto, Eleonora Risaliti, David Salom, J. Noah Workman, Philip D. Kiser, Andrzej T. Foik, David C. Lyon, Gregory A. Newby, David R. Liu, Philip L. Felgner, Krzysztof Palczewski

**Affiliations:** 1 Gavin Herbert Eye Institute – Center for Translational Vision Research, Department of Ophthalmology, University of California, Irvine, CA USA; 2 Department of Biochemistry, Molecular Biology and Biotechnology, Faculty of Chemistry, Wroclaw University of Science and Technology, Wroclaw, Poland; 3 Department of Physiology and Biophysics, School of Medicine, University of California, Irvine, CA USA; 4 Adeline Yen Mah Vaccine Center, Department of Physiology and Biophysics, University of California, Irvine, CA USA; 5 Program in Neuroscience, Amherst College, Amherst, MA USA; 6 Merkin Institute of Transformative Technologies in Healthcare, Broad Institute of Harvard and MIT, Cambridge, MA USA; 7 Department of Chemistry and Chemical Biology, Harvard University, Cambridge, MA USA; 8 Howard Hughes Medical Institute, Harvard University, Cambridge, MA USA; 9 David H. Koch Institute for Integrative Cancer Research, Massachusetts Institute of Technology, Cambridge, MA USA; 10 Research Service, Tibor Rubin VA Long Beach Medical Center, Long Beach, CA USA; 11 Department of Genetic Medicine, Johns Hopkins University, Baltimore, MD USA; 12 Department of Clinical Pharmacy Practice, School of Pharmacy and Pharmaceutical Sciences, University of California, Irvine, CA USA; 13 International Centre for Translational Eye Research (ICTER), Institute of Physical Chemistry, Polish Academy of Sciences, Warsaw, Poland; 14 Institute of Physical Chemistry, Polish Academy of Sciences, Warsaw, Poland; 15 Department of Anatomy and Neurobiology, School of Medicine, University of California, Irvine, CA USA; 16 Department of Chemistry, University of California, Irvine, CA USA; 17 Department of Molecular Biology and Biochemistry, University of California, Irvine, CA USA

**Keywords:** CRISPR-Cas9 genome editing, Experimental models of disease

## Abstract

Delivering ribonucleoproteins (RNPs) for in vivo genome editing is safer than using viruses encoding for Cas9 and its respective guide RNA. However, transient RNP activity does not typically lead to optimal editing outcomes. Here we show that the efficiency of delivering RNPs can be enhanced by cell-penetrating peptides (covalently fused to the protein or as excipients) and that lipid nanoparticles (LNPs) encapsulating RNPs can be optimized for enhanced RNP stability, delivery efficiency and editing potency. Specifically, after screening for suitable ionizable cationic lipids and by optimizing the concentration of the synthetic lipid DMG-PEG 2000, we show that the encapsulation, via microfluidic mixing, of adenine base editor and prime editor RNPs within LNPs using the ionizable lipid SM102 can result in in vivo editing-efficiency enhancements larger than 300-fold (with respect to the delivery of the naked RNP) without detectable off-target edits. We believe that chemically defined LNP formulations optimized for RNP-encapsulation stability and delivery efficiency will lead to safer genome editing.

## Abstract (web summary)

The safety and efficacy of ribonucleoproteins for genome editing can be enhanced by formulations of lipid nanoparticles optimized for enhanced stability, delivery efficiency and editing potency of the protein complexes.

## Main

Monogenic diseases arise from genetic mutations that lead to aberrant or absent gene expression, and many lack appropriate therapies. Advances in molecular biology have enabled several treatment approaches to address this unmet medical need and correct the molecular basis of inherited diseases. Gene augmentation therapy, for example, delivers a wild-type (WT) copy of a mutated gene via a viral vector to supplement expression^1^. However, gene augmentation is limited by several major shortcomings, including potential loss of expression over long periods, lack of endogenous gene regulation, the inability to package large transgenes^2^ and low efficacy when treating mutations that act in a dominant manner^3,4^. An alternative approach to gene augmentation is genome editing. By correcting the genomic mutation in situ, a one-time treatment could be curative for the lifetime of the patient^5,6^. Of the gene editing techniques, clustered regularly interspaced short palindromic repeats and CRISPR-associated protein 9 (CRISPR/Cas9) editing has shown great promise and has advanced to clinical trials^7^. While these early trials have focused on treating monogenic diseases, it is possible to envision the application of genome editing for the treatment or prevention of common diseases with multifactorial or polygenic causes, such as malignancies^8,9^, cardiovascular disorders^10^ or neurodegenerative diseases^11^.

The CRISPR/Cas9 system used in gene editing trials consists of a Cas9 nuclease that is targeted to a genomic site by a protospacer-adjacent motif (PAM) and guide RNA that focuses the binding of Cas9 on a 20-bp-long DNA protospacer^12^. While CRISPR/Cas9 is easily programmed by the substitution of the guide RNA, the double-stranded DNA cleavage mediated by the nuclease can lead to a heterogeneous pool of editing outcomes, namely random insertions and deletions (indels) by non-homologous end joining (NHEJ), as well as cytotoxicity, p53 pathway activation, and large chromosomal irregularities and rearrangements^7^. Moreover, the efficiency of precise repair via homology-directed repair with a donor DNA template is low compared with NHEJ, especially in post-mitotic cells^13^, including major cell types of interest such as neurons.

Two alternatives to genome editing with CRISPR/Cas9 nucleases are base and prime editors, which fuse a DNA effector domain to a partially inactivated Cas9 domain, termed a Cas9 nickase^14–16^. These modifications combine the ease of programmability of CRISPR/Cas9 with the precision and direct chemistry of the effector domain of the base or prime editor, while avoiding NHEJ and cytotoxicity caused by double-stranded DNA breaks^17^. Thus, the purity of editing outcomes is much greater for base and prime editors; because the DNA-repair mechanisms that enable base and prime editing are cell-cycle independent, the high editing efficiencies are maintained in post-mitotic cell types when genome editing is performed in vivo^18^. CRISPR/Cas9 strategies, and base and prime editing in particular, are suitable approaches for dominant-negative diseases through correction of the pathogenic allele^19,20^. However, bystander editing by base editors is a concern^21^, as it could lead to unintended changes and hamper the therapeutic efficacy for the patient^21^. As well, the potential off-target effects of prime editors have not yet been carefully explored and documented.

One major hurdle that limits the application of base and prime editors is appropriate and efficient delivery of these editing constructs. The current standard for delivery of gene therapy and gene editing constructs is via viral vectors. However, the net size of the guide RNA constructs along with the base or prime editors exceeds the packaging limits of most commonly used viral vectors, such as lentiviruses (LV) and adeno-associated viruses (AAVs); indeed, base and prime editors delivered by AAV often are split into two viral vectors^22^. In addition, while these viral vectors have been engineered to be less immunogenic than their native counterparts, they still express base and prime editors over a sustained period^23^. Whereas the intended ‘on-target’ site is favoured thermodynamically to be edited, prolonged expression of base and prime editors leads to an increased risk of off-target editing at less-favoured sites on the genome and transcriptome^24^, either in a Cas9-dependent^25^ or in an effector-dependent manner^26,27^. Prolonged exposure to base editors has been also shown to increase bystander editing^28^. Lastly, there is a non-zero risk of viral genome integration, even when non-integrating viral vectors are used^29^, and this risk could even be worsened by the deployment of Cas9 nucleases and nickases^30,31^. Thus, as sustained expression of CRISPR/Cas9 is unnecessary and only risks unintended editing outcomes, CRISPR/Cas9 should be delivered in a transient manner and rapidly degraded thereafter. Multiple ways of achieving this transient action have been proposed, such as by virus-like particle (VLP) delivery^28,32,33^, mRNA lipid-nanoparticle (LNP) delivery^34^ and by direct ribonucleoprotein (RNP) delivery, either as naked RNP or as a lipoplex with cationic lipid reagents such as Lipofectamine 2000^35–38^. Such a variety of approaches has the potential to fine-tune the duration of activity of CRISPR/Cas9 machinery; however, these alternative delivery mechanisms require further refinement.

Conceptually, delivery of preassembled RNPs offers the most rapid onset and the shortest duration of genome editor activity in the cell. Real-world human data for AAV-mediated delivery of retinal pigment epithelium-specific 65 kDa protein (RPE65) by voretigene neparvovec (Luxturna) show that the therapeutic effect may be maintained for 7 years and potentially longer, demonstrating sustained expression of the transgene delivered by AAV^39^. Protein expression after subretinal delivery of mRNA was detected within 4 h and lasted for up to 7 days^40^. In the case of RNP, the purified protein is complexed with synthetic guide RNA before direct delivery into cells, thus bypassing the requirement of transcription (AAV, LV) and translation (AAV, LV, mRNA). VLPs that have RNP encapsulated in a viral shell offer the same advantageous activity kinetics as RNP; however, they are not chemically defined, which may complicate their manufacturing and clinical application. Use of purified RNP thus offers the most transient and chemically defined delivery modality for CRISPR/Cas9.

Previously, we reported the successful correction of the causative mutation in the *rd12* mouse model of Leber congenital amaurosis (LCA) through delivery of an adenine base editor (ABE) via LV^41^, AAV^42^ and engineered virus-like particles (eVLP)^28^. Likewise, we recently delivered prime editor (PE) via eVLP and achieved effective and precise correction of the *rd12* mutation^43^. However, we expect that we can create a more defined, clinically relevant formulation of CRISPR/Cas9-based genome editor through optimization of direct delivery of base- and prime-editor RNPs.

Here, following a screening of cell-penetrating peptides (CPPs) and commercially available ionizable cationic lipids with acid disassociation constants (p*K*_a_) > 6, we show the restoration of visual function in a mouse model of inherited retinal degeneration using purified ABE and PE RNPs encapsulated in lipid nanoparticle LNPs. We hope that our results will open the way to chemically defined and protected delivery technologies for CRISPR/Cas9-mediated genome editing.

## Results

### Expression and characterization of Cre, ABE and PE

**Fig. 1:**
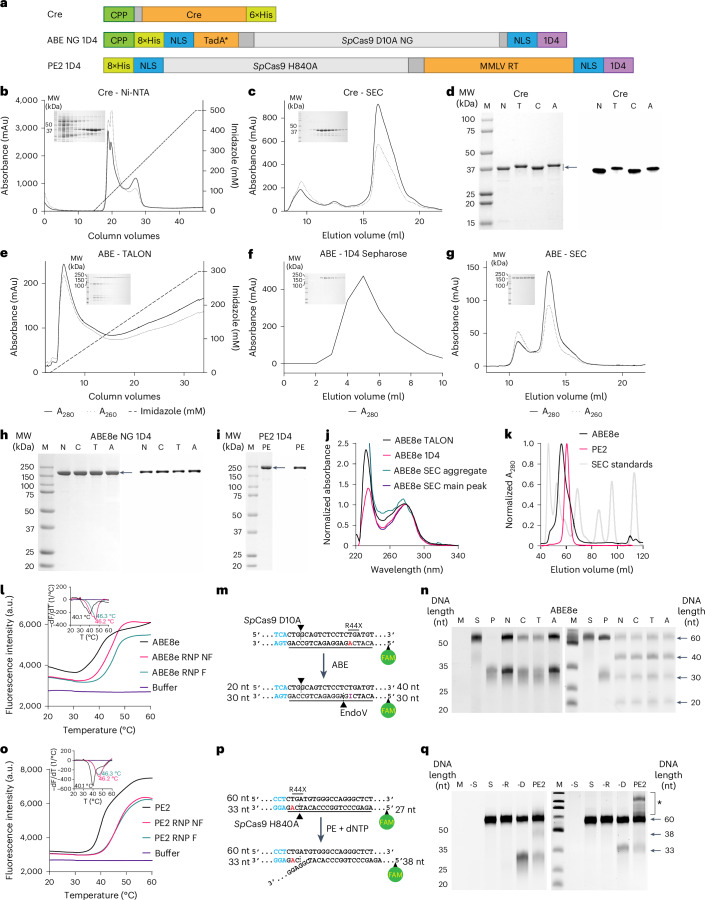
Purification and characterization of genome-editing proteins. **a**, Schematic cartoon of protein constructs utilized in this study. 1D4, 9 amino acid C-terminal 1D4 rhodopsin peptide; CPP, cell-penetrating peptide; MMLV RT, Moloney murine leukaemia virus reverse transcriptase; NLS, nuclear localization signal; TadA* 8e, engineered tRNA deaminase. **b**,**c**, Purification of Cre recombinase by Ni-NTA (**b**) and SEC (**c**). Insets: SDS–PAGE gels of collected fractions after CBB staining. **d**, SDS–PAGE (left) and western blot analysis (right) of purified Cre recombinase. M, molecular weight marker; N, N-Cre-His; T, TAT-Cre-His; C, CPP5-Cre-His; A, ANTP-Cre-His. **e**–**g**, TALON chromatography (**e**), 1D4 chromatography (**f**) and SEC (**g**), representing purification of ABE and PE proteins. Insets: SDS–PAGE gels of collected fractions stained with CBB. **h**,**i**, SDS–PAGE (left) and western blot (right) analyses of purified ABE and PE proteins. **j**, Absorbance spectra of the fractions collected during purification of ABE, showing gradual removal of contaminating nucleic acids by decreasing absorbance at 260 nm relative to 280 nm. **k**, SEC of ABE and PE proteins relative to standards consisting of blue dextran (2,000 kDa, determining void column volume), thyroglobulin (670 kDa), γ-globulin (158 kDa), ovalbumin (44 kDa), myoglobin (17 kDa) and vitamin B12 (1.35 kDa, determining accessible column volume). **l**, Averaged (*n* = 3) DSF profiles of ABE with guide RNA in PBS containing 10% (w/v) sucrose. Inserts: rate of change of fluorescence intensity (−dF/dT) and melting temperatures. NF, non-folded guide RNA; F, folded guide RNA. **m**, Schematic diagram of the ABE activity assay. Blue, SpCas9 PAM; red, target base; arrowhead, nick site; FAM, fluorescein. **n**, Urea–PAGE gels imaged for fluorescein (left) and SYBR Gold (right), demonstrating activity of ABE in vitro. M, DNA standard; S, substrate; N, ABE without fused cell-penetrating peptide; C, CPP5-ABE; T, TAT-ABE; A, ANTP-ABE. **o**, Averaged (*n* = 3) DSF profiles of PE with guide RNA in PBS. Inserts: rate of change of fluorescence intensity (−dF/dT) and melting temperatures. **p**, Schematic diagram of the PE activity assay. Blue, SpCas9 PAM; red, target base; arrowhead, nick site; FAM, fluorescein. **q**, Urea–PAGE gel imaged for fluorescein (left) and SYBR Gold (right), demonstrating activity of PE. ‘−S’, no substrate control; ‘S’, substrate-only control; ‘−R’, no epegRNA control; ‘−D’, no dNTP control; PE2, sample containing PE RNP and dNTP. Asterisk indicates products extended beyond the reverse transcriptase template. Uncropped gels and blots are available in Source data. Source data

The potential for therapeutic benefit from administered proteins hinges upon successful delivery across cellular membranes. A number of agents have been demonstrated to enhance intracellular delivery of genome-editing proteins and RNPs, including CPPs. Accordingly, we genetically fused three different CPPs (TAT, CPP5 and ANTP)^44–47^ to the N terminus of Cre recombinase and ABE8e-SpCas9-NG (hereafter referred to as ABE8e), which recognizes the NG PAM (Fig. 1a). To facilitate purification, we also fused the 1D4 peptide tag^48^ to the C terminus of ABE8e and the prime editor protein, PE2 (Fig. 1a). Although Cre recombinase and Cas9-based genome-editing proteins possess distinct physicochemical properties, such as size and charge, we hypothesized that insights from the study of intracellular delivery of Cre recombinase would inform the design of delivery vehicles for ABE8e and PE2.

We first purified Cre recombinase to homogeneity, with or without the N-terminal CPP fusion, through nickel metal-affinity chromatography (Ni-NTA) chromatography (Fig. 1b) and size exclusion chromatography (SEC, Fig. 1c), and confirmed the purity of the product through sodium dodecyl sulfate–polyacrylamide gel electrophoresis (SDS–PAGE) visualized with Coomassie brilliant blue (CBB) (Fig. 1d) and anti-Cre western blot (Fig. 1e). ABE8e was similarly purified through affinity chromatography on TALON metal (Fig. 1e) and 1D4 (Fig. 1f) affinity columns and completed with SEC (Fig. 1g); purity was again assessed by SDS–PAGE with CBB staining and anti-Cas9 western blot (Fig. 1h,i). PE2 was purified in a similar manner as ABE8e, with an additional heparin chromatography step after 1D4 immunoaffinity to maximize separation from impurities (Fig. 1j). We noted that ABE8e after immunoaffinity chromatography still contained contaminating nucleic acids and aggregates; these contaminants were removed effectively by SEC (Fig. 1k). We also noted that ABE8e RNP was more stable than ABE8e protein alone, as determined by differential scanning fluorimetry (DSF), especially when the single guide RNA (sgRNA) was refolded by heating and slow cooling (Fig. 1l and Supplementary Fig. 1a–f). The ABE RNP was further stabilized by 10% (w/v) sucrose. Similar to ABE8e, the PE2 RNP complex with an engineered prime-editing guide RNA (epegRNA) was more stable than PE2 protein alone; however, heat refolding of epegRNA had no additional effect on PE2 RNP stability (Fig. 1m and Supplementary Fig. 1g,h). PE RNP did not require sucrose to remain soluble. For consistency, we heat refolded all sgRNAs and epegRNAs used throughout the study. We also assessed the enzymatic activity of our ABE8e and PE2 RNPs through in vitro activity assays to ensure our proteins maintained activity following purification. ABE8e RNP displayed high levels of activity as assessed by an in vitro deamination assay (Fig. 1n,o), and PE2 RNP similarly displayed high levels of activity as assessed by an in vitro reverse-transcriptase extension assay (Fig. 1p,q).

#### CPPs enable efficient delivery of Cre in vitro and in vivo

**Fig. 2:**
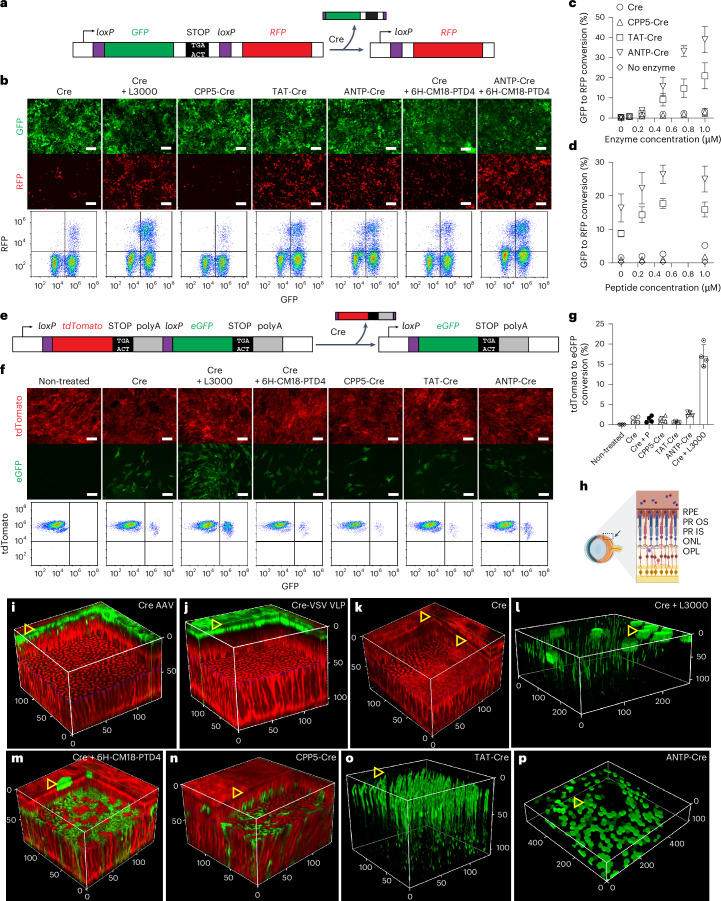
Direct protein delivery of Cre recombinase mediated by cell-penetrating peptides. **a**, Schematic cartoon of colour-switch Cre-reporter cell line. GFP is constitutively expressed, while a stop codon prevents expression of RFP. Upon Cre-mediated excision of GFP and the stop codon, RFP expression replaces expression of GFP. **b**, Various formulations of cell-penetrating Cre mediate excision of the GFP gene and induce expression of RFP measured by fluorescence microscopy (top) and flow cytometry (bottom). From left to right: purified Cre recombinase delivered alone; with Lipofectamine 3000 (L3000); with fused CPP5 covalent cell-penetrating peptide; with fused TAT; with fused ANTP; with 6×His-CM18-PTD4 non-covalent cell-penetrating peptide (6H-CM18-PTD4); with fused ANTP cell-penetrating peptide and 6×His-CM18-PTD4 peptide. Scale bar, 200 μm. **c**, Quantification of Cre-mediated GFP to RFP conversion as a function of direct protein delivery with Cre recombinase alone or with fused cell-penetrating peptides, as measured by flow cytometry. Three biological replicates with 2 analytical replicates each, mean ± s.d. 1 µM corresponds to 41.9 µg ml^−1^. **d**, Quantification of enhancement of Cre-mediated GFP to RFP conversion with increasing concentrations of 6×His-CM18-PTD4 peptide at a protein concentration of 0.5 µM. Symbols as in **c**. Three biological replicates with 2 analytical replicates each, mean ± s.d. **e**, Schematic cartoon of genetic construct of ROSA mT/mG Cre-reporter mouse model. In the mice, tdTomato is constitutively expressed, while a stop signal prevents expression of eGFP. Upon Cre-mediated excision of tdTomato and the stop signal, eGFP expression replaces expression of tdTomato. **f**, Protein delivery to skin fibroblasts isolated from the ROSA mT/mG Cre-reporter mice, as observed with a fluorescence microscope (top) and quantified by flow cytometry (bottom), 72 h post delivery. Scale bar, 100 μm. **g**, Quantification by flow cytometry of Cre recombinase delivery into ROSA mT/mG Cre-reporter mouse primary fibroblasts. Two separate experiments with 2 analytical replicates each, mean ± s.d. **h**, Schematic cartoon representing retinal cross-section orientation. RPE, retinal pigment epithelium; PR OS, photoreceptor outer segment; PR IS, photoreceptor inner segment; ONL, outer nuclear layer; OPL, outer plexiform layer. Created in part with BioRender.com: i66o107. **i**–**p**, Cre-mediated tdTomato to eGFP expression, 1 week after subretinal delivery of Cre recombinase measured by two-photon excitation microscopy. The RPE layer is orientated towards the top, denoted with open yellow triangles. Scale is provided in micrometres. **i**, AAV2/1-CMV-Cre; **j**, VSV-G pseudotyped Cre eVLP; **k**, Cre recombinase alone; **l**, Cre recombinase delivered with Lipofectamine 3000; **m**, Cre recombinase delivered with 6×His-CM18-PTD4 peptide; **n**, CPP5-fused Cre recombinase; **o**, TAT-fused Cre recombinase; **p**, ANTP-fused Cre recombinase. 3D video reconstructions of **i**–**p** are available as Supplementary Videos 1–8. Source data

We first assessed the delivery of Cre recombinase into the HEK293-loxP-GFP-RFP cultured cell line (colour switching, ‘CS’) for a rapid and convenient fluorescent readout of intracellular protein delivery. The CS reporter cell line expresses green fluorescent protein (GFP) constitutively, while a stop codon upstream of red fluorescent protein (RFP) gene prevents its expression. Upon delivery of Cre, floxed GFP-STOP is excised, enabling RFP expression in place of GFP (Fig. 2a). As a positive control, we delivered Cre recombinase as a lipoplex with Lipofectamine 3000 to confirm the specificity of this cell line, reaching up to 40% conversion of the cells (Fig. 2b). Cre recombinase alone induced low but detectable (up to 4.4%) colour switching from GFP to RFP, indicating an ability of unmodified Cre recombinase to cross cell membranes (Fig. 2b,c and Supplementary Fig. 2a,b). TAT- and ANTP-CPPs resulted in enhancement of Cre delivery (up to 28% and 45%, respectively), while the CPP5-CPP did not (Fig. 2b,c and Supplementary Fig. 2a,b). We also delivered Cre as a non-covalent complex with CPP peptide 6×His-CM18-PTD4 (ref. ^49^), which increased the delivery of Cre to ~6% and synergistically enhanced the delivery efficiency of CPP-fused Cre proteins (Fig. 2b,d, and Supplementary Figs. 3a,b and 4a,b). Next, we delivered Cre into ROSA mT/mG mice, a global fluorescent reporter strain for monitoring Cre recombinase activity^50^. All cells from the ROSA mT/mG mouse constitutively express membrane-targeted tdTomato from the *Rosa26* locus, while a polyadenylation stop signal prevents the expression of enhanced GFP (eGFP). When Cre recombinase is introduced to the cells, the tdTomato and stop signals are excised, enabling eGFP expression (Fig. 2e). We isolated and cultured primary ROSA mT/mG fibroblasts and demonstrated that Cre recombinase delivered alone resulted in low tdTomato to eGFP conversion, while Cre protein delivered by Lipofectamine 3000 resulted in higher conversion (Fig. 2f,g). In contrast to the results with the CS line, only the fused ANTP peptide modestly improved delivery of Cre recombinase to the ROSA mT/mG fibroblasts (Fig. 2f,g). When injected subretinally into ROSA mT/mG mice to evaluate in vivo activity (Fig. 2h), the positive controls, AAV1-CMV-Cre and eVLPs packaging Cre protein, mediated efficient tdTomato to eGFP conversion mainly in the retinal pigment epithelium (RPE), with occasional and scarce eGFP observed in photoreceptors, as detected by two-photon excitation (Fig. 2i,j). The untagged Cre recombinase mediated minimal eGFP expression in the RPE (Fig. 2k). Consistent with our in vitro findings, Cre recombinase delivered subretinally with Lipofectamine 3000, the 6×His-CM18-PTD4 peptide, or fused CPPs, effected ample eGFP expression in the RPE and photoreceptors (Fig. 2l–p). Notably, although not effective in vitro, CPP5-fusion peptide enabled delivery of Cre into the photoreceptors in vivo (Fig. 2n).

### CPPs are unable to efficiently deliver ABE and PE in vitro and in vivo

**Fig. 3:**
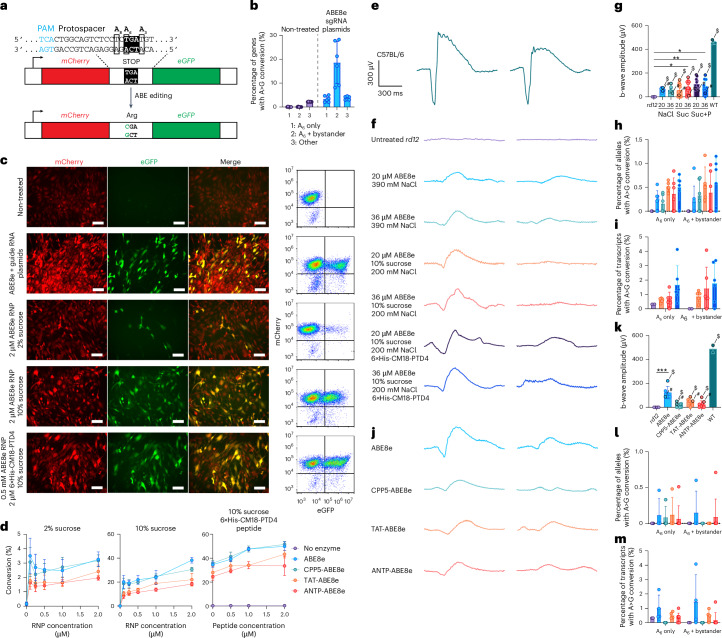
Cell-penetrating peptide-mediated delivery of ABE in vitro and in vivo*.* **a**, Schematic cartoon of fluorescent *rd12* reporter. Under the control of the CMV promoter, a gene expresses both mCherry and eGFP, with an intervening sequence from the *Rpe65 rd12* genomic sequence. The *rd12* mutation (c.130 C > T; p.R44X) prevents expression of eGFP, but successful base editing restores the reading frame and co-expression of mCherry and eGFP. **b**, Quantification of successful base editing of *rd12* reporter cells, 48 h after co-transfection of CMV-ABE8e-NG and sgRNA plasmids. Three biological replicates with 2 analytical replicates each, mean ± s.d. **c**, Assessment by fluorescence microscopy (left) and flow cytometry (right) of ABE delivery to *rd12* reporter cells, 48 h post treatment; mCherry and eGFP co-expression indicate successful delivery. Scale bar, 100 μm. **d**, Concentration dependence of efficiency of delivery of ABE in the presence of 2% (w/v) sucrose, 10% (w/v) sucrose, and of 0.5 µM ABE with 10% (w/v) sucrose and 6×His-CM18-PTD4 peptide. 1 µM corresponds to 224 µg ml^−1^. Two biological replicates with 2 analytical replicates each, mean ± s.d. **e**, ERG response curves from WT mice. **f**, ERG response curves from *rd12* mice, 2 weeks post treatment with ABE RNP in the presence of high and low NaCl and sucrose, with and without 6×His-CM18-PTD4 peptide. The left curves represent the highest-responding eye from each treatment group, while the right curves represent a low-responding eye from each treatment group. **g**, Quantification of ERG responses (b-wave amplitude) from *rd12* mice whose response curves are shown in **e**. ABE RNP concentrations are given in µM. 20 µM corresponds to 4.5 µg ABE RNP per eye, 36 µM to 8.1 µg per eye. At least 6 eyes, mean ± s.d., Kruskal–Wallis test with Dunn’s multiple comparisons test; *P < 0.05, **P < 0.01. $ # indicate data points corresponding to ERG response curves presented in panel **f**. **h**,**i**, Quantification of genomic DNA editing (**h**) and cDNA transcripts containing the edit (**i**) in ABE-treated mice whose responses are summarized in **f**. Colours are as in **g**. **j**, ERG response curves from *rd12* mice, 2 weeks post treatment with ABE RNP with or without fused cell-penetrating peptides. **k**, Quantification of ERG responses (b-wave amplitude) from *rd12* mice whose response curves are shown in **j**. $ and # indicate data points corresponding to ERG response curves presented in panel **j**.The mice received 4.5 µg ABE RNP per eye. At least 6 eyes, mean ± s.d.; Kruskal–Wallis test with Dunn’s multiple comparisons test; ***P < 0.001. **l**,**m**, Quantification of genomic DNA editing (**l**) and resulting proportion of transcripts containing the edit (**m**) in CPP-ABE-treated mice whose responses are summarized in **j**. The colours are as in **k**. Source data

**Extended Data Fig. 1:**
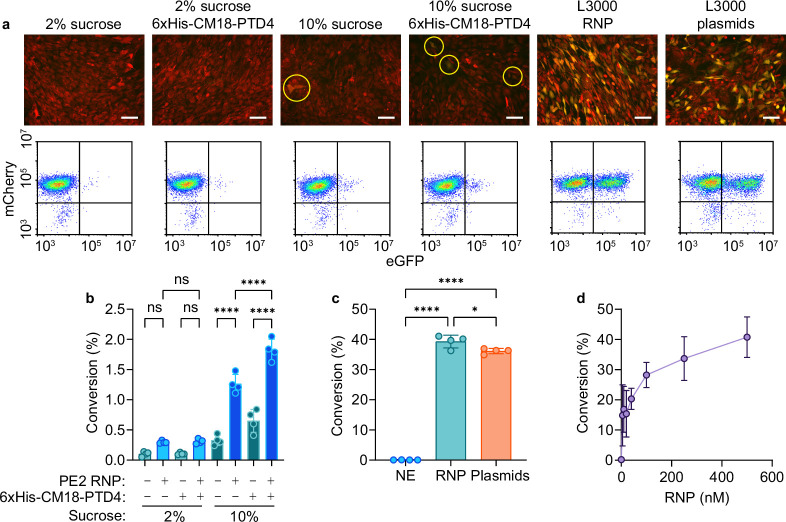
Delivery of PE RNP into *rd12* reporter cells. (**a**) Analysis of delivery of 500 nM PE2 RNP (149 µg ml^-1^), with epegRNA targeting *Rpe65 rd12*, into the *rd12* reporter cells using 2% (w/v) sucrose or 10% (w/v) sucrose, and with or without added cell-penetrating peptide 6xHis-CM18-PTD4; or using Lipofectamine 3000 (L3000). In parallel, the *rd12* reporter cells were transfected with PE2 and *rd12* epegRNA plasmids (right-most panel). The cells were analyzed using fluorescence microscopy and flow cytometry. Faint-yellow cells that were modified with PE2 RNP are highlighted with yellow circles. Scale bar: 100 µm. (**b**) Delivery of PE2 RNP using 10% sucrose and 6xHis-CM18-PTD4 peptide, quantified using flow cytometry. Two biological replicates with two analytical replicates each, mean ± s.d. One-way ANOVA with Tukey’s multiple comparisons test. ****P < 0.0001; ns, *P* > 0.05. (**c**) Comparison of efficiency of delivery of PE2 with Lipofectamine 3000 as RNP, and as a pair of PE and epegRNA plasmids. *P < 0.05; ****P < 0.0001. One-way ANOVA with Tukey’s multiple comparisons test. NE = minus enzyme control with Lipofectamine 3000 alone. (**d**) Titration of *rd12* reporter cells with increasing PE RNP and constant L3000, quantified by flow cytometry. Four biological replicates with 2 analytical replicates each, mean ± s.d. Source data

As all of the CPPs were effective to some degree in delivering Cre recombinase in vitro and in vivo, we then applied them for the delivery of ABE and PE to the *rd12* mouse model of Leber congenital amaurosis. In these mice, a nonsense mutation in *Rpe65* abolishes RPE65 expression^41,42^, leading to a lack of visual chromophore production and photoreceptor light detection; however, successful base or prime editing rescues this phenotype. To enable efficient in vitro screening, we developed a fluorescent *rd12* reporter cell line. We retrovirally transduced NIH/3T3 cells with a construct containing a fragment of the *Rpe65 rd12* complementary (c)DNA encompassing an in-frame nonsense mutation, which is flanked by mCherry and eGFP. In unedited cells, the stop codon only permits mCherry protein expression and successful transition by base or prime editing results in the expression of the mCherry-eGFP fusion protein (Fig. 3a). We verified that adenine base editing via plasmid transfection of ABE8e with a previously validated *rd12* sgRNA^42^ successfully edited the reporter construct and restored eGFP expression, as quantified by next-generation sequencing (22%, Fig. 3b) and flow cytometry (32%, Fig. 3c). Unmodified ABE applied on the *rd12* reporter cells caused low but detectable conversion of the fluorescent reporter (Fig. 3c,d), but in contrast to fusion with Cre recombinase, the fused CPP peptides did not enhance the delivery of ABE in vitro (Fig. 3d). However, as we previously observed that sucrose promoted stability of the RNP complex (Supplementary Fig. 1c–f), the addition of sucrose (10% w/v) to the RNP mixture boosted delivery of the ABE8e RNP (Fig. 3d). This was further enhanced 1.5-fold by the addition of the non-covalent 6×His-CM18-PTD4 peptide, but fused CPPs still did not result in improved delivery efficiency (Fig. 3d). In contrast to ABE, we did not observe a colour change in the *rd12* reporter cells when PE was delivered with 2% sucrose, but we noted activity when delivered with 10% sucrose. As in the case of Cre and ABE, the 6×His-CM18-PTD4 peptide modestly improved delivery efficiency of PE2 RNP, by ~1.6-fold, although overall efficiencies remained low (Extended Data Fig. 1a,b).

We then investigated whether the non-covalent 6×His-CM18-PTD4 peptide or high sucrose concentration could effectively enable the delivery of ABE8e RNP in vivo. Subretinal injection of ABE8e RNP rescued the scotopic dark-adapted flash electroretinography (ERG) b-wave, indicating successful genome editing and restoration of the visual cycle, as untreated *rd12* mice do not exhibit a recordable ERG waveform in response to this light stimulus (Fig. 3e,f). We noted that the low-NaCl, high-sucrose formulation effected a more robust rescue, as measured by ERG b-wave amplitudes (Fig. 3g). Consistent with our in vitro findings, the rescue of the scotopic flash ERG was promoted by the non-covalent 6×His-CM18-PTD4 peptide but was not substantially improved by the fused CPPs (Fig. 3f,g,j,k). We sequenced genomic DNA and transcripts isolated from RPE samples from the treated mice and noted up to 2% on-target genomic editing, corresponding to up to 7% on-target base editing in cDNA. The ABE editing outcomes were distributed approximately equally between precise edits and edits with at least one bystander adenine deaminated (Fig. 3h,i,l,m). Notably, purified RNP led to more precise ABE editing than eVLP packaging of the same ABE8e, which led to multiple deaminated adenines^28^.

### Optimized lipid nanoparticles enable the effective delivery of ABE and PE RNPs

**Extended Data Fig. 2:**
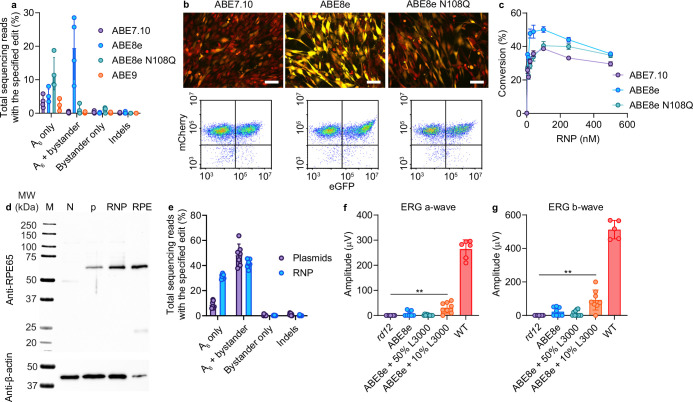
Delivery of ABE using Lipofectamine 3000. (**a**) Analysis of ABE editing of *rd12* reporter cells transfected with plasmids encoding ABE NG variants and guide RNA targeting *Rpe65 rd12*. Two biological replicates with 2 analytical replicates each, mean ± s.d. (**b,c**) ABE RNPs were delivered to *rd12* reporter cells using Lipofectamine 3000. Efficiency of delivery was analyzed using (**b**) fluorescence microscopy (100 nM, 22.4 µg ml^-1^ RNP shown here) and (**c**) flow cytometry. Scale bar: 100 µm. Two biological replicates with 2 analytical replicates each, mean ± s.d. (**d**) Rescue of RPE65 protein expression in NIH/3T3-*rd12* cells by delivery of ABE and guide RNA plasmids (p), or 100 nM ABE RNP using Lipofectamine 3000 (RNP). Mouse retinal pigment epithelium (RPE) extract was used as a positive control. M = molecular weight marker; N = untreated cells. (**e**) NGS analysis of editing of NIH/3T3-*rd12* cells treated with ABE. Two biological replicates with at least 3 analytical replicates each, mean ± s.d. (**f,g**) Rescue of visual function in *rd12* mice injected with 25 µM ABE8e RNP (5.6 µg per eye), 23.6 µM ABE8e RNP with 50%(v/v) Lipofectamine 3000 (4.5 µg per eye), or 23.6 µM ABE8e RNP with 10%(v/v) Lipofectamine 3000. Injected material additionally contained 25% (w/v) sucrose. At least 8 eyes, mean ± s.d, Kruskal-Wallis test with Dunn’s multiple comparisons test, **P < 0.01. Uncropped blots are available within Source data. Source data

As an alternative to CPPs, lipid reagents are also suitable for CRISPR/Cas9 RNP delivery^51^. Our preliminary investigations showed that binding of sgRNA by ABE results in change of the net charge from positive (zeta potential of +4.4 mV) to negative (−7.6 mV), which is in line with data published for Cas9 (ref. ^52^) and suggests that ABE RNP can be efficiently captured by cationic lipids as a lipoplex after simple mixing, or into a lipid nanoparticle after microfluidic encapsulation. For example, delivery of ABE RNP in vitro via a lipoplex with Lipofectamine 3000 was efficient in the *rd12* reporter cell line at concentrations as low as 20 nM (4.5 µg ml^−1^) (Extended Data Fig. 2b,c). ABE RNP delivered using Lipofectamine 3000 restored expression of RPE65 in the *rd12* cDNA reporter cell line, and next-generation sequencing analysis showed improved efficiency and precision of ABE editing mediated by RNP compared with plasmid (Extended Data Fig. 2d,e). However, the formulation of ABE8e RNP that was optimal in vitro (50 pmol (11.2 µg) RNP per µl of Lipofectamine 3000, 5.6 µg RNP per eye) did not restore the ERG responses in the *rd12* mice. This result might correspond to toxicity of the high dose of Lipofectamine 3000 (50% by volume, 0.5 µl per eye), as we observed substantially higher rescue when we used a 5-fold lower dose of Lipofectamine 3000 with the same amount of ABE RNP (Extended Data Fig. 2f,g). These results indicate that lipid-mediated delivery of ABE RNP in vivo is a viable approach; however, we envisioned that a chemically defined formulation that minimizes toxicity is required to maximize the potential of RNP delivery in vivo.

**Fig. 4:**
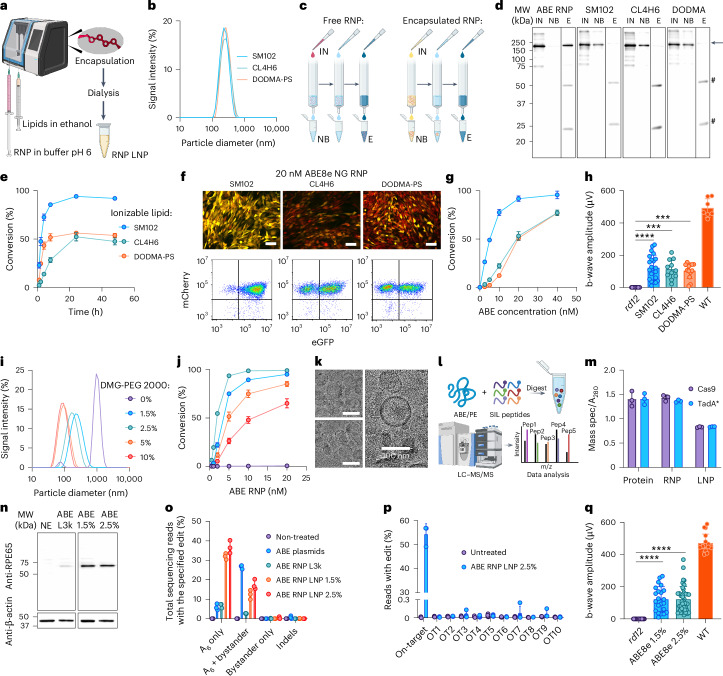
Lipid nanoparticle delivery of ABE RNPs. **a**, Schematic cartoon of encapsulation of RNP into LNP. **b**, Size-distribution analysis of ABE RNP LNP, with 1.5% DMG-PEG 2000 and ionizable lipids: SM102 (diameter *d* = 228 nm, polydispersity index PdI = 0.072); CL4H6 (*d* = 235 nm, PdI = 0.132); DODMA (*d* = 249 nm, PdI = 0.079). Averaged plots are shown, *n* = 3 replicates. **c**, Scheme of the immunoprecipitation of ABE RNP, free (red) or encapsulated into LNP (yellow), on a 1D4 resin. IN, input; NB, non-bound; E, eluate. **d**, Western blot analysis of the immunoprecipitation of ABE RNP, free and encapsulated into LNPs containing 1.5% DMG-PEG 2000 and ionizable lipids SM102, CL4H6 or DODMA. The band corresponding to ABE is indicated with an arrow. Bound material was eluted using a Laemmli sample buffer with DTT, and bands corresponding to the mouse 1D4 antibody stripped from the resin alongside ABE are indicated with hashes (#) (see also Extended Data Fig. 3a,b). **e**, Delivery of ABE RNP by LNP after incubation of the *rd12* reporter cells with the LNPs for 1–48 h, quantified by flow cytometry. The concentration of ABE RNP was 20 nM, 4.5 µg ml^−1^. Two biological replicates with 2 analytical replicates each, mean ± s.d. **f**, Fluorescence microscopy (top) and flow cytometry (bottom) results demonstrating delivery of ABE by LNP to the *rd12* reporter cells, measured 24 h after treatment. Scale bar, 100 μm. **g**, Quantification of delivery of ABE RNP as LNP after incubation of the *rd12* reporter cells with 1–40 nM ABE RNP for 24 h. Two biological replicates with 2 analytical replicates each, mean ± s.d. **h**, Summary of ERG b-wave responses of *rd12* mice treated with 1 µl ABE RNP LNP per eye. The concentration of ABE RNP in the LNP was ~2.3 µM (SM102, CL4H6, 515 ng per eye) and ~1.8 µM (DODMA, 403 ng per eye). At least 10 eyes, mean ± s.d, Kruskal–Wallis test with Dunn’s multiple comparisons test; ***P < 0.001, ****P < 0.0001. **i**, Size-distribution analysis of ABE RNP LNP with ionizable lipid SM102 and 0–10% DMG-PEG 2000. Average of 3 replicates. **j**, Delivery of ABE RNP LNP with 0–10% DMG-PEG 2000, quantified by flow cytometry. The colours are as in **i**. Two biological replicates with 2 analytical replicates each, mean ± s.d. **k**, Three representative cryoelectron-microscopic images of optimized ABE RNP LNP containing ionizable lipid SM102 and 2.5% DMG-PEG 2000. **l**, Schematic cartoon of targeted quantification of ABE and PE using mass spectrometry. **m**, MS quantification of ABE8e protein, RNP and LNP with 2.5% DMG-PEG 2000 using peptides targeting Cas9 and evolved adenosine deaminase TadA*, relative to quantification by absorbance at 280 nm. Protein concentration in LNP was estimated by correcting for dilution throughout the encapsulation and dialysis. Three analytical replicates, mean ± s.d. **n**, Rescue of expression of RPE65 in a cell line expressing *Rpe65 rd12* cDNA by ABE RNP, delivered with Lipofectamine 3000 (L3k) or via LNP containing ionizable lipid SM102 and 1.5–2.5% DMG-PEG 2000. **o**, Next-generation sequencing analysis of ABE editing outcomes in the cells with cDNA encoding *Rpe65 rd12*, treated with ABE delivered on a plasmid with Lipofectamine 3000 (L3k) or as LNP. Three analytical replicates, mean ± s.d. **p**, Off-target analysis of *Rpe65 rd12* cDNA cells. **q**, ERG b-wave responses of *rd12* mice treated with 1 µl ABE RNP LNP per eye, with ionizable lipid SM102 and 1.5 or 2.5% DMG-PEG 2000. The RNP concentrations were ~2.3 and 2.5 µM, respectively, and the doses 515 ng and 560 ng RNP per eye, respectively. At least 8 eyes, mean ± s.d, Kruskal–Wallis test with Dunn’s multiple comparisons test; ****P < 0.0001. Panels **a**, **c** and **l** created in part with BioRender.com: a74e702, m32e636, w33y755 and z14j704. Uncropped blots are available as Source data. Source data

**Extended Data Fig. 3:**
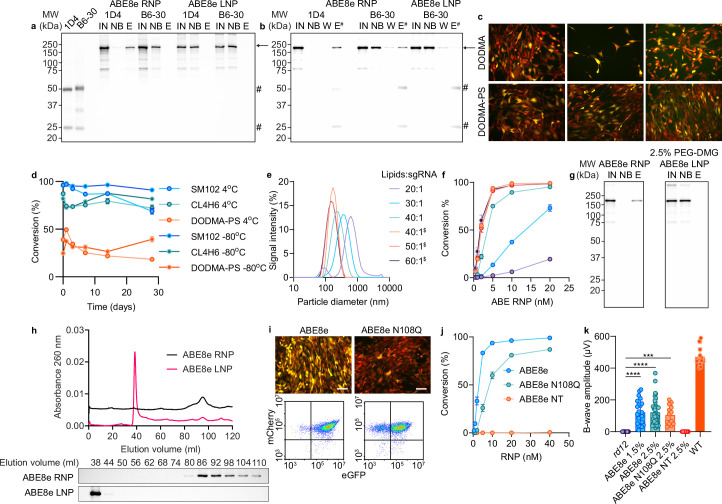
Optimization of ABE RNP LNPs. (**a,b**) Immunoprecipitation of ABE RNP, free or encapsulated in the LNP with SM102 ionizable lipid and 1.5% DMG-PEG 2000, using 1D4 resin or control B6-30 resin. Elution was done using 1 mg ml^-1^ 1D4 peptide (E) or using Laemmli sample buffer with DTT (E*). 1D4 and B6-30 resins treated with Laemmli sample buffer alone served as additional controls. The arrow (at the right) indicates the band corresponding to ABE; hash marks correspond to bands from mouse 1D4 and B6-30 antibodies detected by equine anti-mouse secondary HRP-linked antibody. (**c**) Relative cytotoxicity of LNP with ionizable lipid DODMA, with or without SOPS, demonstrated using fluorescence-microscopic images from three transfection experiments at 20 nM RNP (4.5 µg ml^-1^). (**d**) Stability (upon storage at 4 °C or -80 °C) of ABE RNP LNP made with 50% ionizable lipid and 1.5% DMG-PEG 2000, as tested with *rd12* reporter cells and 20 nM ABE RNP. Three replicates, mean ± s.d. (**e,f**) Particle size distribution and delivery efficiency of ABE RNP LNP made with various lipid:RNA weight ratios. Asterisks denote 2.5% DMG-PEG 2000; otherwise, the concentration was 1.5%. Plots shown in panels **e** and **f** represent data that were averaged from n = 3 replicates, mean ± s.d. (**g**) Encapsulation of ABE RNP into LNPs with ionizable lipid SM102 and 2.5% DMG-PEG 2000. (**h**) Size exclusion chromatograms of ABE8e RNP (black) and ABE8e RNP LNP with SM102 lipid and 2.5% DMG-PEG2000, 40:1 lipid:sgRNA ratio (red) resolved on a Sephacryl S-500 HR column (top) and anti-Cas9 blots of collected fractions (bottom). (**i,j**) ABE editing in the *rd12* reporter cells, using ABE RNP LNP visualized *via* fluorescence microscopy (here, 20 nM ABE8e RNP) and quantified by flow cytometry. Scale bar: 100 µm. Three replicates. (**k**) ERG responses of *rd12* mice treated with ABE8e LNPs made using ionizable lipid SM102 and 1.5 – 2.5% DMG-PEG 2000 (2.3 and 2.5 µM, 515 and 560 ng, respectively), ABE8e N108Q LNPs made with 2.5% DMG-PEG 2000 (2.3 µM, 515 ng), or ABE8e LNPs with non-targeting guide RNA (2.5 µM, 560 ng), 1 µl per eye. At least 8 eyes, mean ± s.d, Kruskal-Wallis test with Dunn’s multiple comparisons test, ***P < 0.001; ****P < 0.0001. Uncropped blots are available within Source data. Source data

To this end, we sought to adapt LNP technology, which has successfully delivered mRNA vaccines and Cas9 nuclease RNP^52,53^. We found that the ABE RNP was transiently stable at pH 6.0 and in 25% ethanol, conditions which are necessary for LNP formulation. To create ABE and PE RNP LNPs, we tested a panel of ionizable lipids in a prototypical lipid mixture consisting of ionizable lipid, 1,2-distearoyl-sn-glycero-3-phosphocholine (DSPC), cholesterol and 1,2-dimyristoyl-rac-glycero-3-methoxypolyethylene glycol-2000 (DMG-PEG 2000) at a molar ratio of 50:10:38.5:1.5, respectively. To avoid inactivation of ABE, we utilized ionizable lipids whose p*K*_a_ was above 6.0: 8-[(2-hydroxyethyl)[6-oxo-6-(undecyloxy)hexyl]amino]-octanoic acid, 1-octylnonyl ester (SM102): p*K*_a_ = 6.68 (ref. ^54^); 9-octadecenoic acid, 1,1′-[7-[4-(dipropylamino)butyl]-7-hydroxy-1,13-tridecanediyl] ester (CL4H6): p*K*_a_ = 6.25 (ref. ^55^); 1,2-dioleyloxy-3-dimethylaminopropane (DODMA): p*K*_a_ = 6.59 (ref. ^56^), enabling the protonation of the tertiary amine of the ionizable lipids at pH 6.0 to facilitate incorporation of ABE RNP into LNPs during encapsulation (Supplementary Fig. 5 and Fig. 4a). We noted that the resultant LNPs were highly monodispersed with a hydrodynamic diameter between 200 and 250 nm, measured by dynamic light scattering (Fig. 4b). We verified encapsulation of ABE RNP within our LNPs by distinguishing encapsulated RNPs from free ABE RNPs through immunoprecipitation with 1D4 resin and subsequent anti-Cas9 western blot analysis (Fig. 4c,d and Extended Data Fig. 3a,b). While free ABE RNPs were bound by the resin and eluted with 1D4 peptide, ABE RNP LNPs were detected in the non-bound fraction (NB). The binding of ABE was epitope specific, as a B6-30 resin directed against the N terminus of rhodopsin did not bind ABE (Extended Data Fig. 3a)^57^. When the LNPs were tested, the majority of the ABE was found in the NB and in wash fractions (data not shown). Upon elution with Laemmli sample buffer, some RNP was eluted from the 1D4 resin (Fig. 4d), suggesting non-specific binding because no material was eluted from LNP-treated resin when 1D4 peptide was used instead (Extended Data Fig. 3a), and a similar amount of LNP-derived material was eluted with Laemmli sample buffer from non-binding control B6-30 resin (Extended Data Fig. 3b).

We tested the ABE RNP LNPs on the *rd12* reporter cells and noted that the LNPs effected rapid delivery of active ABE (Fig. 4e). We also noted high conversion efficiency for all three LNPs, nearing 100% for SM102 LNPs (Fig. 4f,g). The LNPs were highly potent, with as little as 20 nM ABE RNP (4.5 µg ml^−1^) in SM102 LNPs eliciting nearly total conversion of the reporter cells. This result represented a substantial improvement over the lipoplex with Lipofectamine 3000, which peaked at ~50% of the cells converted (Fig. 4g and Extended Data Fig. 2c). We observed high toxicity of DODMA LNPs, which we alleviated by replacement of the 2.5% DSPC within the LNP with 1-stearoyl-2-oleoyl-sn-glycero-3-phospho-L-serine (SOPS) (Extended Data Fig. 3c). The LNPs remained stable for at least 28 days when stored at −80 °C, and for at least 14 days when stored at 4 °C (Extended Data Fig. 3d). We then verified that these LNPs were active in vivo via subretinal delivery into *rd12* mice, with all three LNPs effecting a substantial rescue of the ERG b-wave (Fig. 4h). Importantly, although the concentration of ABE in the LNP preparation (2 µM, 0.45 mg ml^−1^, 450 ng per eye) was at least 10-fold lower than the RNPs formulated with sucrose or Lipofectamine 3000 in vivo (20–36 µM, 4.5–8.1 mg ml^−1^, 4.5–8.1 µg per eye), we registered the most substantial response here, with the ERG b-wave amplitude reaching up to 265 µV for the SM102 LNP-RNP, whereas the highest amplitude registered for free RNP was 185 µV with added 6×His-CM18-PTD4 peptide (Fig. 3g), and 200 µV with Lipofectamine 3000 (Extended Data Fig. 2g).

Our experiments in vitro and in vivo suggested that LNPs made with SM102 were the most effective, but we reasoned that we could further improve the LNP formulation. As modulating PEG lipids can influence biodistribution^58^, we investigated the impact of varying the DMG-PEG 2000 concentration. We also investigated whether we could further optimize the lipid:RNA weight ratio, which was 40:1 in our original formulation. We found that DMG-PEG 2000 lipid is indispensable for encapsulation of the RNP, as LNPs without DMG-PEG 2000 exhibited the highest particle size (Fig. 4i) and were not active in vitro (Fig. 4j). We determined that LNPs with 2.5% DMG-PEG 2000 were the most potent in vitro, achieving nearly 100% conversion of cells at 5 nM (1.1 µg ml^−1^) ABE RNP, a concentration 4-fold lower than that for 1.5% DMG-PEG 2000 (Fig. 4j). The optimal lipid:RNA weight ratio was at least 40:1 (Extended Data Fig. 3e,f). The ABE RNP was fully encapsulated at the optimal formulation of 40:1 lipid:sgRNA ratio and 2.5% DMG-PEG 2000, as shown by 1D4 immunoassay (Extended Data Fig. 3g) and SEC (Extended Data Fig. 3h). We assessed the structure of our optimized RNP LNPs through cryoelectron microscopy (cryoEM) and confirmed intact and uniform particles with an approximate diameter of 100 nm (Fig. 4k). We further characterized our RNP LNPs by absolute quantification of ABE within the LNPs through targeted proteomics (Fig. 4l,m and Supplementary Fig. 6a–e). We noted that Cas9 and TadA* deaminase peptides were detected at the same level, indicative of intact, full-length ABE8e protein within the LNPs (Fig. 4n). The ABE RNP LNPs restored expression of RPE65 in the *rd12* cDNA cell line, and rescue via RNP was notably higher via LNP delivery compared with Lipofectamine 3000 (Fig. 4n). Next-generation sequencing revealed that the RNP LNPs mediated higher on-target editing, up to 40%, compared with plasmid- or Lipofectamine-delivered RNP treatment, and lower bystander editing compared with plasmids (Fig. 4o). The off-target activity of ABE in the *rd12* cDNA cell line was minimal, as we noted ≤0.3% edited alleles in off-target sites, concurrent with almost 60% editing of the on-target adenine (Fig. 4p). When tested in vivo, the SM102 LNP-RNP with 2.5% DMG-PEG 2000 resulted in a higher maximal rescue of the ERG b-wave compared with SM102 LNP RNPs with 1.5% DMG-PEG 2000 (368 µV and 265 µV, respectively), approaching response levels registered for WT mice (Fig. 4q).

**Fig. 5:**
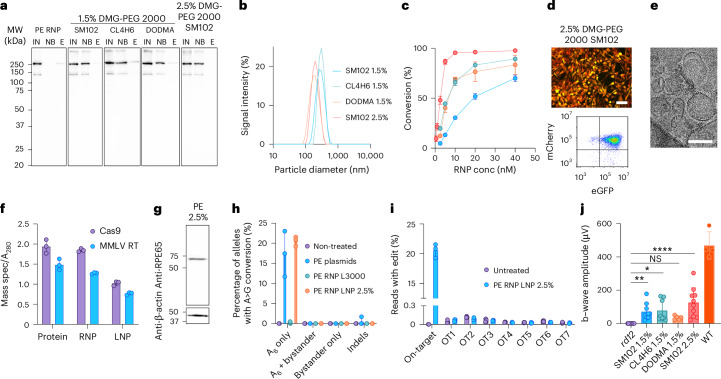
Lipid nanoparticle delivery of PE. **a**, Immunoprecipitation-encapsulation assay of PE2 RNP LNP. **b**, Particle-size distribution of PE2 RNP LNP prepared with 1.5% DMG-PEG 2000 and ionizable lipids SM102 (*d* = 270 nm, PdI = 0.068), CL4H6 (*d* = 298 nm, PdI = 0.024) or DODMA (*d* = 176 nm, PdI = 0.096), and with 2.5% DMG-PEG 2000 and ionizable lipid SM102 (*d* = 194 nm, PdI = 0.040). Average plots are shown, *n* = 3 replicates. **c**,**d**, Delivery of PE2 RNP LNP to *rd12* reporter cells (**c**), analysed by flow cytometry (bottom) and fluorescence microscopy (top) (**d**). Representative microscopic image is shown for *rd12* reporter cells treated with 20 nM PE2 RNP LNP with 2.5% DMG-PEG 2000. 20 nM corresponds to 6 µg ml^−1^. Scale bar, 100 μm. Two biological replicates with two analytical replicates each, mean ± s.d. **e**, Cryoelectron-microscopy image of PE2 RNP LNP containing ionizable lipid SM102 and 2.5% DMG-PEG 2000. Scale bar, 100 nm. **f**, Mass spectrometric quantification of PE2 as protein, RNP and LNP. Three analytical replicates, mean ± s.d. **g**, PE-mediated rescue of expression of RPE65 in a cell line transformed with cDNA encoding *Rpe65 rd12*. LNP with 2.5% DMG-PEG 2000 was used, and RNP concentration was 20 nM, 6 µg ml^−1^. **h**, Next-generation sequencing analysis of PE-editing outcome in the cells with cDNA encoding *Rpe65 rd12*. Three analytical replicates, mean ± s.d. **i**, Off-target analysis of *Rpe65 rd12* cells treated with PE RNP LNP. **j**, Restoration of visual function in *rd12* mice treated with 1.6 µM (CL4H6, 476 ng RNP per eye), 2.0 µM (SM102, DODMA, 596 ng RNP per eye) or 2.2 µM (SM102 with 2.5% DMG-PEG 2000, 655 ng RNP per eye) PE2 LNP, 1 µl per eye, as evidenced by ERG. At least 5 eyes, mean ± s.d. Kruskal–Wallis test with Dunn’s multiple comparisons test; *P < 0.05, **P < 0.01, ****P < 0.0001, ^NS^*P* ≥ 0.05. Uncropped blots are available as Source data. Source data

We also successfully encapsulated the prime editor PE2 within our optimized LNP formulation. Similar to ABE RNP, PE2 RNP can be efficiently delivered in vitro as a lipoplex with Lipofectamine 3000, although it was efficient at higher concentrations of RNP (500 nM (149 µg ml^−1^) for PE compared with 20–100 nM (4.5–22.4 µg ml^−1^) for ABE; Extended Data Figs. 1a,c,d and 2b,c). Our initial effort to encapsulate PE2 RNP into LNPs with 1.5% DMG-PEG 2000 was partially successful, as some unencapsulated PE2 protein was detected in the 1D4 immunoprecipitation assay (Fig. 5a). The particle size was also larger than for ABE RNP LNPs (Fig. 5b). Nevertheless, the unoptimized PE RNP LNPs delivered PE into the *rd12* reporter cells with efficiency surpassing Lipofectamine 3000 (Fig. 5c). By increasing the concentration of DMG-PEG 2000 to 2.5%, we no longer detected unencapsulated PE2 (Fig. 5a) and observed robust delivery of PE into the *rd12* reporter cells (Fig. 5c,d). The cryoEM images of optimized PE RNP LNP revealed the presence of homogeneous, well-defined particles (Fig. 5e). We noted that Cas9 and the reverse transcriptase (MMLV RT) were not detected at equimolar concentrations within our PE2 RNP LNPs, indicating that further improvements may be necessary in LNP formulation and in PE2 purification (Fig. 5f). Nevertheless, the PE RNP LNP restored expression of RPE65 in the cDNA-expression cell line (Fig. 5g). Next-generation sequencing revealed that RNP-mediated prime editing led to exceptional purity of editing outcomes, as we did not notice any unwanted editing of the cells bearing the *Rpe65 rd12* cDNA, while indels were detected in cells transfected with PE and epegRNA plasmids (Fig. 5h). No off-target editing occurred in the cells treated with PE RNP LNP (Fig. 5i). Importantly, using PE2 RNP LNPs, we achieved a magnitude of rescue of the ERG b-wave in *rd12* mice comparable to that of ABE RNP LNPs, with ERG b-wave amplitudes exceeding 300 µV (Fig. 5j).

### In vivo ABE and PE editing in the *rd12* mouse model restores visual chemistry and physiology

**Fig. 6:**
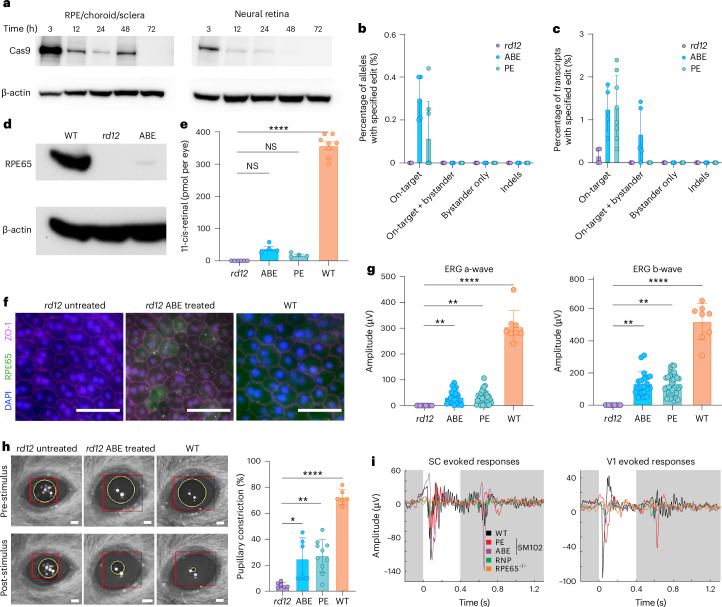
In vivo physiology and chemistry of *Rpe65* correction. **a**, ABE RNP LNP degradation kinetics after subretinal injection into WT mice; anti-Cas9 western blot analysis of RPE/choroid/sclera and neural retina lysates collected at the indicated hours post injection. **b**,**c**, Next-generation sequencing of genomic DNA (**b**) and of transcripts (**c**) to document *Rpe65*-editing outcomes after treatment with ABE RNP LNP or PE RNP LNP. **d**, Anti-RPE65 western blot analysis of RPE/choroid/sclera lysates from WT, untreated *rd12* and ABE-RNP-LNP-treated *rd12*. **e**, High-performance liquid chromatography (HPLC) quantification of 11-*cis*-retinal in whole eyes from dark-adapted untreated *rd12* mice, ABE-RNP-LNP- and PE-RNP-LNP-treated *rd12* mice and WT mice. **f**, RPE flatmounts of *rd12* untreated, ABE-RNP-LNP-treated *rd12* and WT mice stained for RPE65 (green) and counterstained with ZO-1 (magenta) and DAPI (blue). Scale bar, 50 µm. **g**, Scotopic flash ERG a-wave (left) and b-wave (right) amplitudes for *rd12* mice treated with ABE RNP LNP or PE RNP LNP, compared to untreated *rd12* and WT mice. **h**, PLR after a 10^1.2^ W m^−2^ stimulus for ABE-RNP-LNP- and PE-RNP-LNP-treated *rd12* mice, compared to untreated *rd12* and WT mice. Data quantified as pupil diameter constriction post stimulus compared to pupil diameter pre-stimulus in dark-adapted animals. Representative frames (left) and summarized data (right). Scale bar, 1 mm. **i**, Representative SC (left) and V1 (right) responses from WT mice (black), *rd12* mice treated with free ABE RNP (green), ABE RNP LNP (purple), or PE RNP LNP (red) and untreated *rd12* mice (orange). The mice received 1 µl of 2.5 µM ABE RNP (560 ng) or 2.2 µM PE RNP (655 ng) per eye. All data plotted as mean ± s.d. Data in **e** and **g** were analysed using one-way analysis of variance (ANOVA) with Kruskal–Wallis test; in **h**, using one-way ANOVA with Dunnet’s multiple comparisons test; *P < 0.05, **P < 0.01, ***P < 0.001, ****P < 0.0001, ^NS^*P* ≥ 0.05. Uncropped blots are available as Source data. Source data

**Extended Data Fig. 4:**
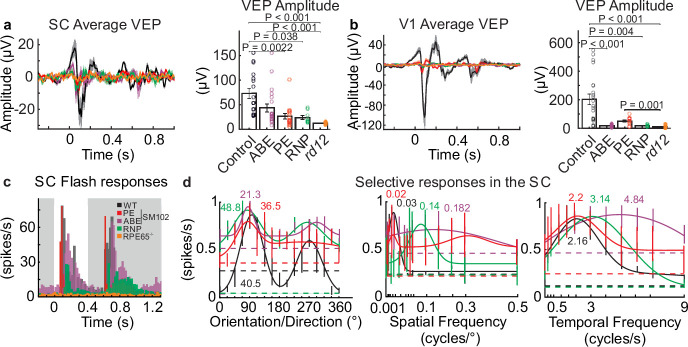
Treatment with editor-containing nanoparticles restores visual responses in *rd12* mice. (**a**) Visually evoked potentials (VEPs) in the superior colliculus (SC) for untreated WT mice (black); *rd12* mice treated with free RNP (green); *rd12* mice treated with ABE RNP LNP (purple); *rd12* mice treated with PE RNP LNP (red); and untreated *rd12* mice (orange). Bar plots represent the summary of the VEPs from all tested animals. (**b**) Same analysis as in (A) performed for the primary visual cortex (V1). (**c**) Overlapped examples of histograms for single-neuron flash responses. (**d**) Single-cell examples of selective neurons recorded in the SC. The tuning curves present orientation-selective cells, spatial frequency, and temporal frequency tuning curves for the tested mice. Numbers on the graphs indicate HWHH, optimal spatial frequency, and optimal temporal frequency. Source data

After we characterized the LNPs in vitro and screened them in vivo for the rescue of ERG response in the *rd12* mice, we applied the optimized RNP LNPs to investigate the physiological rescue of the *rd12* inherited retinal degeneration phenotype. As transient exposure to genome-editing RNPs improved our editing purities in vitro, we sought to determine the residence time of ABE within the mouse eye. We injected the optimized ABE RNP LNP with SM102 and 2.5% DMG-PEG 2000 subretinally into WT mice and killed individual mice at sequential intervals to determine the kinetics of ABE degradation. We noted that ABE was detectable up to 24 h post injection in the neural retina and up to 48 h in the RPE (Fig. 6a). We then delivered our optimized ABE RNP LNP via subretinal injection into 3–4-week-old juvenile *rd12* mice. Two weeks after injection, we noted an average of 0.30% precise correction of *Rpe65* genomic DNA by ABE and an average of 0.12% precise correction by PE (Fig. 6b), as well as an average of 1.25% precise correction of *Rpe65* transcripts by ABE and 1.28% by PE (Fig. 6c). We noted 0.67% bystander editing by ABE in the transcripts, with no indels with either ABE or PE. The base editing efficiency was similar in eyes treated with ABE RNP LNP with 1.5% DMG-PEG 2000 (not shown). We did not detect off-target editing (Supplementary Fig. 7a,b), and we did not note any editing when ABE and PE RNPs were complexed with guide RNAs that did not target the *Rpe65* locus (Supplementary Fig. 8a,b). We also detected restoration of RPE65 in the RPE of the *rd12* mice treated with ABE RNP LNP, according to western blot (Fig. 6d) and immunostaining of RPE flatmounts (Fig. 6f). Restoration of RPE65 function would lead to the production of 11-*cis*-retinal, the chromophore for photoreceptor-mediated vision. Accordingly, we assayed the retinoids in the whole eyes and detected 11-*cis-*retinal only in *rd12* mice that were treated with either ABE or PE RNP LNPs (Fig. 6e). When we performed scotopic flash ERG on the treated *rd12* animals, we observed rescue of both the a- and b-wave ERG amplitudes, both of which were not detectable in untreated *rd12* animals (Fig. 6g). Again, we noted no rescue of the ERG flash response upon treatment with ABE and PE RNPs with non-targeting guides (Supplementary Fig. 8c,d). We also demonstrated restoration of intact visual signalling from the eye through the optic nerve. The pupillary light reflex (Fig. 6h) and evoked responses from the super colliculus (SC) and primary visual cortex (V1) (Fig. 6i and Extended Data Fig. 4), which both require 11-*cis*-retinal and intact neuronal connections, were restored in *rd12* mice treated with ABE or PE RNP LNP. These results collectively demonstrate that a single dose of ABE or PE RNP LNPs can correct the *Rpe65 rd12* mutation and partially restore normal physiology and biochemistry in the *rd12* eye. Further development of ABE and PE RNP geared towards increased editing efficiency will result in a blueprint for chemically defined, effective RNP LNP formulations that allow for the repair of genetic mutations causing dysfunction of the RPE and other tissues.

## Discussion

Genome-editing technologies have opened new avenues in gene therapy, offering potential strategies for addressing both genetic and non-genetic diseases^6,59,60^. Simultaneously, the rapid advances in genome editing have illuminated the critical necessity for precise, safe and efficient delivery systems. Traditionally, vectors including AAV and LV have been the standard approaches in this domain. However, a growing body of evidence highlights intrinsic limitations associated with these vectors. For AAV vectors, one limitation is their constrained packaging capacity. AAVs can typically accommodate genomes ~4.7 thousand base pairs long, which can be restrictive, especially when attempting to deliver larger genome-editing constructs such as base editing and prime editing systems^61–63^. This limitation necessitates either the truncation of essential elements, less active homologues, or the use of dual-vector strategies, which can reduce efficiency and increase complexity. Lentiviral vectors, while accommodating larger genetic payloads, have concerns related to their integration into the host genome^64^. Such integrations, although ensuring long-term expression, can disrupt endogenous genes or regulatory elements, leading to potential genotoxicity^65–67^. Unintended insertions can also potentially activate oncogenes or deactivate tumour suppressor genes, escalating the risk of malignancies^68,69^. Moreover, both AAV and lentiviral vectors can induce immune responses^70–74^. The pre-existing immunity to certain AAV serotypes in the population can render AAV-mediated therapies less effective or even elicit adverse immune reactions and prevent redosing^72^. Similarly, lentiviral vectors can trigger cellular immune responses against transduced cells, impacting the longevity and efficacy of the therapeutic intervention. These challenges underscore the necessity to explore alternative delivery systems that can bypass the constraints and risks associated with AAV and LV. Consequently, non-viral delivery methods such as LNPs, silica nanocapsules, eVLPs and RNP delivery have been developed^28,35,40,75–81^. A major advantage of the approach we propose here is the pharmacologically defined formulation of the RNP LNPs, which offer the most transient exposure to genome-editing agents. Before progressing into clinical trials, a reproducible and manufacturable system will be required to maximize patient safety and satisfy regulatory requirements. Compared with viral and viral-derived systems, such as AAV and eVLPs, an LNP approach allows for a controllable synthetic therapeutic strategy with definable components, and LNPs could offer more uniform and reproducible synthesis compared with previously described lipoplex formulations which deliver RNPs^82^.

In this study, we have developed methods that utilize CPPs and LNPs to deliver proteins and ribonucleoprotein complexes. CPPs, when used as an excipient rather than fused to the protein, improved delivery of ABE RNP. The amphiphilic, cationic CPP can bridge the interactions between RNP and cell surface receptors and proteoglycans, improve absorption of ABE RNP by the cells^49,83,84^ and circumvent intracellular challenges such as endosomal escape. While encouraging, the CPP approach had solubility and efficiency issues and was ineffective for PE RNP. We decided to use protein–lipid and RNA–lipid interactions to bring RNPs into the cell using LNP technology, and we ultimately delivered ABE8e and PE2 RNPs to correct the *rd12* mutation in vitro and in vivo. ABEs have previously been delivered in vitro in a variety of cell lines and in vivo into the RPE and other tissues as a lipoplex^35,37,38^, but the cellular toxicity associated with Lipofectamine-type reagents used to form the lipoplexes suggests that less toxic LNPs could improve the functional outcome of RNP-mediated editing^85^.

Our results show that ABE8e RNP is a useful testing system for RNP-delivery technology, and the technology developed for ABE RNP delivery can be rapidly adapted for PE. We imagine that this approach could be further extended to cytosine base editors, as encapsulation is dependent primarily on the negatively charged guide RNA. When compared head-to-head in vivo in the *rd12* mouse, base editing was more efficient at the target base than prime editing, while prime editing resulted in pure editing outcomes. Consequently, the higher efficiency of ABE means it will be favoured over PE if bystander editing does not result in deleterious coding changes. In general, optimization of transient ABE delivery could be more straightforward than that of PE due to the lower complexity and higher activity of ABE. We demonstrated that the precision of base editing can be further improved by using more precise, albeit less active variants, for example, ABE8e N108Q (Extended Data Figs. 2a and 3i–k). However, PE is still indispensable as it can perform edits that base editors cannot, including modification of sites containing clustered adenines or cytosines. Our study utilized a PE2 editing strategy without the introduction of a second guide RNA that programmes a nick on the unedited strand, the PE3 strategy, which in general increases prime editing efficiency. Similar to the eVLP system, LNPs allow RNPs with multiple guide RNAs to be packaged, theoretically allowing for PE3 (ref. ^52^). However, it is unclear whether adopting PE3 would improve editing outcomes without unacceptable indel formation.

Our study has identified several challenges associated with the LNP delivery system. One of the primary concerns centres on the low concentration of the active macromolecule. Furthermore, the lipid content, which exceeds the weight of the ribonucleoprotein complex by approximately eight times, could pose potential translational challenges in non-human primates or humans, and optimization of the LNP to increase payload delivery without inducing immune response may be required. Overall, optimizing the formulation of LNPs and their characteristics is of paramount importance for enhancing delivery efficiency, especially for challenging target tissues such as the RPE. The RPE has specific structural and physiological attributes that can impact the effectiveness of LNP-mediated delivery. Reducing the size of LNPs has been highlighted as a potential strategy to enhance their uptake by cells. Smaller nanoparticles, typically below 100 nm in diameter, have been shown to be more readily internalized by cells, possibly due to more efficient endocytosis^86^. For the RPE, with its tight junctions and unique phagocytic role, smaller LNPs may offer better uptake and retention to maximize LNP delivery. Enhancing the functionality of LNPs is another potential approach^79^. Incorporating specific ligands or targeting moieties that recognize and bind to receptors on the RPE cells could improve LNP uptake^87^. Recent studies have demonstrated the effectiveness of peptides and small molecules that target RPE-specific receptors and increase the internalization of nanoparticles^77,88^. By tailoring the surface properties and composition of LNPs, it could be feasible to exploit the unique biology of the RPE to enhance delivery.

Despite the challenges with the LNP delivery system, our study presents encouraging functional outcomes. While the observed editing efficiency of *Rpe65* in *rd12* mice was lower than previously reported, a noteworthy restoration of retinal function was evident, as demonstrated by the ERG recordings. Several factors potentially underlie this functional improvement despite the modest genomic correction. A mechanism to consider is the interplay between mRNA transcribed from the corrected *Rpe65* allele and that from the mutant *Rpe65* allele harbouring a nonsense mutation. It is well established that mRNA molecules containing premature termination codons are often subjected to nonsense-mediated decay, a surveillance pathway that degrades aberrant mRNA transcripts^89^. Thus, mRNA transcribed from the mutant *Rpe65* allele could be selectively targeted and degraded by the nonsense-mediated decay pathway, leading to its reduced levels in the RPE. Concurrently, mRNA derived from the corrected *Rpe65* allele would remain stable and accumulate, effectively compensating for the diminished mutant transcript levels. However, additional experiments are needed to correlate functional *Rpe65* mRNA levels with the zygosity of corrected alleles of *Rpe65* in *rd12* mice.

Supporting evidence from our previous investigation provides additional insight into the functional implications of our current findings. In a previous study, we characterized a novel animal model, the RPE65-P2A-CreERT2 knock-in mouse. Remarkably, the homozygous knock-in mice, despite expressing RPE65 at levels amounting to only 1–5% of their WT counterparts, exhibited ERG responses that were nearly indistinguishable from the WT mice^90^. The editing in our current study also resulted in the partial restoration of vision-dependent outcomes, such as the pupillary light reflex and V1 and SC responses, which are all dependent on visual input to the brain. This observation suggests that achieving high levels of RPE65 correction and expression would not be required for fully restoring retinal function, but a low yet stable level of RPE65 could suffice to produce clinically relevant outcomes. In essence, while the genomic-correction efficiencies mediated by ABE or PE LNP could be further optimized, restoration of retinal function could be achieved at low editing rates. The nuanced interplay between genomic editing, mRNA stability and functional restoration provides a compelling rationale for achieving even modest genomic corrections with strategically targeted editors, as they could bring about meaningful therapeutic benefits.

A fundamental gap in our understanding of the cellular uptake and processing mechanisms for LNPs and RNPs limits the efficiency of these systems. Clarifying these mechanisms is crucial for enhancing delivery efficiency. The discrepancies in the delivery efficiencies of Cre, ABE and PE within our study suggests that optimizing the size of the encapsulated protein, the overall diameter of the LNP, the length of the guide RNA and the surface charge of the protein are crucial factors for improving delivery efficiency. In addition to these physical and chemical optimizations, the development of newly evolved genome editors offers further potential to enhance editing efficiency. These advanced editors can be specifically tailored for target-sequence specificity, providing a refined approach to genome modification with potentially higher efficacy and precision.

In summary, our results highlight the potential of LNP-mediated delivery of RNPs as an attractive alternative to viral delivery methods in genome-editing applications. We and others previously showed the potential of RNP delivery via eVLP, lipoplex or RNP with Lipofectamine-type cationic lipids. Here we have described chemically defined and adjustable LNPs that are highly reproducible and stable. Although the RNP LNPs will require additional optimization, including fully quantitative characterization of encapsulation efficiency, before advancing to good manufacturing practice production for non-human-primate and human trials, our approach offers major safety and manufacturing advantages. Future endeavours should focus on refining the LNP formulation to enhance delivery and editing efficiency, particularly to target tissues selectively while also seeking a deeper understanding of the functional consequences of genome editing. There is also a need to develop novel ionizable lipids that would efficiently deliver the RNP with reduced risk of inflammation. This multifaceted approach will be instrumental in harnessing the full potential of genome-editing technologies and in ensuring their safe and effective clinical translation.

## Methods

### Animals

Pigmented Gt(ROSA)26Sor^tm4(ACTB-tdTomato,-EGFP)Luo^ (ROSA^mT/mG^) mice were purchased from the Jackson Laboratory (JAX 007676) and crossed with BALB/cJ albino mice (JAX 000651) to establish an albino ROSA^mT/mG^ line, referred to as ROSA mT/mG mice. The C57BL/6J (‘WT’, JAX 000664) and *rd12* (JAX 005379) mouse lines were purchased from the Jackson Laboratory and housed in the vivarium at the University of California, Irvine, where they were maintained on a normal mouse chow diet and a 12 h/12 h light/dark cycle. All animal procedures were approved by the Institutional Animal Care and Use Committee of the University of California, Irvine, and were conducted in accordance with the NIH guidelines for the care and use of laboratory animals, and with the Association for Research in Vision and Ophthalmology Statement for the Use of Animals in Ophthalmic and Visual Research.

### Molecular cloning

The N-terminal Cre recombinase fusion proteins were produced from recombinant plasmids based on pTAT-Cre^46^. pTAT-Cre was purchased from Addgene (35619), and pCPP5-Cre and pN-Cre inserts were synthesized and cloned by GeneWiz and cloned into pET-28b (Supplementary Information, Plasmid DNA sequences). The antennapedia (ANTP) DNA sequence was generated by single-strand oligo annealing, using ANTP-Or_F and ANTP-Or_R primers (Supplementary Table 1). The two oligonucleotides were mixed at a 1:1 molar ratio for a total DNA concentration of 80 µM in 50 mM Tris-HCl, pH 7.4, 62.5 mM NaCl and 10 mM ethylenediaminetetraacetic acid (EDTA). The annealing was done in a thermocycler with an initial heating step of 2 min at 95 °C and gradual cooling for 45 min to 25 °C. The pANTP-Cre plasmid was obtained by replacing the TAT sequence with ANTP between the NcoI and NdeI cloning sites. The plasmids were propagated in *Escherichia coli* NEB-5α cells (New England Biolabs, C2987H). To produce a positive control Cre eVLP, Cre (Addgene, 123133) was subcloned into pCMV-MMLVgag-3xNES-ABE7.10-NG (Addgene, 181753) via USER cloning (NEB), and eVLPs were prepared as previously described^28^.

Genes encoding ABE7.10, ABE8e and PE2 constructs were subcloned into pRha rhamnose-inducible expression vectors via USER cloning (NEB). The 1D4 peptide with a preceding tobacco etch virus (TEV) protease recognition site was introduced by PCR site-directed mutagenesis and KLD cloning (NEB, M0554S). Sequences encoding cell-penetrating peptides were introduced into ABE using PAGE-purified single-strand oligonucleotides (Sigma-Aldrich). The oligonucleotides were annealed at a final concentration of 25 µM in 10 mM Tris-HCl pH 8.0, 50 mM NaCl, 1 mM EDTA and 5 mM MgCl_2_ by heating at 95 °C for 5 min and slow cooling in a thermocycler. Assembled duplexes were ligated at a 100-fold excess into the protein expression vectors linearized with FastDigest NdeI (Thermo Fisher, FD0584). The ligation mixtures were transformed into NEB-5α cells and plated onto LB-agar plates with 25 µg ml^−1^ kanamycin. Candidate clones were identified using colony PCR and confirmed by Sanger sequencing (GeneWiz).

### Expression and purification of Cre recombinase

The plasmids were transformed into *Escherichia coli* BL21star (DE3) (Thermo Fisher, C601003), and the selected clones were grown in Terrific Broth (TB; Thermo Fisher, 22711-022) with 50 µg ml^−1^ kanamycin (Goldbio, K-120-SL25) overnight at 37 °C with mixing at 190 r.p.m. The production cultures were inoculated with overnight cultures and incubated at 37 °C with mixing at 190 r.p.m. After absorbance at 600 nm (A_600 nm_) reached 0.5, protein expression was induced with 0.5 mM isopropyl β-d-1-thiogalactopyranoside (IPTG; Goldbio, I2481C25) and incubation was continued at 20 °C for 16 h. The cells were collected by centrifugation at 7,000 *g* at 4 °C for 10 min and the pellets were stored at −80 °C until used further for protein purification.

Cre recombinase was kept on ice or refrigerated in a cold room at 4–8 °C throughout the purification procedure. The cell pellet from a 1.5-l culture was thawed in a room-temperature water bath, resuspended in the Cre-lysis buffer (50 mM Na phosphate pH 7.4, 1 M NaCl, 1 cOmplete EDTA-free protease inhibitor cocktail tablet (MilliporeSigma, COEDTAF-RO) per 50 ml buffer) and lysed by sonication (125-W pulses (5 s on, 5 s off) for 10 min total) or French press (3 passes at up to 15,000 psi). The lysate was centrifuged at 48,500 *g* for 15 min and incubated with 1 ml of a suspension of Ni-Sepharose High Performance beads (Cytiva, 17526801) in a rotating mixer for 1 h. The resin was centrifuged at 500 *g* for 5 min, washed with 40 ml of the Cre-wash buffer (25 mM Na phosphate pH 7.4, 500 mM NaCl), centrifuged again, packed in a Tricorn 5/50 column, connected to a Bio-Rad DuoFlow system (Bio-Rad) and perfused at 0.5 ml min^−1^. The resin was washed with 20 ml of the Cre-wash buffer or until a stable absorbance baseline was observed; then, the proteins were eluted with 30 ml of a continuous gradient of the Cre-elution buffer (25 mM Na phosphate, pH 7.4, containing 500 mM NaCl, 500 mM imidazole). The fractions containing Cre recombinase that were identified by SDS-PAGE and CBB staining (Quick Coomassie stain, Anatrace, GEN-QC-STAIN-1L) were concentrated and subjected to size exclusion chromatography on a Superdex 200 Increase 10/300 GL column (Cytiva, 28990944) or a HiLoad 16/600 Superdex 200 pg column (28989335), with P500G buffer (20 mM sodium phosphate, pH 7.4, 500 mM NaCl, 20% (v/v) glycerol) as the mobile phase. Fractions containing pure Cre recombinase were concentrated, snap frozen in liquid nitrogen and stored at −80 °C.

### Expression and purification of ABE and PE

*E. coli* BL21star (DE3) cells (Thermo Fisher, C601003) were transformed with the expression plasmids and grown overnight on Luria-Bertani (LB)-agar plates with 25 μg ml^−1^ kanamycin; single clones were used to inoculate starter cultures in TB with kanamycin and grown overnight. One and a half litre of TB with kanamycin was inoculated with 10 ml of starter culture, and the cells were grown at 37 °C with mixing at 190 r.p.m. until they reached an A_600nm_ of 1.5. Then, the cultures were cooled in an ice-water slurry for 30 min to 1 h, and protein expression was induced with 0.8% (w/v) rhamnose (Goldbio, R-105-250). The proteins were expressed at 17 °C with mixing at 190 r.p.m. for 16–24 h. The cells were collected by centrifugation at 5,000 *g* for 10 min at 4 °C and the pellets were stored at −80 °C.

All protein purification steps were conducted in a cold room (~4–8 °C) or on ice. The thawed cells from a 1.5-l culture were homogenized with a 40 ml Dounce homogenizer in lysis buffer (100 mM Bis-Tris propane, pH 8.0, 1 M NaCl, 20% (v/v) glycerol, 5 mM tris(2-carboxyethyl)phosphine (TCEP), 1 cOmplete Ultra EDTA-Free protease inhibitor tablet per 40 ml of the buffer) and lysed by sonication with a Qsonica Q125 sonicator (125 W) with a 1/8-inch microtip at 100% amplitude for a total of 20 min (intermittent pulses: 5 s on, 5 s off). The lysate was clarified two times at 4 °C by centrifugation at 48,500 *g* for 10 min. ABE was captured on a 3-ml TALON metal-affinity resin (Takara Bio, 635502). The resin was then washed with 100 ml of the lysis buffer without inhibitors and with 10 ml of the wash buffer (100 mM Bis-Tris propane, pH 8.0, 500 mM NaCl, 20% (v/v) glycerol, 1 mM TCEP). The proteins were eluted with the wash buffer supplemented with 150 mM imidazole.

In a second step, ABE was purified by immunoaffinity chromatography using a 1D4 resin (Sepharose CL-4B resin; Cytiva, 17043001) with immobilized 5–10 mg ml^−1^ 1D4 antibody purified in house. The 1D4 resin (4 ml) was packed in a DWK Life Sciences Kimble Kontes FlexColumn. The column was equilibrated with the wash buffer (described above). ABE was loaded by gravity flow. The column was washed with at least 40 ml of the wash buffer at 0.5 ml min^−1^, and then ABE was eluted with 1 mg ml^−1^ 1D4 peptide in the wash buffer at ~1 ml h^−1^. Fractions containing ABE were pooled, concentrated and further purified to remove contaminating nucleic acids and aggregates, by size exclusion chromatography on a Superdex 200 Increase 10/300 GL column or a Superdex 200 16/60 Prep Grade column. The protein was eluted with the ABE storage buffer (10 mM 4-(2-hydroxyethyl)-1-piperazine ethanesulfonic acid (HEPES), pH 7.0, 500 mM NaCl, 20% glycerol). The fractions containing pure ABE were concentrated using Amicon Ultra centrifugal filters with 30-kDa molecular weight cut-off membranes (Merck, UFC903024), sterilized by passage through 0.22-μm filters, quantified using UV absorption at 280 nm, divided into aliquots and snap frozen in a chilled metal block for storage at −80 °C.

PE was purified using a similar procedure as for ABE. Cells expressing PE were lysed using a single pass through the French press and subsequent sonication for 10 min. Metal affinity and immunoaffinity steps were identical as for ABE, except that the wash buffer contained 400 mM NaCl. After immunoaffinity chromatography, PE was subjected to heparin-affinity chromatography (HiTrap Heparin HP 5 ml; Cytiva, 17040703) at 0.5 ml min^−1^. The heparin column was equilibrated with the wash buffer containing 400 mM NaCl. After PE was loaded, the column was washed with at least 50 ml of the wash buffer until the UV baseline was stable, and then bound proteins were eluted with a 200-ml NaCl gradient (0.4 M–1.0 M). Purest fractions were selected for size exclusion chromatography on a Superdex 200 16/60 Prep Grade column, with PE-storage buffer (10 mM HEPES, pH 7.0, 500 mM NaCl, 5% (v/v) glycerol) as the eluent. Pure PE was concentrated, filtered, quantified using UV absorption at 280 nm, distributed into aliquots and snap frozen for storage at −80 °C.

The protein purity was assessed using SDS–PAGE in hand-cast Tris-glycine-SDS discontinuous gels with 4% acrylamide in a stacking gel (pH 6.8) and 10% acrylamide in a resolving gel (pH 8.8) with 2.7% crosslinker (acrylamide:bis-acrylamide ratio of 37.5:1; Bio-Rad, 1610158). The samples were mixed with 4×-concentrated Laemmli sample buffer (Bio-Rad, 1610747) and supplemented with 50 mM dithiothreitol (DTT; MilliporeSigma, D9779), denatured at 70 °C for 10 min and centrifuged before applying on gel. The protein concentration was quantified using UV absorption spectroscopy by measuring absorbance at 280 nm with a Nanodrop ND-1000 spectrophotometer. Separation of contaminating nucleic acids was followed by monitoring the ratio of absorbance at 260 nm to absorbance at 280 nm; a ratio <0.60 was used as an indication of protein free from nucleic acids. A typical A_260 nm_/A_280 nm_ value for purified ABE and PE was 0.55. Extinction coefficients of the constructs were estimated using the ProtParam tool (https://web.expasy.org/protparam/).

### ABE and PE activity assay

Synthetic 60-bp-long DNA oligonucleotides (Sigma) labelled with fluorescein on the strand undergoing deamination were annealed at a 1:1 ratio in 10 mM Tris pH 8.0, 50 mM NaCl and 1 mM EDTA by incubation at 95 °C for 5 min and subsequent slow cooling to 20 °C. End-modified sgRNA and epegRNA were ordered from IDT Technologies, with 2′-*O*-methyl groups on the first three and last three nucleotides, and the first and last three phosphodiester bonds were replaced with phosphorothioate bonds. The guide RNAs were dissolved in nuclease-free water at 37 °C for 15 min at 500 r.p.m. in a thermomixer and folded by incubation at ~75 °C for 5 min, followed by slow cooling. Prepared nucleic acids were quantified using UV absorption spectroscopy by measuring absorbance at 260 nm. Accordingly, an A_260nm_ value of 1.0 corresponded to a DNA concentration of 50 µg ml^−1^ or an RNA concentration of 40 µg ml^−1^. DNA was stored at −20 °C and RNA at −80 °C.

For the ABE enzymatic assay, ABE ribonucleoprotein was assembled by incubation with 1.5-fold molar excess of sgRNA in a reaction buffer (20 mM Bis-Tris propane, pH 7.5, 100 mM KCl, 2.5 mM MgSO_4_, 2 mM DTT, 5% (v/v) glycerol) for 15 min at room temperature. Additional 10% (w/v) sucrose was used when RNPs were assembled above 4 µM. Then, ABE was diluted to 1 µM with the reaction buffer, preheated at 37 °C and added with 15 ng of DNA substrate (0.02 µM final). The deamination was conducted for 10–60 min for ABE8e. The 20-µl reactions were quenched by addition of 30 µl of water preheated to 95 °C and incubated for 2 min at 95 °C. After cooling, the mixtures were treated with 1 µl of RNase A (20 mg ml^−1^) and 1 µl of proteinase K (20 mg ml^−1^) for 15 min at room temperature; then DNA products were purified using an Oligo Clean and Concentrator kit (Zymo Research, D4061). Purified DNA was nicked with 5 units of Endonuclease V (EndoV, NEB) for 2–3 h at 37 °C, after which the reaction was quenched by addition of TriTrack DNA-loading dye (1× final) (Thermo Fisher, R1161) and incubation at 95 °C for 2 min. The cleavage products were analysed by denaturing polyacrylamide gel electrophoresis with urea (Urea–PAGE) in Bio-Rad MiniProtean continuous hand-cast 15% acrylamide gels in Tris-borate-EDTA (TBE; Bio-Rad, 1610770) with 7 M urea and 5% crosslinker (acrylamide:bis-acrylamide ratio of 19:1; Bio-Rad, 1610144). The voltage was controlled to maintain at least 42 °C in the electrophoresis chamber. Imaging was done using the ChemiDoc MP system (Bio-Rad). For fluorescein, it was done immediately after electrophoresis and for the SYBR Gold (Thermo Fisher, S11494), after 30 min staining with 1× dye in 1×TBE.

For the in vitro reverse-transcriptase elongation assay of PE, the activity assay was carried out in the same reaction buffer as for ABE, supplemented with 0.5 mM deoxyribonucleotides (dNTP). PE2 RNP was assembled with 1.1-fold molar excess of epegRNA, targeting the rd12 locus in the reaction buffer without added sucrose for 15 min at room temperature. Then, reactions were preheated at 37 °C and 45 ng of fluorescein-labelled substrate was added to a final volume of 15 µl. After 15 min, the reactions were quenched with 1 µl each of proteinase K (20 mg ml^−1^) and RNase A (20 mg ml^−1^), denatured at 95 °C for 2 min and supplemented with 6×TriTrack loading dye, and 15 ng of substrate was analysed by Urea–PAGE, as for ABE.

### Differential scanning fluorimetry

ABE and PE proteins were complexed with 1.5-fold molar excess of guide RNA in phosphate-buffered saline (PBS) at room temperature for 15 min to obtain 10 µM RNP. ABE RNP contained an additional 10% (w/v) sucrose. Subsequently, the RNPs were diluted with PBS to 1 µM, SYPRO Orange probe was added to a final concentration of 5×, the samples were aliquoted (10 µl per well) into a 384-well plate (Applied Biosystems, 4483319) and the plate was sealed with optical foil (Applied Biosystems, 4360954). After an additional 15 min incubation, the plate was centrifuged at 1,000 *g* for 1 min at room temperature and installed in a pre-equilibrated Bio-Rad CFX384 thermocycler. Fluorescence was measured in Förster resonance energy transfer (FRET) mode every 0.2 °C from 20 °C to 95 °C. The rate of change of fluorescence (−dF/dT) was used to estimate the melting temperature (*T*_m_). All samples were run in triplicate and plots of averaged data are reported.

### Mammalian cell culture

HEK293-loxP-GFP-RFP cells (referred to as ‘CS cell line’; GenTarget, SC018-Bsd), NIH/3T3 *rd12* cells^41^ and *rd12* reporter cells were maintained in DMEM/F12 medium with GlutaMAX supplement (Thermo Fisher, 10565018) or in DMEM with glutamine (Thermo Fisher, 11965092), both supplemented with 10% FBS (Genesee Scientific, 25-514H) and optional 100 U ml^−1^ penicillin-streptomycin (Thermo Fisher, 15140122) (complete medium) in a humidified incubator at 37 °C and 5% CO_2_.

Primary fibroblasts were isolated from the skin of P0-P3 ROSA mT/mG mice. The mice were euthanized, the skin separated and washed with PBS containing 100 U ml^−1^ penicillin-streptomycin and 40 µg ml^−1^ gentamicin (Thermo Fisher, 15710072). The skins were digested in a 100-mm cell culture dish using a 1:1 mixture of 0.25% trypsin without EDTA (Thermo Fisher, 15050065) and 5 U ml^−1^ dispase (STEMCELL Technologies, 07913) for 1 h at 37 °C, after which the dermis was collected to a new dish and digested with 0.25% collagenase I (Thermo Fisher, 17018029) in serum-free DMEM/F12 for 1 h at 37 °C. The tissue fragments were thoroughly resuspended, filtered through a 70-µm strainer and extensively washed with DMEM/F12 with 15% FBS and 100 U ml^−1^ penicillin-streptomycin by two centrifugation steps (180 *g*, 5 min, room temperature). The cells were maintained in DMEM/F12 with 15% FBS and passaged every 3–4 days using 0.05% trypsin-EDTA (Thermo Fisher, 25300054). After the first passage, the cells were filtered through a 40-µm strainer to remove undigested aggregates that permeated through the 70-µm strainer. Both freshly isolated and cryopreserved cells that were passaged at least twice were used for the experiments. For Cre delivery, medium with 10% FBS was used and the procedure was the same as for the CS cell line. For protein delivery experiments, the cells were seeded 24 h before the experiment in 24-, 48- and 96-well plates to reach 50–70% confluency (~100,000, 50,000 and 25,000 cells per well, respectively, for CS cell line and NIH/3T3 rd12 cells, and 50,000, 24,000 and 10,000 cells per well, respectively, for *rd12* reporter cells and primary fibroblasts).

### Delivery of Cre recombinase

The CS cells and primary ROSA mT/mG fibroblasts were plated on 48-well treated tissue-culture plates in complete medium with 10% FBS. Cre recombinase, fused separately with each of the cell-penetrating peptides (CPPs: CPP5 (KLPVM)^47^, TAT (RKKRRQRRR)^91^ or ANTP (RQIKIWFQNRRMKWKK))^92^, was prepared at various final concentrations (0.10, 0.25, 0.50, 0.75 or 1.00 µM; 4.2, 10.5, 21.0, 31.4 or 41.9 µg ml^−1^, respectively) in 250 µl each of OptiMEM medium (Thermo Fisher, 31985070). Only the 0.5 µM Cre proteins were tested in the primary fibroblasts. For non-covalent complexation with 6×His-CM18-PTD4 peptide (Genscript)^49^, an aliquot of the non-covalent peptide was added to Cre recombinase (free or fused with one of the CPPs) and incubated for 15 min at room temperature. The reporter cells were washed with 180 µl of OptiMEM medium, which was then exchanged with OptiMEM plus one of the various preparations of Cre recombinase. After 3 h, the medium was exchanged with complete medium. The CS cells and ROSA mT/mG fibroblast cells were maintained post treatment for a total of 24 h and 72 h, respectively. For imaging, the medium was exchanged to FluoroBrite DMEM (Thermo Fisher, A1896701) and the cells were imaged using a Keyence BZ-X810 microscope with GFP and Texas Red optical filters.

### Flow cytometry

Cells were washed with PBS (Thermo Fisher, 10010023), detached with 0.05% trypsin (Thermo Fisher, 25300054), transferred to a 96-well round-bottom plate and centrifuged at 180 *g* for 5 min at room temperature. Centrifuged cells were washed with a FACS buffer (PBS with 2% FBS, 100 U ml^−1^ penicillin-streptomycin), centrifuged again and resuspended in the FACS buffer with 1 µg ml^−1^ 4′,6-diamidino-2-phenylindole (DAPI; Thermo Fisher, 62248). The cells were analysed using a Novocyte Quanteon (Agilent) flow cytometer with Pacific Blue (445/45 nm), FITC (530/30 nm) and PE (586/20 nm) optical filters. Cells were gated on forward and side scatter, viability via DAPI exclusion and single cells (Supplementary Fig. 9).

### Subretinal injections

Cre proteins with or without fused cell-penetrating peptides were diluted in OptiMEM to 10 µM. 6×His-CM18-PTD4 peptide was optionally added to Cre in 10-fold excess and incubated for at least 15 min before injection. A lipoplex of Cre with Lipofectamine 3000 was injected at 10 µM, with 2% Lipofectamine 3000 by volume (419 ng Cre per eye, 0.02 µl Lipofectamine 3000 per eye). AAV2/1-Cre (Addgene, 105537-AAV1; 1.8 × 10^10^ vg) or vesicular stomatitis virus G glycoprotein (VSV-G) pseudotyped eVLP-Cre (concentrated by ultracentrifugation 1,000-fold as previously described^28^) were injected as a positive control for ROSA mT/mG mice. Mice were anaesthetized by intraperitoneal injection of a cocktail consisting of 20 mg ml^−1^ ketamine and 1.60 mg ml^−1^ xylazine in PBS at a dose of 100 mg kg^−1^ of ketamine and 8 mg kg^−1^ of xylazine, and their pupils were dilated by topical administration of 1% tropicamide ophthalmic solution (Akorn, 17478-102-12) and 10% phenylephrine ophthalmic solution (MWI Animal Health, 054243). The corneas were hydrated with GenTeal Severe Lubricant Eye Gel (0.3% hypromellose, Alcon). Subretinal injections were performed using an ophthalmic surgical microscope (Zeiss). An incision was made through the cornea adjacent to the limbus at the nasal side using a 27-gauge needle. A 34-gauge blunt-end needle (World Precision Instruments, NF34BL-2) connected to an RPE-KIT (World Precision Instruments, RPE-KIT) with SilFlex tubing (World Precision Instruments, SILFLEX-2) was inserted through the corneal incision while avoiding the lens and advanced into the subretinal space. Each mouse received a 1-μl injection per eye, and volume and rate were controlled with a UMP3 UltraMicroPump (World Precision Instruments, UMP3-4). After surgery, the mice were placed on a heating pad and anaesthesia was reversed with intraperitoneal atipamezole (2.5 mg kg^−1^; MWI Animal Health, 032800). Triple antibiotic ophthalmic ointment (neomycin, polymyxin and bacitracin) was administered to the cornea to promote recovery.

### Two-photon imaging of mouse eyes

After killing, intact enucleated mouse eyes were submerged in room temperature PBS. Pulsing infrared light from a Ti:sapphire laser (Coherent, Vision S; tunable between 690 and 1,050 nm) was set to 950 nm and attenuated in a controlled, variable manner with an electro-optic modulator. To image and spectrally separate GFP and tdTomato, two internal spectral detectors were used with their detection bandwidths set to 490–545 nm for GFP and 590–680 nm for tdTomato. A 1.0 NA ×20 Leica objective was used for the imaging^93,94^.

### Generation of the *rd12* reporter cell line

The *rd12* reporter construct was synthesized by GenScript according to the following strategy: 198 bp of the mouse *Rpe65* cDNA was flanked by 5’-mCherry and 3’-eGFP, and the whole construct was inserted into the pcDNA3.1/(Zeo)+ backbone with BamHI and XhoI restriction sites (mCherry-*rd12*-eGFP). The mCherry-*rd12*-eGFP construct was then subcloned into pMXs-IRES-blasticidin via double digestion of the backbone with BamHI and XhoI. The downstream sequence of the internal ribosomal entry site (IRES) and blasticidin-resistance gene enabled co-expression of the reporter and selectable marker. The *rd12* reporter cell line was generated by transduction of NIH/3T3 cells with retrovirus obtained from Phoenix-Eco cells transfected with pMXs-mCherry-*rd12*-eGFP-IRES-blasticidin^95,96^ according to a previously published protocol^97^. Transduced cells were then selected with blasticidin for 10 days (5 μg ml^−1^; Thermo Scientific, R25001). The surviving cells were sorted using flow cytometry to select high-expressing mCherry-positive clones and then seeded into 96-well plates for clonal selection. Single colonies were screened for proper expression and editability via Gene Juice (MilliporeSigma, 70967-3) co-transfection of pCMV-NG-SpCas9-ABE7.10max and pSPgRNA-*rd12*-A6, and via proper co-expression of mCherry and eGFP. Finally, the clones were sequenced. No additional characterization was performed on the sorted cells.

### Delivery of ABE and PE in vitro

ABE RNP was assembled by incubation of up to 20 µM ABE for at least 15 min at room temperature with 1.1-fold excess of sgRNA in OptiMEM medium supplemented with 10% (w/v) sucrose. The sgRNA used for experiments in cell lines and in vivo contained additional modifications as described (Supplementary Information, Supplementary Sequences, rd12-A6-sgRNA highly modified) and was supplied by IDT^75^. The 6×His-CM18-PTD4 peptide was dissolved in 100 mM HEPES pH 8.0 to achieve a peptide concentration of 10 mM; the final pH of this stock solution was ~7.0. The 6×His-CM18-PTD4 peptide was diluted to 400 µM with water, added to ABE RNP and incubated for an additional 15 min. CPP fusions of ABE with or without 6×His-CM18-PTD4 peptide were diluted to their final concentrations in OptiMEM containing 2% or 10% sucrose, and 100 µl of each of the mixtures was applied on the cells in a 48-well plate that had been washed with OptiMEM medium. After 3 h, the medium was exchanged with 250 µl of complete medium.

To assemble Lipofectamine 3000 lipoplexes, 0.5 µl of Lipofectamine 3000 per well of a 48-well plate or 0.2 µl per well of a 96-well plate was diluted with OptiMEM containing 10% (w/v) sucrose to 12.5 and 5 µl per well, respectively; the ABE RNPs diluted with OptiMEM with 10% (w/v) sucrose to 12.5 µl (48-well) or 5 µl (96-well) were then added to the diluted lipids and incubated for 15 min at room temperature. A lipoplex of ABE with Lipofectamine 3000 in a volume of 25 µl was added to the cells with 225 µl fresh complete medium in a 48-well plate, or 10 µl was added to the cells with 90 µl medium in a 96-well plate. The cells were incubated for 48 h before analysis. Plasmid DNA transfections were done in 48-well plates using 160 ng pCMV-NG-SpCas9-ABE, 80 ng pSPgRNA-*rd12*-A6 and 0.75 µl Lipofectamine 3000 per well, following manufacturer protocol. PE was delivered in vitro, similarly as ABE, but without sucrose, unless otherwise noted. Activities of ABE and PE in the *rd12* reporter cell line were analysed by fluorescence microscopy and flow cytometry, as described above for Cre delivery.

Rescue of RPE65 expression was analysed using NIH/3T3 cells stably expressing *Rpe65 rd12* cDNA, as previously described^41^. The ABE and PE were applied on these cells, and after 48 h, the cells were detached using trypsin and washed three times with PBS. One-tenth of the cell suspension was lysed with 10 mM Tris pH 7.5 and 0.05% SDS with 0.02 mg ml^−1^ proteinase K for 1 h at 37 °C, then the proteinase K was inactivated at 85 °C for 45 min. The lysate was used as a template for PCR and the products were subjected to next-generation sequencing. The remaining cells were lysed in 1× RIPA buffer (Cell Signaling Technology, 9806) with 1× cOmplete Ultra EDTA-free protease inhibitors (Roche) for 1 h on a rotator in a cold room and then centrifuged at 17,000–21,400 *g* for 20 min at 4 °C, and the supernatant was used for analysis. Protein concentration in the extracts was measured using the BCA assay (Thermo Fisher, 23252) and the extracts were subjected to western blotting.

### Next-generation sequencing

Complementary DNA was synthesized from RNA with the High Capacity RNA-to-cDNA kit (Thermo Fisher, 4387406) according to manufacturer instructions. Of the isolated genomic DNA or cDNA, 0.5–1 μl was used as input for the first of two PCR reactions (PCR1). Genomic loci were amplified in PCR1 using Phusion Plus polymerase (Thermo Fisher, F631S). PCR1 primers for genomic loci are listed in Supplementary Table 2 (marked as HTS_fwd and HTS_rev). PCR1 was performed as follows: 98 °C for 30 s; 30 cycles at 98 °C for 10 s, 60 °C for 20 s and 72 °C for 30 s; 72 °C for 5 min. PCR1 products were confirmed on a 2% agarose gel. One microlitre of PCR1 was used as input for PCR2 to install Illumina barcodes. PCR2 was conducted for 9 cycles of amplification using a Phusion HS II kit (Life Technologies). Following PCR2, samples were pooled and gel purified in a 1% agarose gel using a Qiaquick Gel Extraction kit (Qiagen). Library concentration was quantified using the Qubit High-Sensitivity Assay kit (Thermo Fisher). Samples were sequenced on an Illumina MiSeq instrument (paired-end reads, read 1: 200–280 cycles, read 2: 0 cycles) using an Illumina MiSeq 300 v.2 kit (Illumina).

### High-throughput sequencing data analysis

Sequencing reads were demultiplexed using the MiSeq Reporter software (Illumina) and analysed using CRISPResso2 as previously described^27,98^. Batch analysis mode (one batch for each unique amplicon and sgRNA combination analysed) was used in all cases. Reads were filtered according to minimum average quality score (*Q* > 30) before analysis. The following quantification window parameters were used: -w 20 -wc -10. Base editing efficiencies are reported as the percentage of sequencing reads containing a given base conversion at a specific position. Prism 10 (GraphPad) was used to generate dot plots and bar plots.

### Western blotting

Protein was analysed via western blotting and detected using mouse anti-RPE65 antibody (produced in house)^99^ or mouse anti-SpCas9 (clone 7A9, Biolegend, 844302) diluted 1:1,000 in 5% non-fat dry milk in PBS (Bio-Rad, 1610780) with 0.1% Tween 20 (MilliporeSigma, P9416) (PBST). The lysates (7–10 µg total protein) were separated by SDS–PAGE, transferred to a polyvinylidene fluoride membrane (MilliporeSigma, IPVH00010), blocked for 1 h with 5% non-fat dry milk in PBST at room temperature and incubated with the primary antibodies overnight in the cold room. After washing 4 times with PBST for 5 min each, the blots were incubated for 1 h at room temperature with a horseradish peroxidase (HRP)-linked horse anti-mouse antibody (Vector Laboratories, PI-2000-1) diluted 1:2,500 in 5% non-fat dry milk in PBST. The signals were detected with SuperSignal West Pico Plus Chemiluminescent substrate (Thermo Fisher, 34577). Next, the antibodies were stripped from the membrane with 0.2 M glycine pH 2.2, 0.1% SDS and 1% Tween 20; then, the membranes were washed, blocked and re-probed for 1 h at room temperature using rabbit anti-β-actin polyclonal antibody (1:2,000; Cell Signaling Technology, 4970S). Goat anti-rabbit IgG with HRP (1:2,500; Cell Signaling Technology, 7074S) was used as the secondary antibody before developing the blots, as described above.

### Delivery of ABE in vivo

ABE was diluted into OptiMEM containing additional sucrose (10 or 25% (w/v) final, as indicated); or into the ABE storage buffer containing high salt concentration (390 mM NaCl final) without added sucrose. To form RNPs, ABE was added to guide RNA dissolved in water and incubated for 15 min at room temperature. For combination with 5-fold molar excess of 6×His-CM18-PTD4 peptide, ABE RNPs were added to a peptide stock solution diluted to 1 mM. In the case of Lipofectamine 3000, ABE RNPs were added to the undiluted reagent. RNPs were incubated with the reagents for at least 15 min at room temperature before subretinal injection into *rd12* mice as described.

### RPE dissociation, genomic DNA and RNA, and lysate preparation

Mouse eyes were dissected under a light microscope to separate the posterior eyecup (containing RPE, choroid and sclera) from the retina and anterior segment. Each posterior eyecup was immediately immersed in RLT Plus (Qiagen). RPE, choroid and scleral cells were detached from the posterior eyecup by gentle pipetting, followed by removal of the remaining posterior eyecup. Cells were then processed for genomic DNA and RNA using the AllPrep DNA/RNA Micro kit according to manufacturer instructions (Qiagen, 80284). To prepare the protein lysate from the mouse RPE tissue, the dissected mouse posterior eyecup was transferred to a microcentrifuge tube containing 40 μl of ice-cold RIPA buffer with protease inhibitors and homogenized with a motorized tissue grinder (Fisher Scientific, K749540-0000), incubated on ice for 20 min and then centrifuged for 20 min at 21,000 *g* at 4 °C. The resulting supernatant was pre-cleared with Dynabeads Protein G (Thermo Fisher, 10003D) by rotation at 4 °C for 15 min to remove immunoglobulin contaminants from blood before loading on the gel.

### Immunohistochemistry of RPE flatmounts and cryosections

Mouse eyes were enucleated and fixed with 4% paraformaldehyde in PBS for 20 min at room temperature and washed three times in PBS for 5 min each. To make RPE flatmounts, the anterior segment and retina were removed from the posterior eyecup under a dissecting microscope, and four radial cuts were made towards the optic nerve to flatten the eyecup into an RPE flatmount. Samples were permeabilized and blocked in 0.1% Triton X-100 (Sigma-Aldrich, T8532) with 3% normal goat serum (NGS) in PBS for 30 min and incubated with the following primary antibodies in PBS, 0.1% Triton X-100 and 3% NGS: mouse anti-RPE65 antibody (1:100; in house) and rabbit anti-ZO-1 polyclonal antibody (1:100; Invitrogen, 61-7300) overnight at 4 °C. The next day, samples were washed three times in PBS for 5 min each and then incubated with the appropriate secondary antibodies in PBS, 0.1% Triton X-100 and 3% NGS, including Alexa Fluor 555-conjugated goat anti-mouse IgG (1:200; Thermo Fisher, A11032) and Alexa Fluor 647-conjugated goat anti-rabbit IgG (1:200; Thermo Fisher), for 2 h at room temperature in the dark. The secondary antibodies were then removed and the flatmounts were incubated in DAPI (1 µg ml^−1^; Thermo Fisher, 62248) in PBS for 10 min. Samples were washed three times in PBS for 5 min each. The samples were then mounted with VECTASHIELD HardSet Antifade Mounting Medium (Vector Labs, H-1400-10) and imaged on a Keyence BZ-X810 All-in-One fluorescence microscope.

### Electroretinography

Before ERG recording, mice were dark adapted for 1 week. Under a safety light, mice were anaesthetized by isoflurane inhalation, and their pupils were dilated with topical administration of 1% tropicamide ophthalmic solution (Akorn, 17478-102-12) and 10% phenylephrine ophthalmic solution (MWI Animal Health, 054243), followed by hypromellose (Akorn, 9050-1) for hydration. The mouse was placed on a heated Diagnosys Celeris rodent-ERG device (Diagnosys). Ocular stimulator electrodes were placed on the corneas, the reference electrode was positioned subdermally between the ears, and a ground electrode was placed in the rear leg. The eyes were stimulated with a green-light flash stimulus (peak emission 544 nm, bandwidth ∼160 nm) of −0.3 log (cd s m^−2^) light intensity. The responses for 10 stimuli with an inter-stimulus interval of 10 s were averaged, and the a- and b-wave amplitudes were acquired from the averaged ERG waveform. Data were analysed with the Espion V6 software (Diagnosys). For the RNP LNP optimization study, eyes that received LNP and had no ERG response after treatment were excluded from the analyses.

### Pupillary light reflex (PLR)

The PLR was characterized in mice (*n* = 5 for each group) using the A2000 computerized pupillometer (Neuroptics). Mice were dark adapted for 6 h before recordings in a dark room. This pupilometer system consists of a sensing device equipped with two infrared cameras that independently record and track dynamics of each pupil. The light profile consisted of four white-light stimuli (1.2 log, 10^1.2^ W m^−2^), each for 500 ms. For the duration of the PLR testing routine, mice were kept under isoflurane anaesthesia. The experiments were carried out under scotopic conditions, with no background illumination from the pupillometer, with the infrared cameras as the primary light source. The maximum size of the pupil after dark adaptation was quantified at 2 min after anaesthesia and was used to establish baseline size. The pupil response was expressed as percent constriction of the pupil when compared to baseline. Captured digital movies of pupil responses were recorded using the Active Presenter software (v.9.1.3, Atomi Systems), and the videos were subsequently decomposed into individual frames using the Adobe Premiere Rush programme (v.10.0.1, Adobe Systems) for manual verification of pupil dynamics and calculation of absolute pupil diameters from the recorded images.

### Retinoid analysis

Mice were dark adapted for 2 days before eye enucleation. Eyes were homogenized in 1 ml of a 10 mM sodium phosphate buffer (pH 8.0) containing 50% (v/v) methanol (Sigma-Aldrich, 34860-1L-R) and 100 mM hydroxylamine (pH 8.0) (Sigma-Aldrich, 159417-100G). After 15 min incubation at room temperature, 2 ml of 3 M NaCl was added. The resulting sample was extracted twice with 3 ml ethyl acetate (Fisher Scientific, E195-4). Then, the combined organic phase was dried in vacuo and reconstituted in 250 μl hexane. Extracted retinoids (100 µl) were separated on a normal-phase HPLC column (Zorbax Sil, 5 µm, 4.6 mm × 250 mm; Agilent Technologies) connected to an Agilent Infinity 1260 HPLC system equipped with a diode-array detector. Separation was achieved with a mobile phase of 0.6% ethyl acetate in hexane (Fisher Scientific, H302-4) at a flow rate of 1.4 ml min^−1^ for 17 min, followed by a step increase to 10% ethyl acetate in hexane for an additional 25 min. Retinoids were detected by monitoring absorbances at 325 nm and 360 nm using Agilent ChemStation software.

### Encapsulation of ABE and PE LNPs

RNPs were assembled by mixing 10 μM purified ABE or PE with synthetic sgRNA (ABE) or epegRNA (PE) at a 1:1.1 molar ratio. The buffer for ABE was 10 mM HEPES (pH 7.0), 500 mM NaCl, 20% (v/v) glycerol; for PE, the buffer was 10 mM HEPES (pH 7.0), 500 mM NaCl, 5% (v/v) glycerol; and guide RNA was dissolved in water. Proteins were diluted, supplemented with 10% (w/v) sucrose from 50% (w/v) stock in water and added to guide RNA. Final composition of the buffer in which RNPs were assembled was 2.8 mM HEPES (pH 7.0), 140 mM NaCl, 10% (w/v) sucrose, 5.6% (v/v) glycerol (ABE); or 1.4% (v/v) glycerol (PE). The RNPs were incubated at room temperature for at least 15 min. Transient turbidity, which cleared during incubation, was observed. Immediately before encapsulation, RNPs were diluted to 0.711 μM in a 50 mM Tris-acetate buffer (pH 6.0) with 10% (w/v) sucrose to achieve a final NaCl concentration of 10 mM. The lipids used to encapsulate pre-formed RNPs comprised an ionizable cationic lipid (either CL4H6 (Cayman Chemical, 37279), SM102 (Broadpharm, BP-25499) or DODMA (Avanti Polar Lipids, 890899), all of which have p*K*_a_s > 6.0 at 6.25, 6.68 and 6.59, respectively) and co-lipids DSPC (Avanti Polar Lipids, 850365), cholesterol (Avanti Polar Lipids, 700100) and DMG-PEG 2000 (Avanti Polar Lipids, 880151) (Supplementary Fig. 5) at a molar composition of 50/10/38.5/1.5, respectively, to encapsulate pre-formed RNPs. For optimization of DMG-PEG 2000 lipid content from 1.5 to 10 mole%, the concentration of cholesterol was decreased accordingly. In some formulations, 2.5 mole% of SOPS (18:0–18:1 PS, Avanti Polar Lipids, 840039C) was used with 7.5 mole% of DSPC. In brief, the lipids were dissolved in ethanol and rapidly combined with pre-formed RNP at a volume ratio of 1:3 (ethanol:aqueous) and a total lipid:guide RNA weight ratio of 40:1 (approximate total lipid:protein weight ratio of 7.75:1). The combination was performed by microfluidic mixing using a Precision NanoSystems Ignite device (Precision NanoSystems). Immediately after mixing, the formed LNPs were dialysed two times for 2 h each at room temperature against 20 mM Tris, 4.3 mM Na acetate (pH 7.4) and 10% (w/v) sucrose (TAS buffer) to remove the ethanol and deprotonate the ionizable cationic lipid at neutral pH. Final concentration of protein was ~0.53 µM (0.10 mg ml^−1^ ABE8e, 0.13 mg ml^−1^ PE), and that of guide RNA was 0.59 µM (0.02 mg ml^−1^ sgRNA, 0.03 mg ml^−1^ epegRNA). The LNPs were transferred to ice and concentrated to no less than one-fifth of the initial volume using an Amicon Ultra centrifugal filter with molecular weight cut-off of 30 kDa (Merck, UFC903024); the concentrated LNPs were distributed into aliquots, quickly frozen on a pre-cooled metal block and stored at −80 °C. The percent recovery and concentration factor of the LNPs were estimated using SDS–PAGE electrophoresis and CBB staining. Fluorescence intensity of Coomassie dye bound to protein was measured using ChemiDoc MP imager and analysed using ImageLab software v.6.1.0 (Bio-Rad). Doses of RNP LNP are reported in vitro as a final concentration of RNP and in vivo as RNP concentration and injected volume.

### Particle-size distribution analysis

Particle-size distribution was measured using a Malvern Zetasizer Advance Nano (Malvern Panalytical). Twenty microlitres of freshly dialysed LNP were diluted to 200 μl with TAS buffer and subjected to particle-size distribution measurement in triplicates. The particle size distributions were processed by the accompanying software to calculate average particle diameter and polydispersity index (PdI).

### Cryoelectron-microscopic imaging of LNPs

LNP samples were concentrated 3–4 times in an Amicon Ultra 0.5 device with a 10 kDa molecular weight cut-off. LNP solution (2.5 μl) was applied onto a Quantifoil 200 mesh grid coated with a thin carbon film (Ted Pella). Grids were blotted for 2 s with filter paper, then plunged into liquid ethane using a manual plunger. The image was collected on an FEI Tecnai TF20 high resolution transmission electron microscope equipped with a K2 Direct-Detection Camera at an accelerating voltage of 200 kV.

### ABE encapsulation immunoassay

LNPs were diluted 1:3 in an immunoprecipitation buffer (10 mM HEPES (pH 7.0), 150 mM NaCl, 10% (w/v) sucrose), and 80 μl of diluted LNPs were incubated with 20 μl of 1D4 resin for 30 min in a cold room in an overhead mixer. As a control, pre-formed free RNP was diluted in the TAS buffer to the approximate concentration of RNP in a prepared LNP and also incubated with 20 μl of 1D4 resin. Samples were then centrifuged at 700 *g* for 5 min at 4 °C. The supernatant was filtered with centrifugation at 200 *g* for 2 min through a 30-µm polyethylene filter. Pelleted resin was resuspended in 500 μl of the immunoprecipitation buffer and washed by centrifugation at 700 *g* for 5 min. The supernatant was discarded and the washing process was repeated four separate times to ensure thorough washing. To elute the RNP, 80 μl of the elution buffer (1 mg ml^−1^ of 1D4 peptide in the immunoprecipitation buffer) was introduced to the resin and the samples were incubated overnight in a cold room in an overhead mixer. After incubation, the samples were centrifuged and filtered as described above. Filtered samples were then analysed by western blot, as described.

### Size exclusion chromatography of ABE LNP

The LNPs containing ABE8e RNP or free ABE8e RNP (112 µg RNP in both) were diluted into 500 µl of 1× PBS (Corning, 46-013-CM) with 0.001% Pluronic F-68 (Gibco, 24040032), filtered on a pre-washed 0.22-µm cellulose acetate centrifugal filter (Corning, 8160) and resolved on a HiPrep 16/60 Sephacryl S-500 HR column (Cytiva, 28935606) at a flow rate of 0.4 ml min^−1^. One millilitre fractions were collected and analysed by western blot with anti-Cas9 antibodies as described.

### Quantification of ABE and PE by mass spectrometry

Deionized water in all experiments was generated using a Milli-Q water-purification system (Millipore). Formic acid (FA), ammonium bicarbonate and acetonitrile of MS grade were purchased from Fisher chemical. Iodoacetic acid and DTT were of analytical grade and supplied by Millipore. Sequencing-grade modified trypsin was provided by Promega. Stable-isotope-labelled peptides (SIL peptides) were synthesized with alkylated cysteines by GenScript. The stock solutions of all the peptides were prepared by accurately weighing the synthetic peptides and then dissolving them in water or dimethylsulfoxide following manufacturer instructions. The SIL peptides were diluted in water before adding to the samples (Supplementary Table 3).

The samples were diluted with 50 mM ammonium bicarbonate and reduced with 10 mM DTT for 1 h at 56 °C and alkylated with 20 mM iodoacetic acid for 30 min at room temperature in the dark. Then, the SIL peptides were spiked into protein samples, and then free trypsin was added at a trypsin to protein ratio of 1:50 and incubated overnight at 37 °C. Trypsin activity was inhibited by acidification with 0.1% FA and the samples were then desalted using a C18 desalting column (Nest). After drying completely by speed vacuum, peptides were dissolved in 0.1% FA. The samples were analysed by LC–MS/MS using a Vanquish LC instrument (Thermo Fisher) coupled in-line with a Q Exactive mass spectrometer (Thermo Fisher) with an ESI source. Mobile phase A was composed of 0.1% FA in water and mobile phase B was composed of 0.1% FA in acetonitrile. The total flow rate was 0.4 ml min^−1^. Peptides were separated with a 25-min gradient on an Acquity UPLC BEH C18 column (1.7 μm, 2.1 mm × 100 mm; Waters). The acquisition method combined a full scan method with a time-scheduled sequential parallel-analysis monitoring (PRM) method. For PRM, MS2 scan parameters were set to select the *m*/*z* ratio of the natural peptides of Cas9, TadA deaminase and reverse transcriptase, and their corresponding SIL peptides with defined elution time windows. MS1 scans were acquired at the *m*/*z* range of 300–1,000, mass resolution of 70,000, automatic gain control (AGC) target of 1 × 10^6^ and maximum ion injection time of 50 ms. The PRM scans were acquired at a resolution of 17,500, AGC target value of 1 × 10^5^, maximum ion injection time of 50 ms and isolation window of 2.0 *m*/*z*.

### Local field potential and single unit recordings, visual stimulation and data analysis

Mice were initially anaesthetized with 2% isoflurane in a mixture of N_2_O/O_2_ (70%/30%) and then placed into a stereotaxic apparatus. A small, custom-made plastic chamber was glued (Vetbond) to the exposed skull. After 1 day of recovery, re-anaesthetized animals were placed in a custom-made hammock, maintained under isoflurane anaesthesia (1–2% in O_2_), and multiple single tungsten electrodes were inserted into a small craniotomy above the V1 and SC. Once the electrodes were inserted, the chamber was filled with sterile agar and sealed with sterile bone wax. During recording sessions, animals were kept under isoflurane anaesthesia (0.5–1% in 30% O_2_). EEG and EKG scans were monitored throughout the experiments and body temperature was maintained with a heating pad (Harvard Apparatus).

Data were acquired using a 32-channel Scout recording system (Ripple). The local field potential (LFP) from multiple locations was bandpass filtered from 0.1 Hz to 250 Hz and stored with spiking data on a computer with a 1-kHz sampling rate. The LFP signal was cut according to stimulus time stamps and averaged across trials for each recording location to calculate visually evoked potentials (VEP)^41,100–102^. The evoked potential across all layers was recorded and the most robust response was used for comparisons between groups at the same SC or V1 layer.

The spike signal was bandpass filtered from 500 Hz to 7 kHz and stored in a computer hard drive at a 30 kHz sampling frequency. Spikes were sorted online in Trellis software (Ripple) while performing visual stimulation. Visual stimuli were generated in Matlab (Mathworks) using the Psychophysics Toolbox^103–105^ and displayed on a gamma-corrected LCD monitor (55 inches, 60 Hz; 1,920 × 1,080 pixels; 52 cd m^−2^ mean luminance). Stimulus onset times were corrected for LCD-monitor delay using a photodiode and microcontroller (in-house design).

Vision was assessed using protocols published in our previous work^41,101,106,107^. Cells were first tested with 100 repetitions of a 500-ms bright flash of light (105 cd m^−2^) for the presence of the visually evoked responses. When cells showed signs of robust visually driven activity, we used further drifting grating stimuli to assess the properties of the spatiotemporal receptive fields. Briefly, each cell was evaluated for orientation selectivity, optimal stimulus size, optimal spatial frequency, optimal temporal frequency and contrast sensitivity. Recorded tuning curves were further normalized between 0 and 1 for visual purposes and plotted together for comparison. Data are presented as mean ± s.e.m. The level of statistical significance was set at *P* < 0.05 for two-tailed Mann–Whitney *U*-tests. The figures show single recording locations from the SC and V1 recordings as examples. Offline data analysis and statistics were performed in Matlab (Mathworks).

Tuning curves were calculated on the basis of the average spike rate. Optimal visual parameters were chosen as the maximum response value. Orientation tuning was measured in degrees at the half-width at half-height (HWHH; 1.18 × σ) on the basis of fits to Gaussian distributions using equation (1):

1 $${R}_{{O}_{\rm{s}}}={\rm{baseline}}+{R}_{\rm{p}}{e}^{\frac{-{\left({O}_{\rm{s}}-{O}_{\rm{p}}\right)}^{2}}{{2\sigma }^{\,2}}}+{R}_{\rm{n}}{e}^{\frac{-{\left({O}_{\rm{s}}-{O}_{\rm{p}}+180\right)}^{2}}{{2\sigma }^{\,2}}}$$

where *O*_s_ is the stimulus orientation, *R*_*O*s_ is the response to different orientations, *O*_p_ is the preferred orientation, *R*_p_ and *R*_n_ are the responses at the preferred and non-preferred direction, *σ* is the tuning width and ‘baseline’ is the offset of the Gaussian distribution. Gaussian fits were estimated without subtracting spontaneous activity^107^.

The optimal spatial and temporal frequency was extracted from the data fitted to Gaussian distributions using equation (2)^107^:

2 $${R}_{\frac{{\rm{SF}}}{{\rm{TF}}}}={\rm{baseline}}+{R}_{{\rm{pref}}}{e}^{\frac{-{\left(\frac{{\rm{SF}}}{{\rm{TF}}}-{\frac{{\rm{SF}}}{{\rm{TF}}}}_{{\rm{pref}}}\right)}^{2}}{{2\sigma }^{\,2}}}$$

where *R*_SF/TF_ is the estimated response and *R*_pref_ indicates response at a preferred spatial or temporal frequency. SF/TF indicates spatial or temporal frequency, *σ* is the standard deviation of the Gaussian and the baseline is the Gaussian offset.

### Statistical analyses

Unless otherwise stated, data are presented as mean ± s.d. and statistical analyses were performed using GraphPad Prism 10.0, with *P < 0.05, **P < 0.01, ***P < 0.001, ****P < 0.0001 and ^NS^*P* ≥ 0.05.

### Reporting summary

Further information on research design is available in the Nature Portfolio Reporting Summary linked to this article.

## Supplementary information

Supplementary Information Supplementary Figures, Tables, Discussion, Sequences and References.

Reporting Summary

Supplementary Data Source data for Supplementary Figs. 1–4 and 6–8.

Supplementary Video 1 Supplementary video for Fig. 2i.

Supplementary Video 2 Supplementary video for Fig. 2j.

Supplementary Video 3 Supplementary video for Fig. 2k.

Supplementary Video 4 Supplementary video for Fig. 2l.

Supplementary Video 5 Supplementary video for Fig. 2m.

Supplementary Video 6 Supplementary video for Fig. 2n.

Supplementary Video 7 Supplementary video for Fig. 2o.

Supplementary Video 8 Supplementary video for Fig. 2p.

Supplementary Video 9 Pupillary reflex of a wild-type C57BL/6 mouse.

Supplementary Video 10 Pupillary reflex of an *rd12* mouse.

Supplementary Video 11 Pupillary reflex of an ABE RNP LNP-treated *rd12* mouse.

## Data Availability

High-throughput sequencing data are available from the National Center for Biotechnology Information Sequence Read Archive database, under accession PRJNA1124167. Source data for the figures are provided with this paper. The raw and analysed datasets generated during the study are available for research purposes from the corresponding authors on reasonable request.

## Soruce data

Source Data for Figs. 1–6 and Extended Data Figs. 1–4 Source data and unprocessed gels and western blots.
