# Supplementary material for: Safer and efficient base editing and prime editing via ribonucleoproteins delivered through optimized lipid-nanoparticle formulations
Source: Nat Biomed Eng. 2024 Nov 28;9(1):57–78. doi: 10.1038/s41551-024-01296-2 (PMC11754100; doi:10.1038/s41551-024-01296-2)
Supplement: Supplementary file 1 — Supplementary Figures, Tables, Discussion, Sequences and References. [file 41551_2024_1296_MOESM1_ESM.pdf]

nature biomedical engineering

Supplementary information https://doi.org/10.1038/s41551-024-01296-2

Safer and efficient base editing and prime editing via ribonucleoproteins delivered through optimized lipid-nanoparticle formulations

In the format provided by the authors and unedited

Contents

Supplementary figures....2
- Supplementary Fig. 1 I Differential-scanning fluorimetry profiles of ABE and PE....2
- Supplementary Fig. 2 I Titration of HEK293-loxP-GFP-RFP cells with CPP-fused Cre....3
- Supplementary Fig. 3 I Titration of HEK293-loxP-GFP-RFP cells with Cre and 6xHis-CM18-PTD4 peptide....4
- Supplementary Fig. 4 I Minus-enzyme controls for HEK293-loxP-GFP-RFP cells with 6xHis-CM18-PTD4 peptide....5
- Supplementary Fig. 5 I Structures of lipids used in the study....6
- Supplementary Fig. 6 I Mass spectrometric quantification of ABE and PE....7
- Supplementary Fig. 7 I Off-target analysis of rd12 mice treated with ABE- and PE RNP LNP....8
- Supplementary Fig. 8 I Non-targeting ABE and PE RNP LNPs do not result in correction of Rpe65 or restore scotopic flash ERG responses....9
- Supplementary Fig. 9 I Flow cytometry gating strategies....10

Supplementary discussion....11
- CPP versus LNP for RNP delivery....11
- Estimation of number of RNPs in an LNP....11
- Comparison of RNP encapsulation approaches....11

Supplementary tables....13
- Supplementary Table 1 I Oligonucleotides used in this work....13
- Supplementary Table 2 I PCR primers used in this work....14
- Supplementary Table 3 I Sequences of selected SIL peptides, purity, concentration of stock solution and spiking solution....15
- Supplementary Table 4 I Sequences of primers used for off-target analyses....16

Supplementary sequences....17
- Peptide sequences....17
- RNA sequences....17
- Plasmid DNA sequences....17

Supplementary references....26

Captions for the supplementary videos....27

Supplementary figures

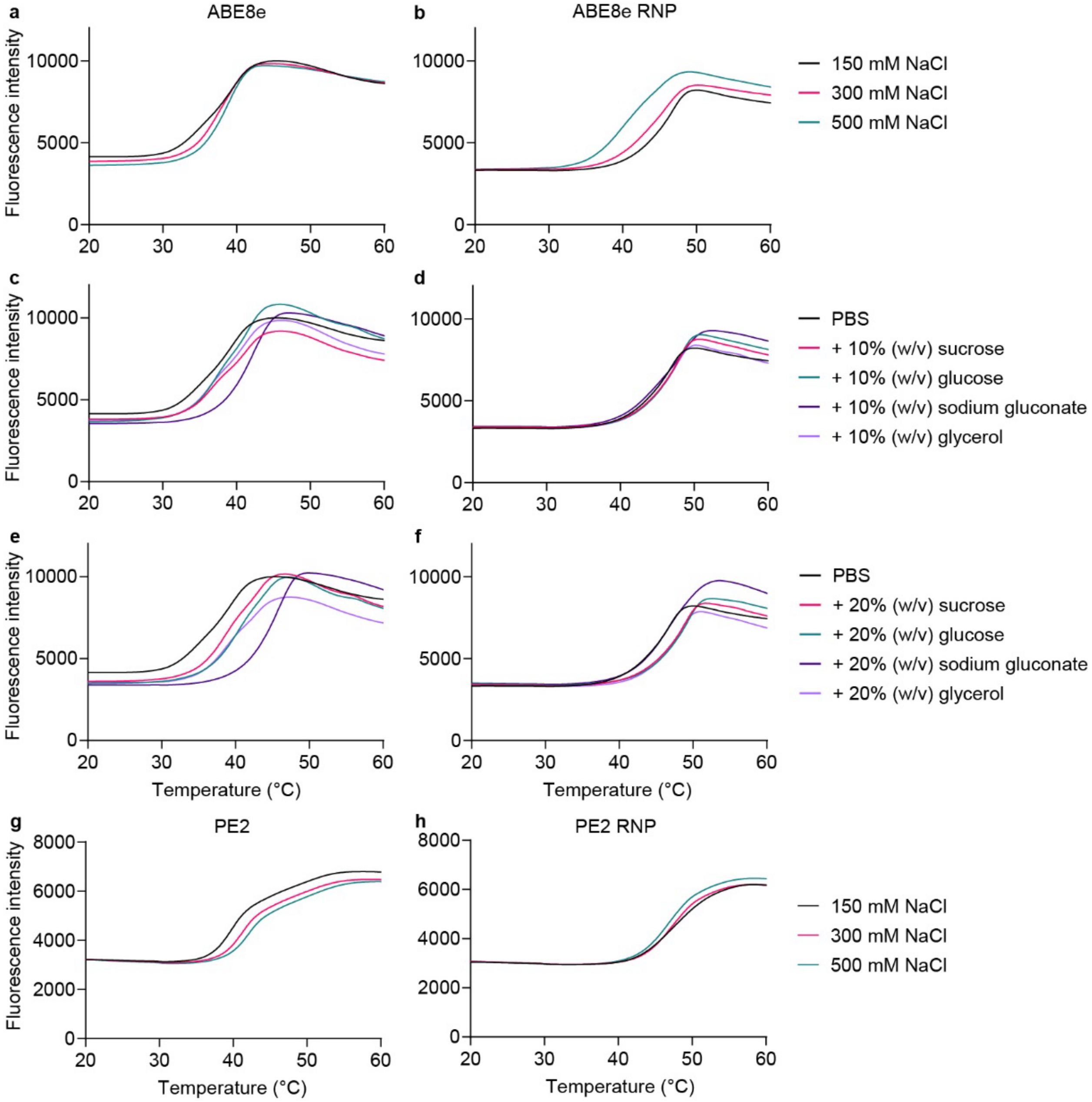

Supplementary Fig. 1 | Differential-scanning fluorimetry profiles of ABE and PE. ABE8e and PE2 proteins, with or without respective guide RNAs, were incubated with increasing concentrations of NaCl, and with optional additives. Plots were averaged from n = 3 replicates.

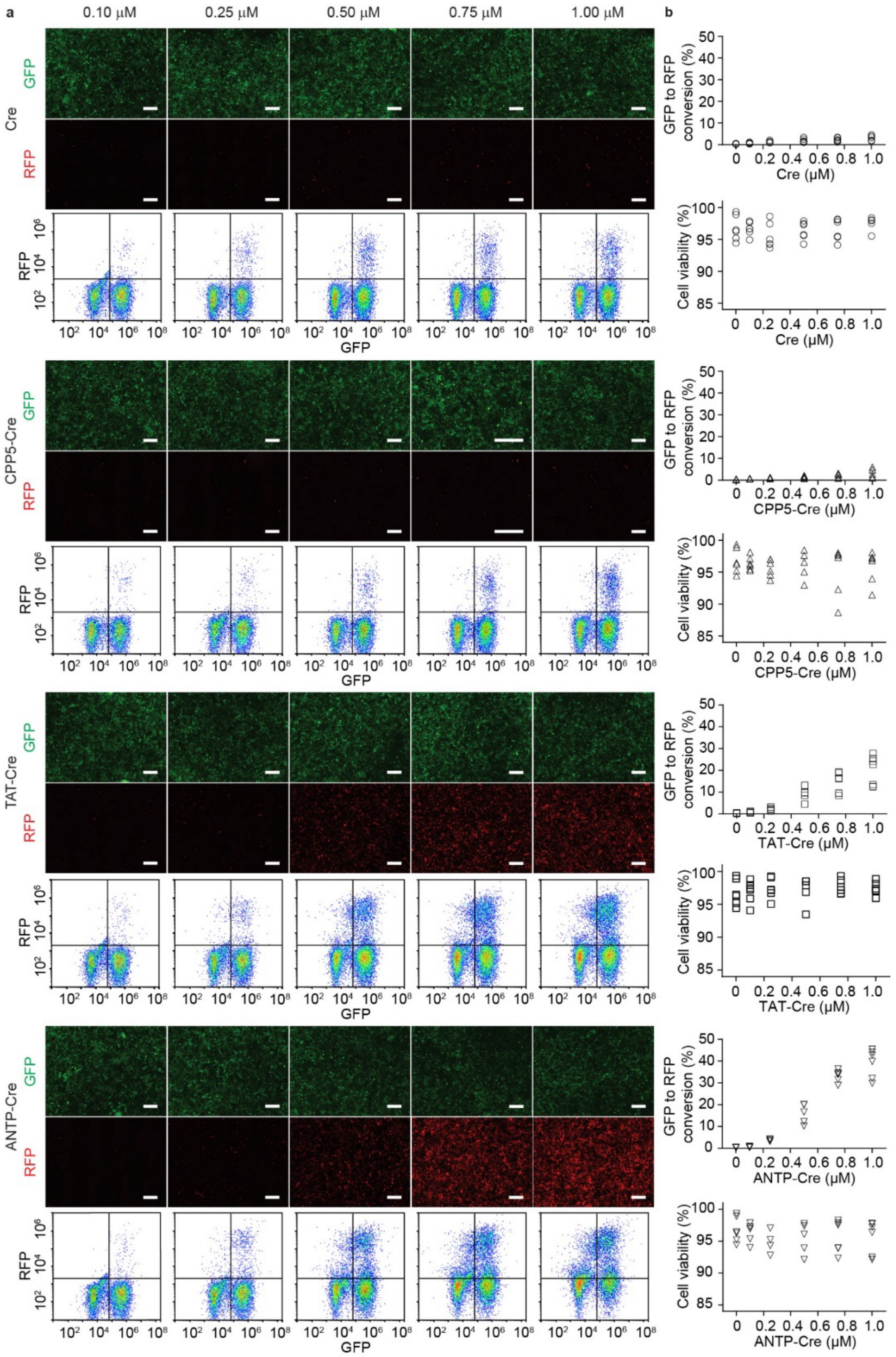

Supplementary Fig. 2 | Titration of HEK293-loxP-GFP-RFP cells with CPP-fused Cre. Cre recombinase, with and without fused cell-penetrating peptides, was delivered to the HEK293-loxP-GFP-RFP cells; and delivery efficiency was analyzed by (**a**) fluorescence microscopy and (**b**) flow cytometry. Cell viability was assessed using inclusion of DAPI. Scale bar: 200 *μ*m. Three experiments with two replicates each, mean ± s.d.

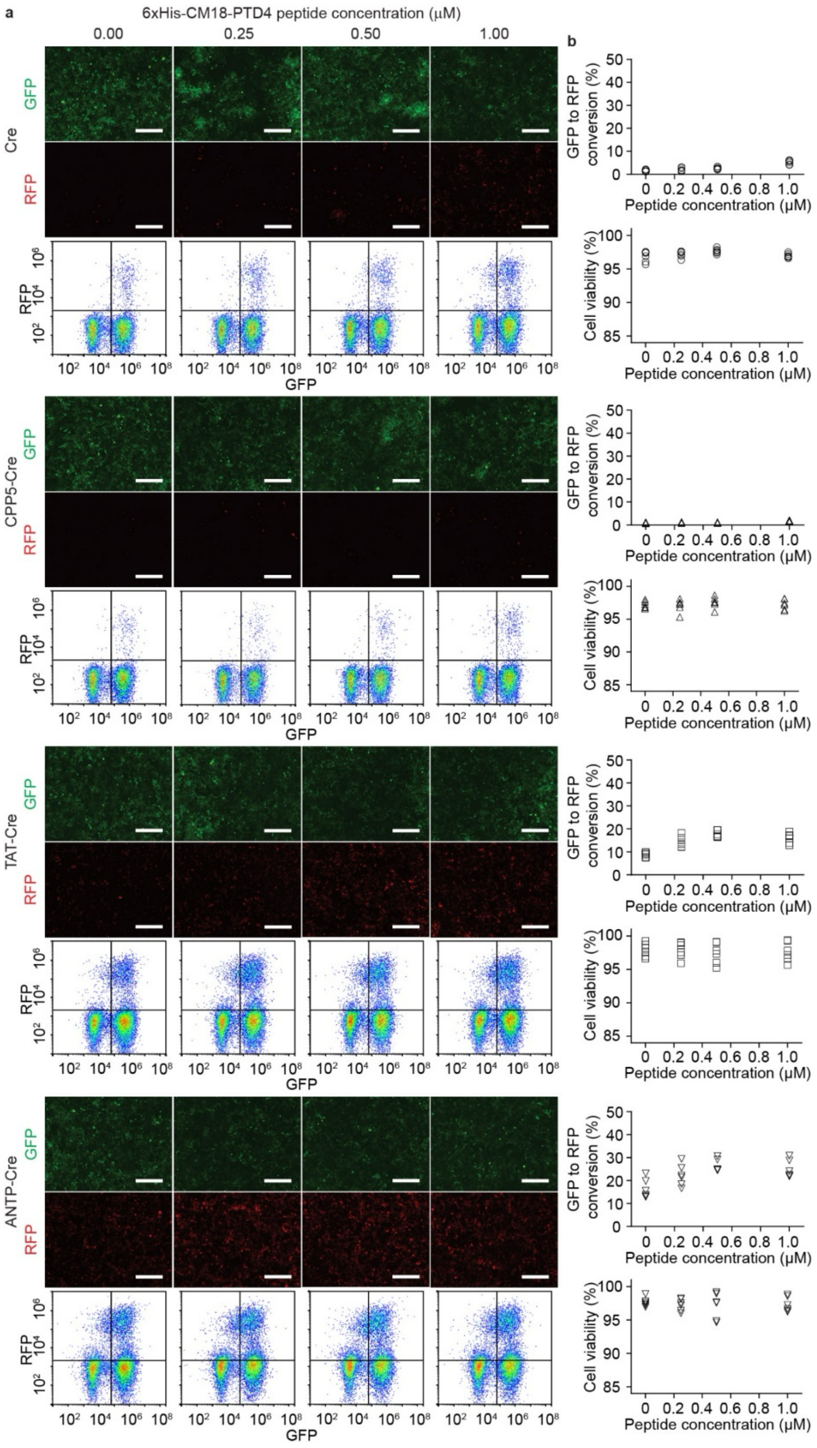

Supplementary Fig. 3 | Titration of HEK293-loxP-GFP-RFP cells with Cre and 6xHis-CM18-PTD4 peptide. Cre recombinase (0.5 μM) with additional 6xHis-CM18-PTD4 peptide was delivered to the HEK293-loxP-GFP-RFP cells, and delivery efficiency was analyzed by (**a**) fluorescence microscopy and (**b**) flow cytometry. Cell viability was assessed according to inclusion of DAPI. Scale bar: 200 μm. Three experiments with two replicates each, mean ± s.d.

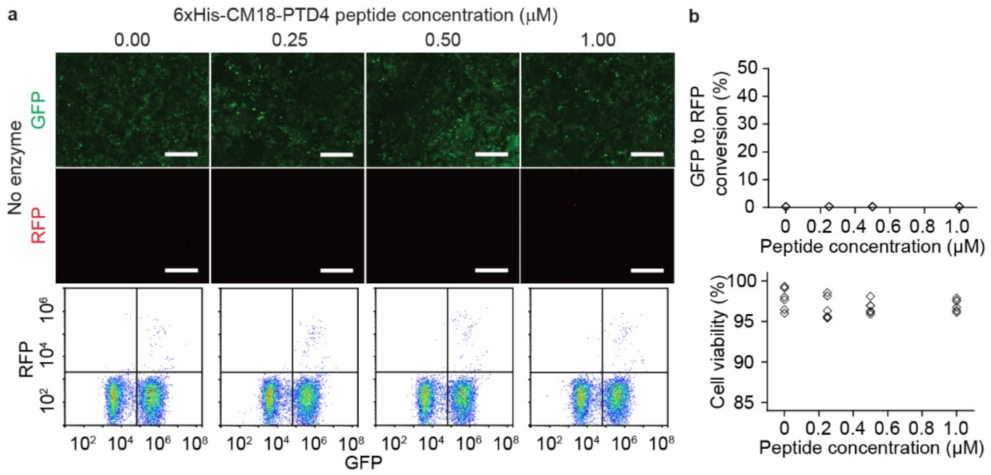

Supplementary Fig. 4 l Minus-enzyme controls for HEK293-loxP-GFP-RFP cells with 6xHis-CM18-PTD4 peptide. The cells were treated with OptiMEM medium without or with additional 6xHis-CM18-PTD4 peptide, analogous to the experiments with Cre recombinase, and analyzed by (**a**) fluorescence microscopy and (**b**) flow cytometry. Scale bar: 200 μm. Three experiments with two replicates each, mean ± s.d.

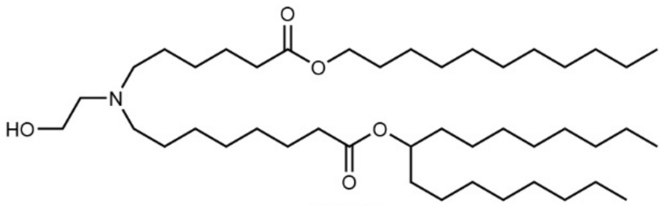

SM102

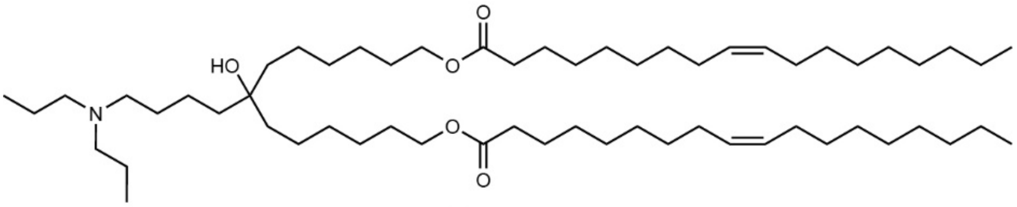

CL4H6

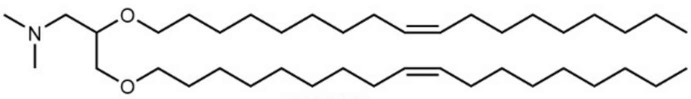

DODMA

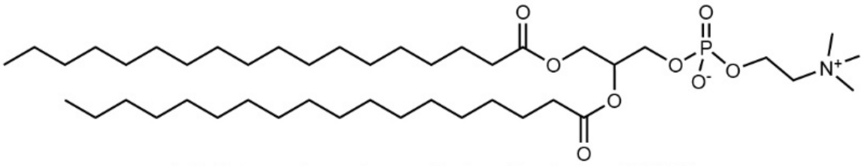

1,2-distearoyl-sn-glycero-3-phosphocholine (DSPC)

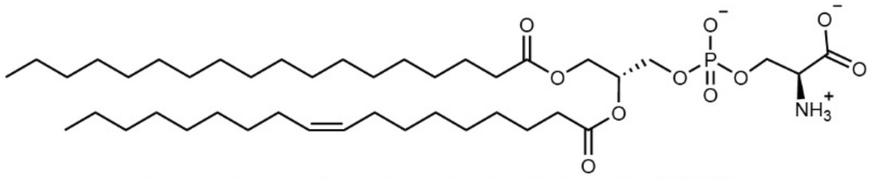

1-stearoyl-2-oleoyl-sn-glycero-3-phospho-L-serine (SOPS)

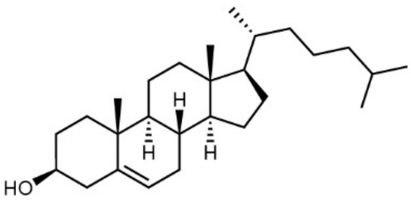

cholesterol

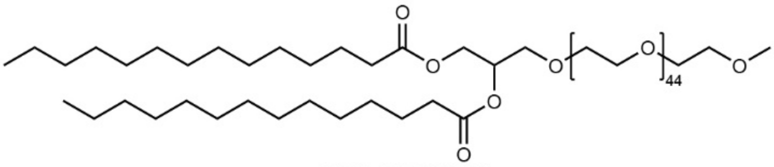

DMG-PEG 2000

Supplementary Fig. 5 | Structures of lipids used in the study.

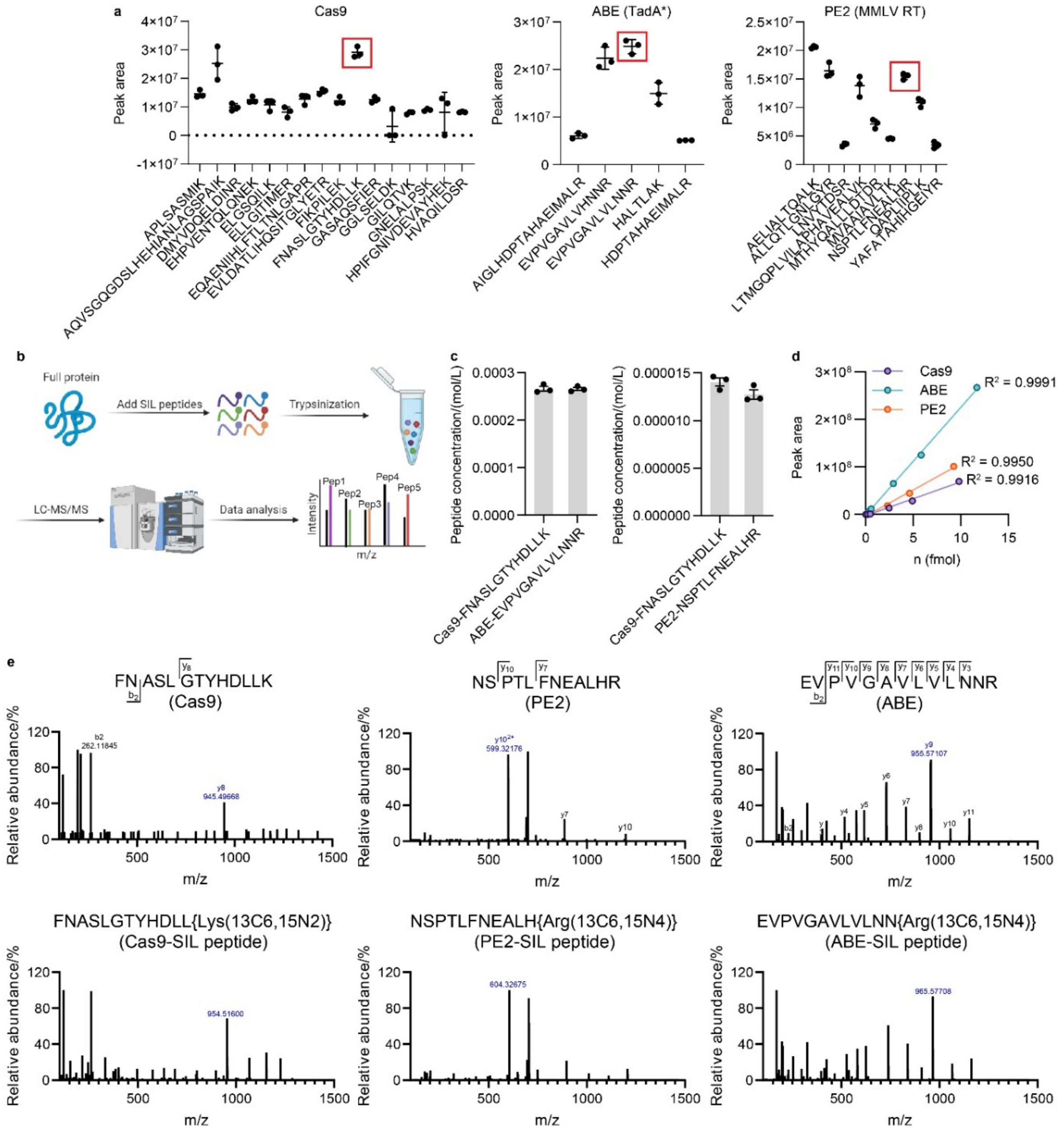

Supplementary Fig. 6 | Mass spectrometric quantification of ABE and PE. (**a**) Reproducibly detected tryptic peptides of Cas9, ABE, and PE2. The peptides selected for quantification are highlighted. (**b**) Workflow for absolute quantification of protein abundance with stable-isotope–labeled (SIL) peptides. Created in BioRender, BioRender.com/z14j704. (**c**) Repeatability of SIL peptide-based quantification in LNP samples. (**d**) Linear response of SIL peptide-based quantification of Cas9, ABE, and PE2. (**e**) The MS2 spectra of the peptides and related SIL peptides for quantification of Cas9, ABE, and PE2.

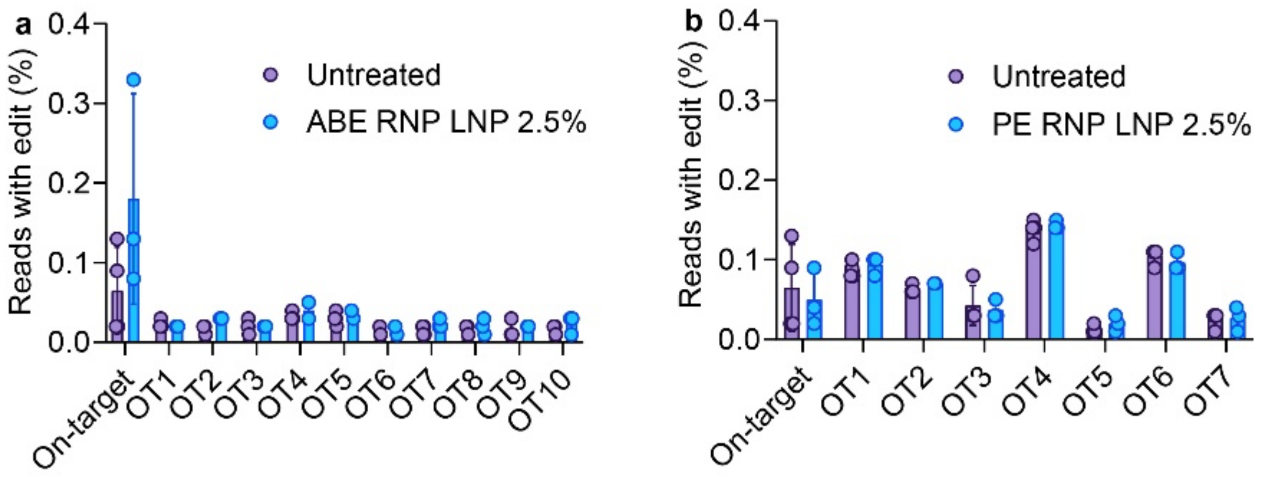

Supplementary Fig. 7 | Off-target analysis of rd12 mice treated with ABE- and PE RNP LNP. (a) Quantification of deamination of target A6 (on-target) or adenosine closest to A5 (off-targets) in genomic DNA isolated from RPE cells of *rd12* mice treated with ABE RNP LNP, 560 ng per eye. **(b)** Quantification of target edit (on-target) or most edited nucleotide (off-targets) in genomic DNA isolated from RPE cells of *rd12* mice treated with PE RNP LNP.

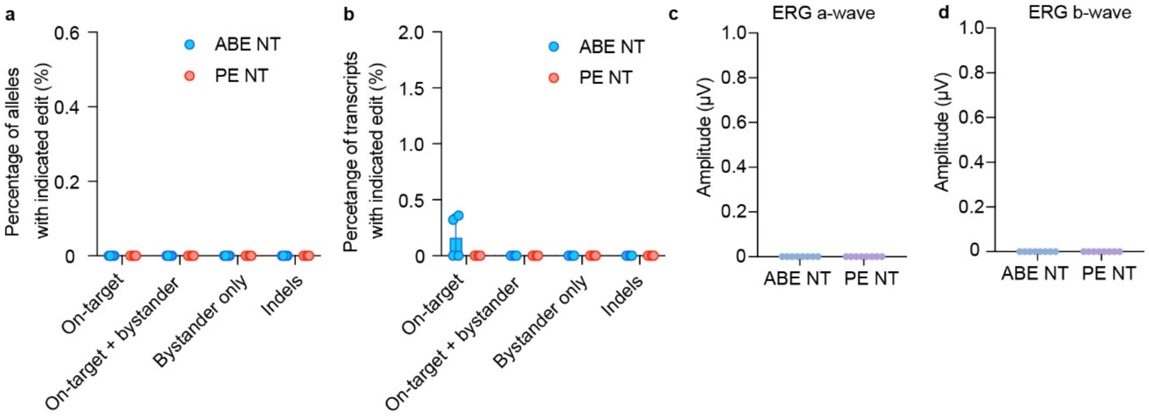

Supplementary Fig. 8 | Non-targeting ABE and PE RNP LNPs do not result in correction of Rpe65 or restore scotopic flash ERG responses. (**a, b**) Next-generation sequencing of **(a)** genomic DNA and (**b**) transcripts to document *Rpe65*-editing outcomes after treatment of *rd12* mice with non-targeting ABE8e RNP LNP or nontargeting PE2 RNP LNP made with 2.5% DMG-PEG 2000. (**c, d**) Scotopic flash ERG a-wave and b-wave amplitudes for *rd12* mice treated with non-targeting ABE RNP LNP and non-targeting PE RNP LNP. The mice received 560 ng of ABE RNP or 655 ng of PE RNP per eye.

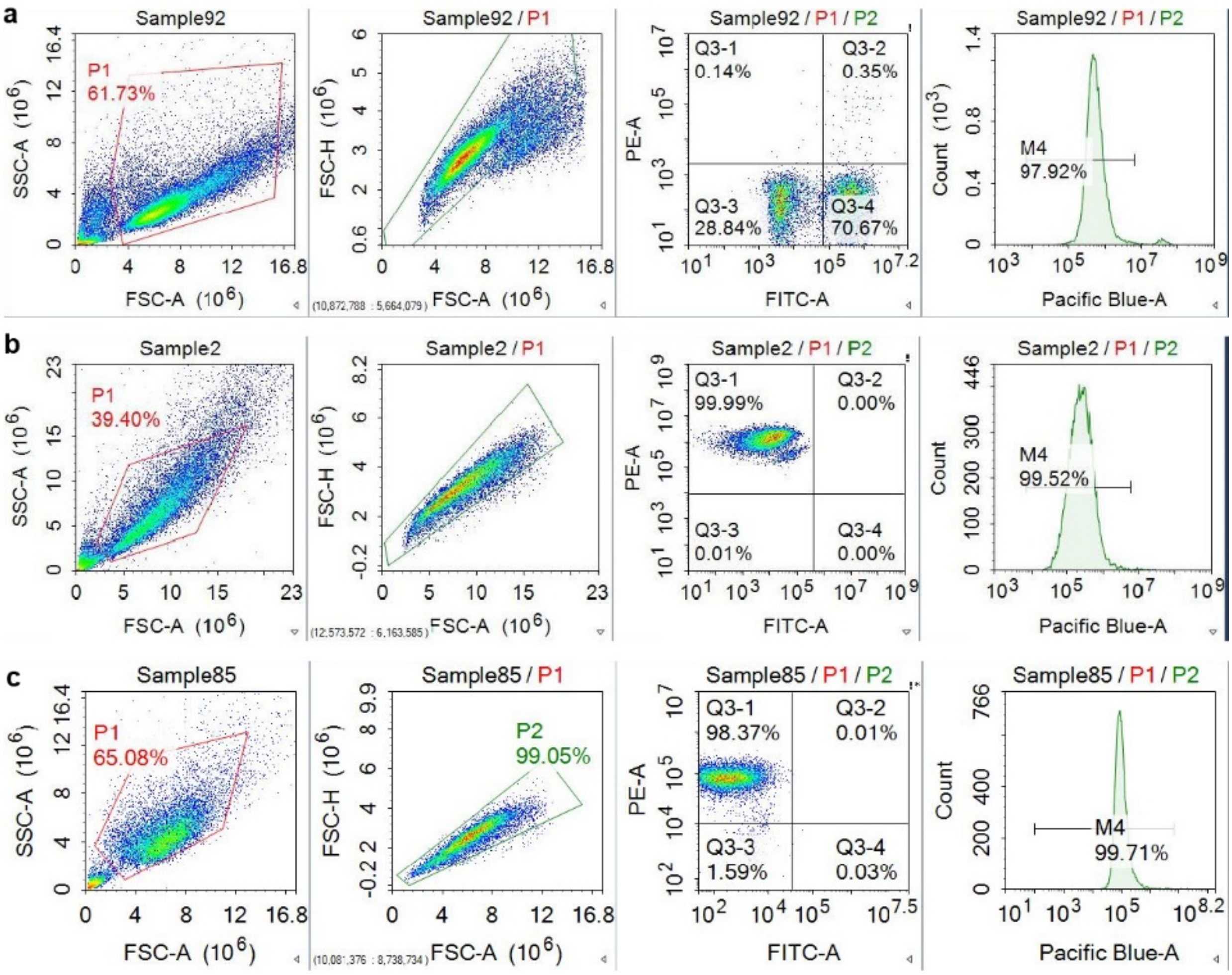

Supplementary Fig. 9 | Flow cytometry gating strategies. (**a**) HEK293-loxP-GFP-RFP cells, (**b**) mT/mG fibroblasts, (**c**) *rd12* reporter cells. The cells were gated on forward and side scatter (FSC-A and CCS-A, respectively) to select intact cells; and forward scatter area (FSC-A) and height (FSC-H) to isolate single cells. Fluorescence of GFP was quantified using a FITC filter (530/30 nm), and fluorescence of RFP, tdTomato, and mCherry was quantified using a PE filter (586/20 nm). Cell viability was estimated by DAPI exclusion using a Pacific Blue filter (445/45 nm).

Supplementary discussion

CPP versus LNP for RNP delivery

In summary, we learned that inclusion of a fused peptide in a protein sequence can be effective for a small, positively charged protein such as Cre (~40 kDa), but not for a large protein such as ABE (187 kDa protein plus 33 kDa sgRNA per monomer), which, as an RNP, is negatively charged. Recently, another group demonstrated extensive optimization of fused CPPs that enabled the delivery of a nuclease Cas9 RNP *in vitro* and *in vivo* [1]. It is important to note, however, that Cas9 nuclease is a monomer,[2] and thus is a better candidate for CPP-mediated delivery than ABE which dimerizes in solution.[3] This self-association can be noted in our SEC profile in **Fig. 1k**, where the ABE protein has an apparent molecular weight of 480 kDa, 2.6-fold higher than the theoretical molecular weight of 187.1 kDa. Non-covalent CPP can be used to overcome these issues *in vitro*, as demonstrated by us and others.[4,5] We were quite disappointed that high concentrations of ABE were still needed, and out of concern about potential toxicity and immunogenicity of ABE, we overcame this by encapsulating RNP into LNP. We justified the shift from CPP- to lipid-based delivery by considering the molecular properties of ABE, the most important of which was the negative charge of the RNP that would enable interactions of ABE with cationic lipids. Indeed, in a reporter cell line, the typical cationic lipid reagent Lipofectamine 3000 enabled effective delivery of ABE RNP at a 25-fold lower concentration than with non-covalent CPP and sucrose. As a next step, we explored the chemically defined LNP technology, which proved to be the most effective both *in vitro* and *in vivo*, achieving superior therapeutic effect at a 15-fold lower concentration of ABE than with CPP or Lipofectamine. Importantly, in contrast to ABE, PE was efficiently delivered only as a lipoplex with Lipofectamine 3000 or encapsulated in LNP, not with non-covalent CPP.

Estimation of number of RNPs in an LNP

We have estimated the approximate number of RNP particles encapsulated per LNP. Two models were considered for this estimation: (1) the solid sphere model, and (2) the liposome model. The solid sphere model posits that lipids, guide RNA, and ABE protein form a compact, water-excluded sphere, and the mass of lipids and RNPs per LNP increases proportionally to volume, cubically with increasing radius. Conversely, the liposome model suggests the formation of a lipid bilayer encapsulating an aqueous interior, thus accommodating ABE proteins within; this model predicts the formation of a higher number of particles, each with fewer protein molecules, due to the internal aqueous space. Here, the mass of lipids and RNPs per LNP increases proportionally to the lipid bilayer area, approximately quadratically with increasing radius of the LNP.

Based on the cryoEM data, we observed condensed product inside the nanoparticles and blebs containing water. Therefore, we favor the liposome model (**Fig. 4k**). To simplify the estimation, we assume that all the lipids in the liposome form a lipid bilayer. The imaged preparation of LNP contained 928.8 μg of lipids (1447 nmol, $8.71 \cdot 10^{17}$ molecules), 119.7 μg of ABE protein (0.64 nmol, $3.85 \cdot 10^{14}$ molecules) and 23.22 μg of sgRNA. Under the assumption that a surface of a lipid head group is 0.71 nm^2,[6] and an average thickness of a lipid monolayer is 5 nm,[7] we estimated that a liposome whose diameter is 100 nm would require 80,000 lipid molecules per particle, and the preparation would contain $1.09 \cdot 10^{13}$ particles. Knowing that $3.85 \cdot 10^{14}$ ABE RNP molecules were used to prepare the LNPs, we can estimate that there are approximately 35 ABE RNP molecules per particle. This number changes to 87 RNP per LNP when the diameter is determined by particle size analysis (154 nm) and used for the calculation.

According to the solid-sphere model, assuming the density of LNP to be 1 g ml^{-1}, the LNPs would occupy a volume of 1.07 μl. A single 100 nm nanoparticle would have a volume of $5.23 \cdot 10^{-16}$ liter; therefore, $2.05 \cdot 10^{12}$ nanoparticles would encapsulate $3.85 \cdot 10^{14}$ molecules of ABE, approximately 188 RNP molecules per nanoparticle (or 687 for particles of d = 154 nm).

Comparison of RNP encapsulation approaches

The typical mRNA LNP encapsulation process involving buffer at pH 4 is not compatible with Cas9/ABE/PE RNP, which denatures and precipitates at low pH. In the approach from Siegwart's group, encapsulation of Cas9 RNP is performed in PBS at neutral pH by the addition of a permanently charged lipid DOTAP, which binds RNP and seeds further interactions with other lipids, including the ionizable lipids C12-200, 5A2, or DLin-MC3-DMA, which are uncharged at this pH. Importantly, the Siegwart group's formulations using a variety of lipid mixes suggested that the LNP technology is quite flexible. Due to the complex nature of ABE and PE compared to Cas9 nuclease used in the Siegwart report,[8] (larger ABE and PE, dimerization of ABE, longer guide RNA for PE), and poor solubility of our RNPs in PBS, we decided to develop a unique

approach. We found that ABE is soluble and active at pH 6 and higher, enabling us to encapsulate RNPs at pH 6. Based on our experience with liposomes and mRNA nanoparticles, we utilized monoprotic Tris base and acetic acid as the buffer. We included 10% sucrose in all solutions to prevent aggregation of ABE RNP, which occurs at ABE RNP concentrations above 4 μM without sucrose. Next, to provide a driving force for interactions between the positively charged lipids and the negatively charged guide RNA and acidic side chains of ABE/PE, we used ionizable lipids with pKa values significantly above 6.0, so that they would be at least partially protonated and bear significant positive charge at pH 6. We kept the ionic strength of our encapsulation buffer low (10 mM NaCl instead of ~137 mM NaCl plus other salts in PBS) to further strengthen the interactions between lipids and our cargo. All these considerations helped us achieve successful encapsulation of first ABE, and then PE, using three suitable ionizable lipids, SM102, CL4H6 and DODMA. SM102 was chosen for further optimization due to toxicity of DODMA and poor reproducibility with CL4H6, including optimization of the lipid:RNA ratio and PEG content; we settled on and chose the 40:1 weight ratio and 2.5% DMG-PEG 2000 in the lipid mixture.

Comparing the two approaches, the LNPs with permanently cationic lipids made by Wei *et al.*[8] had a particle size of 200 nm or more, and polydispersity index mostly above 0.1 (**Fig. 2 therein**). Our optimized RNP LNP had a particle size consistently below 200 nm and a polydispersity index predominantly below 0.1. The low diameter of our optimized LNPs formed at pH 6 enables sterile filtration through 0.22 um filters, which is a significant advantage for clinical translation. In terms of activity, Siegwart-group LNPs were effective at a tested concentration of 9 nM RNP, whereas our ABE LNPs and PE LNPs converted 100% of the reporter cells at as low as 5 nM RNP (**Fig. 4j, Fig. 5c**). Our LNPs can be stored frozen for at least 6 months without loss of activity, which was not demonstrated for SORT LNP. Overall, we conclude that while distinct, our, Siegwart's and others' approaches are viable starting points for further development of Cas9-RNP delivery technology [9], and our formulation is uniquely optimized for the more complex and challenging delivery of ABE- and PE-RNPs.

Supplementary tables

Supplementary Table 1 I Oligonucleotides used in this work.

Name	Sequence 5' – 3'	Remarks
ANTP-Or_F	CATGGGCCGCCAGATCAAGATTTGGTTCCAGAATCGTCGCATGAAGTGGAAGAAAGGCCA	
ANTP-Or_R	TATGGCCTTTCTTCCACTTCATGCGACGATTCTGGAACCAAATCTTGATCTGGCGGCC	
RH011_CPP5_F	TATGGGCAAACTGCCGGTTATGGGCCA	Introduction of CPP5 peptide into ABE
RH012_CPP5_R	TATGGCCCATAACCGGCAGTTTGCCCA	Introduction of CPP5 peptide into ABE
RH013_TAT_F	TATGGGCAGGAAGAAGCGGAGACAGCGACGAAGAGGCCA	Introduction of TAT peptide into ABE
RH014_TAT_R	TATGGCCTCTTCGTCGCTGTCTCCGCTTCTTCCTGCCCA	Introduction of TAT peptide into ABE
RH017_Antp_F	TATGGGCCGCCAGATAAAGATTTGGTTCCAGAATCGGCGCATGAAGTGGAAGAAGG GCCA	Introduction of Antp peptide into ABE
RH018_Antp_R	TATGGCCCTTCTTCCACTTCATGCGCCGATTCTGGAACCAAATCTTTATCTGGCGGCCCA	Introduction of Antp peptide into ABE
Cre_USER_F	AGCGGAAAGUCAGCAATTTACTGACCGTA	USER cloning
Cre_USER_R	AGGGAAAAAGAUTTAGTCGCCATCTTCCAGCA	USER cloning
gag_USER_F	ATCTTTTTCCCUCTGCCAAAAATTATGGGGAC	USER cloning
gag_USER_R	ACTTTCCGCUTCTTCTTTGGTGACTCGAA	USER cloning
RH008_ABE_F	GATTCCCCTCTGGCTCACTGGCAGTCTCCTCTGATGTGGGCCAGGGCTCTTTGAAGTTGG	ABE/PE assay substrate
RH010FAM_ABE_contR	[6FAM]CCAACTTCAAAGAGCCCTGGCCCACATCAGAGGAGACTGCCAGTGAGCCAGAGGGGAATC	ABE/PE assay substrate
RH009FAM_ABE_R	[6FAM]CCAACTTCAAAGAGCCCTGGCCCACATCIGAGGAGACTGCCAGTGAGCCAGAGGGGAATC	ABE/PE assay positive control

Supplementary Table 2 I PCR primers used in this work.

Name	Sequence	Remarks
rd12_genomic_HTS_F	ACACTCTTTCCCTACACGACGCTCTTCCGATCTNNNNTGATATCTCACTTTGCTGCAGG	PCR1
rd12_genomic_HTS_R	TGGAGTTCAGACGTGTGCTCTTCCGATCTATGGCTAGACCATGAAGAAAGAAG	PCR1
rd12_cDNA_HTS_F	ACACTCTTTCCCTACACGACGCTCTTCCGATCTNNNNACCCTGCTGGTGGCTACAAGAA	PCR1
rd12_cDNA_HTS_R	TGGAGTTCAGACGTGTGCTCTTCCGATCTGCAGGGGTCTGGGAAAGCACAG	PCR1
mCherry_HTS_F	ACACTCTTTCCCTACACGACGCTCTTCCGATCTNNNNGAGGGCCGCCACTCCACCGG	PCR1
eGFP_HTS_R	TGGAGTTCAGACGTGTGCTCTTCCGATCTCACGCCTGTAAACAGTTCCTCGCC	PCR1
TEV_1D4_SDM_F	GAGACAAGCCAGGTGGCTCCGGCGTAAGGTTAGAGCGGCCGC	SDM of protein tag
TEV_1D4_SDM_R	AGTTGATTGAAAATATAGATTTTCCACCTTGCGTTTCTTTTTCGG	SDM of protein tag
RH019_CPP5F	TGGGCAAACTGCCGGTTATGG	Colony PCR
RH020_TATF	GAAGCGGAGACAGCGACGAAG	Colony PCR
RH021_ANTPF	CCAGAATCGGCGCATGAAGTGG	Colony PCR
PD881F	CGAATTCAGGCGCTTTTTAG	Sanger sequencing
RH022_ABER	CTGCATAACCAAACCACCCTGGC	Colony PCR

Supplementary Table 3 I Sequences of selected SIL peptides, purity, concentration of stock solution and spiking solution.

Protein	Sequences of SIL peptides	Purity/ %	Stock solution (mg ml^{-1})	Spiking Solution (mg ml^{-1})
Cas9	FNASLGTYHDLL[Lys(^{13}C6, ^{15}N2)]	91.2	0.999	0.999
ABE	EVPVGAVLVLNN[Arg(^{13}C6, ^{15}N4)]	97.6	1.041	1.041
PE2	NSPTLFNEALH[Arg(^{13}C6, ^{15}N4)]	81.0	1.005	1.005

Supplementary Table 4 | Sequences of primers used for off-target analyses.

Name	Sequence	Locus
NW-Rd12_ontarg_F'	ACACTCTTTCCCTACACGACGCTCTTCCGATCTTGATATCTCACTTTGCTGCAGG	Rd12 On-target (genomic)
NW-Rd12_ontarg_R'	GACTGGAGTTCAGACGTGTGCTCTTCCGATCTAGGAGAAGAACTGAGATAGGCT	Rd12 On-target (genomic)
NW_ret-OT1-F	ACACTCTTTCCCTACACGACGCTCTTCCGATCTNNNNTTGCCAACAGGTTCTCCTATC	ABE OTS 1
NW_ret-OT1-R	TGGAGTTCAGACGTGTGCTCTTCCGATCTCTGGCAGAACTGAACGAGG	ABE OTS 1
NW_ret-OT2-F	ACACTCTTTCCCTACACGACGCTCTTCCGATCTNNNNGTGATCACAGAGGTGGGC	ABE OTS 2
NW_ret-OT2-R	TGGAGTTCAGACGTGTGCTCTTCCGATCTCATCAGTGACTTGGGATGATAGA	ABE OTS 2
NW_ret-OT3-F	ACACTCTTTCCCTACACGACGCTCTTCCGATCTNNNNCCATCTGCCACCACTCAC	ABE OTS 3
NW_ret-OT3-R	TGGAGTTCAGACGTGTGCTCTTCCGATCTGATCTAGTAGGCCTTTCAGGCATGA	ABE OTS 3
NW_ret-OT4-F	ACACTCTTTCCCTACACGACGCTCTTCCGATCTNNNNGGAATGAGTCTGTGAGATCGC	ABE OTS 4
NW_ret-OT4-R	TGGAGTTCAGACGTGTGCTCTTCCGATCTCTCATTCTGTAGATCAGGCTGG	ABE OTS 4
NW_ret-OT5-F	ACACTCTTTCCCTACACGACGCTCTTCCGATCTNNNNGCTAAGTCAGCCCTTCGTTG	ABE OTS 5
NW_ret-OT5-R	TGGAGTTCAGACGTGTGCTCTTCCGATCTTACCTCCCAGTGATGGGACT	ABE OTS 5
NW_ret-OT6-F	ACACTCTTTCCCTACACGACGCTCTTCCGATCTNNNNTCTCACAGCAAATCACTGATCTA	ABE OTS 6
NW_ret-OT6-R	TGGAGTTCAGACGTGTGCTCTTCCGATCTTATCCTACTCAGTTCTGCAGATA	ABE OTS 6
NW_ret-OT7-F	ACACTCTTTCCCTACACGACGCTCTTCCGATCTNNNNAAGTGGCCAACCGTGCGA	ABE OTS 7
NW_ret-OT7-R	TGGAGTTCAGACGTGTGCTCTTCCGATCTTTCTAACACTTCAATTCAGGTGG	ABE OTS 7
NW_ret-OT8-F	ACACTCTTTCCCTACACGACGCTCTTCCGATCTNNNNCAGGACAAGTTGTCCCTGGGC	ABE OTS 8
NW_ret-OT8-R	TGGAGTTCAGACGTGTGCTCTTCCGATCTGTGGGAGTGTTAAAGACAGCG	ABE OTS 8
NW_ret-OT9-F	ACACTCTTTCCCTACACGACGCTCTTCCGATCTNNNNTGGCAGATAGGAGACACTTCT	ABE OTS 9
NW_ret-OT9-R	TGGAGTTCAGACGTGTGCTCTTCCGATCTCCTGATAAACCCTTCCTTGAC	ABE OTS 9
NW_ret-OT10-F	ACACTCTTTCCCTACACGACGCTCTTCCGATCTNNNNGAATGCGGAAGACATGTTCTATT C	ABE OTS 10
NW_ret-OT10-R	TGGAGTTCAGACGTGTGCTCTTCCGATCTCATCAGAGGATTCCCAACGC	ABE OTS 10
NW-Rd12peg_OT02_primers1_fwd	ACACTCTTTCCCTACACGACGCTCTTCCGATCTNNNNCCACATTGTTGGAGCTGCCGGT	PE OTS 1
NW-Rd12peg_OT03_primers1_fwd	ACACTCTTTCCCTACACGACGCTCTTCCGATCTNNNNGGGTCTCTCTTCCCCATGTCTCT A	PE OTS 2
NW-Rd12peg_OT04_primers1_fwd	ACACTCTTTCCCTACACGACGCTCTTCCGATCTNNNNCCTGGCTCCTTCTGAGCACACTG	PE OTS 3
NW-Rd12peg_OT07_primers1_fwd	ACACTCTTTCCCTACACGACGCTCTTCCGATCTNNNNCCACCCCATCACATCCTCTGCTG	PE OTS 4
NW-Rd12peg_OT08_primers1_fwd	ACACTCTTTCCCTACACGACGCTCTTCCGATCTNNNNGGTACACTGTAGGCTGGGGACC	PE OTS 5
NW-Rd12peg_OT09_primers1_fwd	ACACTCTTTCCCTACACGACGCTCTTCCGATCTNNNNACTCTTGGCACCCACTGTAAGTC T	PE OTS 6
NW-Rd12peg_OT10_primers1_fwd	ACACTCTTTCCCTACACGACGCTCTTCCGATCTNNNNGTTGGGGCTTCCTGGTTCAA	PE OTS 7
NW-Rd12peg_OT02_primers1_rvs	TGGAGTTCAGACGTGTGCTCTTCCGATCTAGCTCTCCAGTGCTCACACTCAA	PE OTS 1
NW-Rd12peg_OT03_primers1_rvs	TGGAGTTCAGACGTGTGCTCTTCCGATCTGGTTCCGATGCTCACCTCCATGTC	PE OTS 2
NW-Rd12peg_OT04_primers1_rvs	TGGAGTTCAGACGTGTGCTCTTCCGATCTAAAAGCAAGGCTTTGTTCGGGCCT	PE OTS 3
NW-Rd12peg_OT07_primers1_rvs	TGGAGTTCAGACGTGTGCTCTTCCGATCTATGATATCCACACCCGGCTCAAAGG	PE OTS 4
NW-Rd12peg_OT08_primers1_rvs	TGGAGTTCAGACGTGTGCTCTTCCGATCTGTGTCTCCTTAGAGGCAGCAAGAGT	PE OTS 5
NW-Rd12peg_OT09_primers1_rvs	TGGAGTTCAGACGTGTGCTCTTCCGATCTCCAGGCCCCACCCCTATAAGGTA	PE OTS 6
NW-Rd12peg_OT10_primers1_rvs	TGGAGTTCAGACGTGTGCTCTTCCGATCTCCCTTAATAACATAAAAGCTGTGGT	PE OTS 7

Supplementary sequences

Peptide sequences
>6xHis-CM18-PTD4 peptide
HHHHHHKWKLFKKIGAVLKVLTTGYARAAARQARA

>1D4 peptide
TETSQVAPA

RNA sequences
r = Ribose RNA bases
m = 2' O-methyl RNA bases
m_* = Phosphorothioated 2'-O-methyl RNA bases

>rd12-A6-sgRNA sequence
ACATCAGAGGAGACTGCCAGGTTTTAGAGCTAGAAATAGCAAGTTAAAATAAGGCTAGTCCGTTATCAA
CTTGAAAAAGTGGCACCGAGTCGGTGC

>rd12-A6-sgRNA highly modified
mA*mC*mA*rUrCrArGrArGrGrArGrArCrUrGrCrCrArGrGrUrUrUrUrArGrAmGmCmUmAmGmAmAmAmUmA
mGmCrArArGrUrUrArArArArUrArArGrGrCrUrArGrUrCrCrGrUrUrArUrCrAmAmCmUmUmGmAmAmAmAmA
mGmUmGmGmCmAmCmCmGmAmGmUmCmGmGmUmGmCmUmU*mU*mU*rU

>rd12-A6-sgRNA end-modified
mA*mC*mA*rUrCrArGrArGrGrArGrArCrUrGrCrCrArGrGrUrUrUrUrArGrArGrCrUrArGrArArArUrArGrCrArArGr
UrUrArArArArUrArArGrGrCrUrArGrUrCrCrGrUrUrArUrCrArArCrUrUrGrArArArArArGrUrGrGrCrArCrCrGrArGr
UrCrGrGrUrGrCrUmU*mU*mU*rU

>rd12-epegRNA sequence
AGAGCCCTGGCCCACATCAGGTTTTAGAGCTAGAAATAGCAAGTTAAAATAAGGCTAGTCCGTTATCAA
CTTGAAAAAGTGGCACCGAGTCGGTGCAGTCTCCTCCGATGTGGGCCGCGGTTCTATCTAGTTACGCG
TTAAACCAACTAGAA

>rd12_epegRNA
mA*mG*mA*rGrCrCrCrUrGrGrCrCrCrArCrArUrCrArGrGrUrUrUrUrArGrArGrCrUrArGrArArArUrArGrCrArArG
rUrUrArArArArUrArArGrGrCrUrArGrUrCrCrGrUrUrArUrCrArArCrUrUrGrArArArArArGrUrGrGrCrArCrCrGrArG
rUrCrGrGrUrGrCrArGrUrCrUrCrCrUrCrCrGrArUrGrUrGrGrGrCrCrGrCrGrGrUrUrCrUrArUrCrUrArGrUrUrArC
rGrCrGrUrUrArArArCrCrArArCrUrArGrArAmUmU*mU*mU

>ABE_NT_RNA
mG*mC*mG*rArGrGrUrArUrUrCrGrGrCrUrCrCrGrCrGrGrUrUrUrUrArGrAmGmCmUmAmGmAmAmAmUmA
mGmCrArArGrUrUrArArArArUrArArGrGrCrUrArGrUrCrCrGrUrUrArUrCrAmAmCmUmUmGmAmAmAmAmA
mGmUmGmGmCmAmCmCmGmAmGmUmCmGmGmUmGmCmUmU*mU*mU*rU

>PE_NT_RNA
mC*mU*mU*rCrUrCrCrArGrArGrArGrUrGrCrCrCrUrUrGrUrUrUrUrArGrArGrCrUrArGrArArArUrArGrCrArArG
rUrUrArArArArUrArArGrGrCrUrArGrUrCrCrGrUrUrArUrCrArArCrUrUrGrArArArArArGrUrGrGrCrArCrCrGrArG
rUrCrGrGrUrGrCrArCrCrArGrArCrCrArGrUrArArGrUrCrCrCrArArGrGrGrCrArCrUrCrUrCrUrGrGrCrGrCrGrGr
UrUrCrUrArUrCrUrArGrUrUrArCrGrCrGrUrUrArArArCrCrArArCrUrArGrArAmUmU*mU*mU

Plasmid DNA sequences
>pN-Cre
TGGCGAATGGGACGCGCCCTGTAGCGGCGCATTAAGCGCGGCGGGTGTGGTGGTTACGCGCAGCGT
GACCGCTACACTTGCCAGCGCCCTAGCGCCCGCTCCTTTCGCTTTCTTCCCTTCCTTTCTCGCCACGTT
CGCCGGCTTTCCCCGTCAAGCTCTAAATCGGGGGCTCCCTTTAGGGTTCCGATTTAGTGCTTTACGGCA
CCTCGACCCCAAAAAACTTGATTAGGGTGATGGTTCACGTAGTGGGCCATCGCCCTGATAGACGGTTTT
TCGCCCTTTGACGTTGGAGTCCACGTTCTTTAATAGTGGACTCTTGTTCCAAACTGGAACAACACTCAAC
CCTATCTCGGTCTATTCTTTTGATTTATAAGGGATTTTGCCGATTTCGGCCTATTGGTTAAAAAATGAGCT
GATTTAACAAAAATTTAACGCGAATTTTAACAAAATATTAACGTTTACAATTTCAGGTGGCACTTTTCGGG

GAAATGTGCGCGGAACCCCTATTTGTTTATTTTTCTAAATACATTCAAATATGTATCCGCTCATGAATTAA
TTCTTAGAAAAACTCATCGAGCATCAAATGAAACTGCAATTTATTCATATCAGGATTATCAATACCATATT
TTTGAAAAAGCCGTTTCTGTAATGAAGGAGAAAACTCACCGAGGCAGTTCCATAGGATGGCAAGATCCT
GGTATCGGTCTGCGATTCCGACTCGTCCAACATCAATACAACCTATTAATTTCCCCTCGTCAAAAATAAG
GTTATCAAGTGAGAAATCACCATGAGTGACGACTGAATCCGGTGAGAATGGCAAAAGTTTATGCATTTCT
TTCCAGACTTGTTCAACAGGCCAGCCATTACGCTCGTCATCAAAATCACTCGCATCAACCAAACCGTTAT
TCATTCGTGATTGCGCCTGAGCGAGACGAAATACGCGATCGCTGTTAAAAGGACAATTACAAACAGGAA
TCGAATGCAACCGGCGCAGGAACACTGCCAGCGCATCAACAATATTTTCACCTGAATCAGGATATTCTT
CTAATACCTGGAATGCTGTTTTCCCGGGGATCGCAGTGGTGAGTAACCATGCATCATCAGGAGTACGGA
TAAAATGCTTGATGGTCGGAAGAGGCATAAATTCCGTCAGCCAGTTTAGTCTGACCATCTCATCTGTAAC
ATCATTGGCAACGCTACCTTTGCCATGTTTCAGAAACAACTCTGGCGCATCGGGCTTCCCATACAATCG
ATAGATTGTCGCACCTGATTGCCCGACATTATCGCGAGCCCATTTATACCCATATAAATCAGCATCCATG
TTGGAATTTAATCGCGGCCTAGAGCAAGACGTTTCCCGTTGAATATGGCTCATAACACCCCTTGTATTAC
TGTTTATGTAAGCAGACAGTTTTATTGTTCATGACCAAAATCCCTTAACGTGAGTTTTCGTTCCACTGAGC
GTCAGACCCCGTAGAAAAGATCAAAGGATCTTCTTGAGATCCTTTTTTTCTGCGCGTAATCTGCTGCTTG
CAAACAAAAAAACCACCGCTACCAGCGGTGGTTTGTTTGCCGGATCAAGAGCTACCAACTCTTTTTCCG
AAGGTAACTGGCTTCAGCAGAGCGCAGATACCAAATACTGTCCTTCTAGTGTAGCCGTAGTTAGGCCAC
CACTTCAAGAACTCTGTAGCACCGCCTACATACCTCGCTCTGCTAATCCTGTTACCAGTGGCTGCTGCC
AGTGGCGATAAGTCGTGTCTTACCGGGTTGGACTCAAGACGATAGTTACCGGATAAGGCGCAGCGGTC
GGGCTGAACGGGGGGTTCGTGCACACAGCCCAGCTTGGAGCGAACGACCTACACCGAACTGAGATAC
CTACAGCGTGAGCTATGAGAAAGCGCCACGCTTCCCGAAGGGAGAAAGGCGGACAGGTATCCGGTAA
GCGGCAGGGTCGGAACAGGAGAGCGCACGAGGGAGCTTCCAGGGGGAAACGCCTGGTATCTTTATAG
TCCTGTCGGGTTTCGCCACCTCTGACTTGAGCGTCGATTTTTGTGATGCTCGTCAGGGGGGCGGAGCC
TATGGAAAAACGCCAGCAACGCGGCCTTTTTACGGTTCCTGGCCTTTTGCTGGCCTTTTGCTCACATGT
TCTTTCCTGCGTTATCCCCTGATTCTGTGGATAACCGTATTACCGCCTTTGAGTGAGCTGATACCGCTCG
CCGCAGCCGAACGACCGAGCGCAGCGAGTCAGTGAGCGAGGAAGCGGAAGAGCGCCTGATGCGGTA
TTTTCTCCTTACGCATCTGTGCGGTATTTCACACCGCATATATGGTGCACTCTCAGTACAATCTGCTCTG
ATGCCGCATAGTTAAGCCAGTATACACTCCGCTATCGCTACGTGACTGGGTCATGGCTGCGCCCCGAC
ACCCGCCAACACCCGCTGACGCGCCCTGACGGGCTTGTCTGCTCCCGGCATCCGCTTACAGACAAGCT
GTGACCGTCTCCGGGAGCTGCATGTGTCAGAGGTTTTCACCGTCATCACCGAAACGCGCGAGGCAGCT
GCGGTAAAGCTCATCAGCGTGGTCGTGAAGCGATTCACAGATGTCTGCCTGTTCATCCGCGTCCAGCT
CGTTGAGTTTCTCCAGAAGCGTTAATGTCTGGCTTCTGATAAAGCGGGCCATGTTAAGGGCGGTTTTTT
CCTGTTTGGTCACTGATGCCTCCGTGTAAGGGGGATTTCTGTTCATGGGGGTAATGATACCGATGAAAC
GAGAGAGGATGCTCACGATACGGGTTACTGATGATGAACATGCCCGGTTACTGGAACGTTGTGAGGGT
AAACAACTGGCGGTATGGATGCGGCGGGACCAGAGAAAAATCACTCAGGGTCAATGCCAGCGCTTCGT
TAATACAGATGTAGGTGTTCCACAGGGTAGCCAGCAGCATCCTGCGATGCAGATCCGGAACATAATGGT
GCAGGGCGCTGACTTCCGCGTTTCCAGACTTTACGAAACACGGAAACCGAAGACCATTCATGTTGTTGC
TCAGGTCGCAGACGTTTTGCAGCAGCAGTCGCTTCACGTTCGCTCGCGTATCGGTGATTCATTCTGCTA
ACCAGTAAGGCAACCCCGCCAGCCTAGCCGGGTCCTCAACGACAGGAGCACGATCATGCGCACCCGT
GGGGCCGCCATGCCGGCGATAATGGCCTGCTTCTCGCCGAAACGTTTGGTGGCGGGACCAGTGACGA
AGGCTTGAGCGAGGGCGTGCAAGATTCCGAATACCGCAAGCGACAGGCCGATCATCGTCGCGCTCCA
GCGAAAGCGGTCCTCGCCGAAAATGACCCAGAGCGCTGCCGGCACCTGTCCTACGAGTTGCATGATAA
AGAAGACAGTCATAAGTGCGGCGACGATAGTCATGCCCCGCGCCCACCGGAAGGAGCTGACTGGGTT
GAAGGCTCTCAAGGGCATCGGTCGAGATCCCGGTGCCTAATGAGTGAGCTAACTTACATTAATTGCGTT
GCGCTCACTGCCCGCTTTCCAGTCGGGAAACCTGTCGTGCCAGCTGCATTAATGAATCGGCCAACGCG
CGGGGAGAGGCGGTTTGCGTATTGGGCGCCAGGGTGGTTTTTCTTTTCACCAGTGAGACGGGCAACAG
CTGATTGCCCTTCACCGCCTGGCCCTGAGAGAGTTGCAGCAAGCGGTCCACGCTGGTTTGCCCCAGCA
GGCGAAAATCCTGTTTGATGGTGGTTAACGGCGGGATATAACATGAGCTGTCTTCGGTATCGTCGTATC
CCACTACCGAGATATCCGCACCAACGCGCAGCCCGGACTCGGTAATGGCGCGCATTGCGCCCAGCGC
CATCTGATCGTTGGCAACCAGCATCGCAGTGGGAACGATGCCCTCATTCAGCATTTGCATGGTTTGTTG
AAAACCGGACATGGCACTCCAGTCGCCTTCCCGTTCCGCTATCGGCTGAATTTGATTGCGAGTGAGATA
TTTATGCCAGCCAGCCAGACGCAGACGCGCCGAGACAGAACTTAATGGGCCCGCTAACAGCGCGATTT
GCTGGTGACCCAATGCGACCAGATGCTCCACGCCCAGTCGCGTACCGTCTTCATGGGAGAAAATAATA
CTGTTGATGGGTGTCTGGTCAGAGACATCAAGAAATAACGCCGGAACATTAGTGCAGGCAGCTTCCACA
GCAATGGCATCCTGGTCATCCAGCGGATAGTTAATGATCAGCCCACTGACGCGTTGCGCGAGAAGATT
GTGCACCGCCGCTTTACAGGCTTCGACGCCGCTTCGTTCTACCATCGACACCACCACGCTGGCACCCA
GTTGATCGGCGCGAGATTTAATCGCCGCGACAATTTGCGACGGCGCGTGCAGGGCCAGACTGGAGGT
GGCAACGCCAATCAGCAACGACTGTTTGCCCGCCAGTTGTTGTGCCACGCGGTTGGGAATGTAATTCA
GCTCCGCCATCGCCGCTTCCACTTTTTCCCGCGTTTTCGCAGAAACGTGGCTGGCCTGGTTCACCACG

CGGGAAACGGTCTGATAAGAGACACCGGCATACTCTGCGACATCGTATAACGTTACTGGTTTCACATTC
ACCACCCTGAATTGACTCTCTTCCGGGCGCTATCATGCCATACCGCGAAAGGTTTTGCGCCATTCGATG
GTGTCCGGGATCTCGACGCTCTCCCTTATGCGACTCCTGCATTAGGAAGCAGCCCAGTAGTAGGTTGA
GGCCGTTGAGCACCGCCGCCGCAAGGAATGGTGCATGCAAGGAGATGGCGCCCAACAGTCCCCCGGC
CACGGGGCCTGCCACCATACCCACGCCGAAACAAGCGCTCATGAGCCCGAAGTGGCGAGCCCGATCT
TCCCCATCGGTGATGTCGGCGATATAGGCGCCAGCAACCGCACCTGTGGCGCCGGTGATGCCGGCCA
CGATGCGTCCGGCGTAGAGGATCGAGATCTCGATCCCGCGAAATTAATACGACTCACTATAGGGGAAT
TGTGAGCGGATAACAATTCCCCTCTAGAAATAATTTTGTTTAACTTTAAGAAGGAGATATACCATGGgcgc
gggccatatggctagcatgactggtggacagcaaatgggtcgggatccgaattccatgtccaatttactgaccgtacaccaaaatttgcctgcattaccgg
tcgatgcaacgagtgatgaggttcgcaagaacctgatggacatgttcagggatcgccaggcgttttctgagcatacctggaaaatgcttctgtccgtttgcc
ggtcgtgggcggcatggtgcaagttgaataaccggaaatggtttcccgcagaacctgaagatgttcgcgattatcttctatatcttcaggcgcgcggtctgg
cagtaaaaactatccagcaacatttgggccagctaaacatgcttcatcgtcggtccgggctgccacgaccaagtgacagcaatgctgtttcactggttatg
cggcggatccgaaaagaaaacgttgatgccggtgaacgtgcaaaacaggctctagcgttcgaacgcactgatttcgaccaggttcgttcactcatggaa
aatagcgatcgctgccaggatatacgtaatctggcatttctggggattgcttataacaccctgttacgtatagccgaaattgccaggatcagggttaaagat
atctcacgtactgacggtgggagaatgttaatccatattggcagaacgaaaacgctggttagcaccgcaggtgtagagaaggcacttagcctgggggta
actaaactggtcgagcgatggatttccgtctctggtgtagctgatgatccgaataactacctgttttgccgggtcagaaaaaatggtgttgccgcgccatctg
ccaccagccagctatcaactcgcgccctggaagggatttttgaagcaactcatcgattgatttacggcgctaaggatgactctggtcagagatacctggc
ctggtctggacacagtgcccgtgtcggagccgcgcgagatatggcccgcgctggagtttcaataccggagatcatgcaagctggtggctggaccaatgt
aaatattgtcatgaactatatccgtaacctggatagtgaaacaggggcaatggtgcgcctgctggaagatggcgatgcggccgcaCTCGAGCAC
CACCACCACCACCACTGAGATCCGGCTGCTAACAAAGCCCGAAAGGAAGCTGAGTTGGCTGCTGCCAC
CGCTGAGCAATAACTAGCATAACCCCTTGGGGCCTCTAAACGGGTCTTGAGGGGTTTTTTGCTGAAAGG
AGGAACTATATCCGGAT

>pMXs-mCherry-*rd12*-eGFP-IRES-Blasticidin
CCCGAAAAGTGCCACCTGCATAATGAAAGACCCCACCTGTAGGTTTGGCAAGCTAGCTTAAGTAACGCC
ATTTTGCAAGGCATGGAAAAATACATAACTGAGAATAGAAAAGTTCAGATCAAGGTCAGGAACAGATGG
AACAGCTGAATATGGGCCAAACAGGATATCTGTGGTAAGCAGTTCCTGCCCCGGCTCAGGGCCAAGAA
CAGATGGAACAGCTGAATATGGGCCAAACAGGATATCTGTGGTAAGCAGTTCCTGCCCCGGCTCAGGG
CCAAGAACAGATGGTCCCCAGATGCGGTCCAGCCCTCAGCAGTTTCTAGAGAACCATCAGATGTTTCCA
GGGTGCCCCAAGGACCTGAAATGACCCTGTGCCTTATTTGAACTAACCAATCAGTTCGCTTCTCGCTTC
TGTTCGCGCGCTTCTGCTCCCCGAGCTCAATAAAAGAGCCCACAACCCCTCACTCGGCGCGCCAGTCC
TCCGATTGACTGAGTCGCCCGGGTACCCGTGTATCCAATAAACCCTCTTGCAGTTGCATCCGACTTGTG
GTCTCGCTGTTCCTTGGGAGGGTCTCCTCTGAGTGATTGACTACCCGTCAGCGGGGGTCTTTCATTTGG
GGGCTCGTCCGGGATCGGGAGACCCCTGCCCAGGGACCACCGACCCACCACCGGGAGGTAAGCTGG
CCAGCAACTTATCTGTGTCTGTCCGATTGTCTAGTGTCTATGACTGATTTTATGCGCCTGCGTCGGTACT
AGTTAGCTAACTAGCTCTGTATCTGGCGGACCCGTGGTGGAACTGACGAGTTCGGAACACCCGGCCGC
AACCCTGGGAGACGTCCCAGGGACTTCGGGGGCCGTTTTTGTGGCCCGACCTGAGTCCAAAAATCCCG
ATCGTTTTGGACTCTTTGGTGCACCCCCCTAATAGGAGGGATATGTGGTTCTGGTAGGAGACGAGAACC
TAAAACAGTTCCCGCCTCCGTCTGAATTTTTGCTTTCGGTTTGGGACCGAAGCCGCGCCGCGCGTCTTG
TCTGCTGCAGCATCGTTCTGTGTTGTCTCTGTCTGACTGTGTTTCTGTATTTGTCTGAAAATTAGGGCCA
GACTGTTACCACTCCCTTAAGTTTGACCTTAGGTCACTGGAAAGATGTCGAGCGGATCGCTCACAACCA
GTCGGTAGATGTCAAGAAGAGACGTTGGGTTACCTTCTGCTCTGCAGAATGGCCAACCTTTAACGTCGG
ATGGCCGCGAGACGGCACCTTTAACCGAGACCTCATCACCCAGGTTAAGATCAAGGTCTTTTCACCTGG
CCCGCATGGACACCCAGACCAGGTCCCCTACATCGTGACCTGGGAAGCCTTGGCTTTTGACCCCCCTC
CCTGGGTCAAGCCCTTTGTACACCCTAAGCCTCCGCCTCCTCTTCCTCCATCCGCCCCGTCTCTCCCCC
TTGAACCTCCTCGTTCGACCCCGCCTCGATCCTCCCTTTATCCAGCCCTCACTCCTTCTCTAGGCGCCC
CCATATGGCCATATGAGATCTTATATGGGGCACCCCCGCCCCTTGTAAACTTCCCTGACCCTGACATGA
CAAGAGTTACTAACAGCCCCTCTCTCCAAGCTCACTTACAGGCTCTCTACTTAGTCCAGCACGAAGTCT
GGAGACCTCTGGCGGCAGCCTACCAAGAACAACTGGACCGACCGGTGGTACCTCACCCTTACCGAGTC
GGCGACACAGTGTGGGTCCGCCGACACCAGACTAAGAACCTAGAACCTCGCTGGAAAGGACCTTACAC
AGTCCTGCTGACCACCCCCACCGCCCTCAAAGTAGACGGCATCGCAGCTTGGATACACGCCGCCCACG
TGAAGGCTGCCGACCCCGGGGGTGGACCATCCTCTAGACTGCCGGATCTAGCTAGTTAATTAAGgatcca
ccatggtgagcaagggcgaggaggataacatggccatcatcaaggagttcatgcgcttcaaggtgcacatggagggctccgtgaacggccacgagtt
cgagatcgagggcgagggcgagggccgcccctacgagggcacccagaccgccaagctgaaggtgaccaagggtggcccccctgcccttcgcctgg
gacatcctgtcccctcagttcatgtacggctccaaggcctacgtgaagcaccccgccgacatccccgactacttgaagctgtccttccccgagggcttcaa
gtgggagcgcgtgatgaacttcgaggacggcggcgtggtgaccgtgacccaggactcctccctgcaggacggcgagttcatctacaaggtgaagctg
cgcggcaccaacttcccctccgacggccccgtaatgcagaagaagaccatgggctgggaggcctcctccgagcggatgtaccccgaggacggcgc
cctgaagggcgagatcaagcagaggctgaagctgaaggacggcggccactacgacgctgaggtcaagaccacctacaaggccaagaagcccgt
gcagctgcccggcgcctacaacgtcaacatcaagttggacatcacctcccacaacgaggactacaccatcgtggaacagtacgaacgcgccgagg

gccgccactccaccggcggcatggacgagctgtacaagggctacaagaaactatttgaaactgtggaggaactgtcctcaccactaacagctcatgtc
acaggcaggattcccctctggctcactggcagtctcctctgatgtgggccagggctctttgaagttggatctgagcctttctatcacctgtttgatggacaagc
ccttttgcacaagtttgacttcaaggagggccatGGCAAAGGCGAGGAACTGTTTACAGGCGTGGTGCCAATCCTGGTG
GAACTTGATGGAGATGTGAACGGTCACAAGTTTAGCGTGTCCGGCGAAGGCGAAGGCGACGCCACCTA
CGGCAAGCTGACACTGAAGTTCATCTGCACCACCGGCAAGCTGCCCGTGCCTTGGCCTACCCTGGTTA
CAACACTGACCTACGGCGTCCAATGTTTTAGCAGATACCCCGACCACATGAAAAGACACGACTTCTTCA
AAAGCGCCATGCCAGAGGGCTACGTGCAGGAGCGGACCATCAGCTTCAAGGACGACGGAAACTACAA
GACCAGAGCCGAGGTGAAGTTCGAGGGCGATACACTGGTCAACCGGATCGAGCTGAAGGGCATCGAC
TTCAAAGAGGACGGAAATATCCTGGGCCACAAGCTCGAATACAATTACAACAGCCACAACGTGTACATC
ACAGCTGATAAGCAGAAGAACGGCATTAAGGCCAACTTCAAGATCAGGCATAATATCGAGGATGGATCT
GTGCAGCTGGCCGACCACTATCAGCAGAACACCCCTATCGGCGACGGACCTGTGCTGCTGCCTGACAA
CCACTACCTGAGCACCCAGAGCGCTCTGTCTAAGGACCCCAACGAGAAGAGAGATCACATGGTGCTGC
TGGAATTCGTGACCGCCGCCGGCATCACCCACGgcatggacgagctgtacaagtgacTCGAGGGCCGGCGCGCC
GCGGCCGCTACGTAAATTCCGCCCCCCCCCCTCCCCCCCCCCTAACGTTACTGGCCGAAGCCGCTTG
GAATAAGGCCGGTGTGCGTTTGTCTATATGTTATTTTCCACCATATTGCCGTCTTTTGGCAATGTGAGGG
CCCGGAAACCTGGCCCTGTCTTCTTGACGAGCATTCCTAGGGGTCTTTCCCCTCTCGCCAAAGGAATG
CAAGGTCTGTTGAATGTCGTGAAGGAAGCAGTTCCTCTGGAAGCTTCTTGAAGACAAACAACGTCTGTA
GCGACCCTTTGCAGGCAGCGGAACCCCCCACCTGGCGACAGGTGCCTCTGCGGCCAAAAGCCACGTG
TATAAGATACACCTGCAAAGGCGGCACAACCCCAGTGCCACGTTGTGAGTTGGATAGTTGTGGAAAGA
GTCAAATGGCTCTCCTCAAGCGTATTCAACAAGGGGCTGAAGGATGCCCAGAAGGTACCCCATTGTATG
GGATCTGATCTGGGGCCTCGGTGCACATGCTTTACATGTGTTTAGTCGAGGTTAAAAAAACGTCTAGGC
CCCCCGAACCACGGGGACGTGGTTTTCCTTTGAAAAACACGATGATAATATGGCCACAACCATGGTTCC
TTTGTCTCAAGAAGAATCCACCCTCATTGAAAGAGCAACGGCTACAATCAACAGCATCCCCATCTCTGAA
GACTACAGCGTCGCCAGCGCAGCTCTCTCTAGCGACGGCCGCATCTTCACTGGTGTCAATGTATATCAT
TTTACTGGGGGACCTTGTGCAGAACTCGTGGTGCTGGGCACTGCTGCTGCTGCGGCAGCTGGCAACCT
GACTTGTATCGTCGCGATCGGAAATGAGAACAGGGGCATCTTGAGCCCCTGCGGACGGTGCCGACAG
GTGCTTCTCGATCTGCATCCTGGGATCAAAGCCATAGTGAAGGACAGTGATGGACAGCCGACGGCAGT
TGGGATTCGTGAATTGCTGCCCTCTGGTTATGTGTGGGAGGGCTAAGTCGACGATAAAATAAAAGATTT
TATTTAGTCTCCAGAAAAAGGGGGGAATGAAAGACCCCACCTGTAGGTTTGGCAAGCTAGCTTAAGTAA
CGCCATTTTGCAAGGCATGGAAAAATACATAACTGAGAATAGAGAAGTTCAGATCAAGGTCAGGAACAG
ATGGAACAGCTGAATATGGGCCAAACAGGATATCTGTGGTAAGCAGTTCCTGCCCCGGCTCAGGGCCA
AGAACAGATGGAACAGCTGAATATGGGCCAAACAGGATATCTGTGGTAAGCAGTTCCTGCCCCGGCTC
AGGGCCAAGAACAGATGGTCCCCAGATGCGGTCCAGCCCTCAGCAGTTTCTAGAGAACCATCAGATGT
TTCCAGGGTGCCCCAAGGACCTGAAATGACCCTGTGCCTTATTTGAACTAACCAATCAGTTCGCTTCTC
GCTTCTGTTCGCGCGCTTCTGCTCCCCGAGCTCAATAAAAGAGCCCACAACCCCTCACTCGGGGCGCC
AGTCCTCCGATTGACTGAGTCGCCCGGGTACCCGTGTATCCAATAAACCCTCTTGCAGTTGCATCCGAC
TTGTGGTCTCGCTGTTCCTTGGGAGGGTCTCCTCTGAGTGATTGACTACCCGTCAGCGGGGGTCTTTCA
CATGCAGCATGTATCAAAATTAATTTGGTTTTTTTTCTTAAGTATTTACATTAAATGGCCATAGTTGCATTA
ATGAATCGGCCAACGCGCGGGGAGAGGCGGTTTGCGTATTGGGCGCTCTTCCGCTTCCTCGCTCACTG
ACTCGCTGCGCTCGGTCGTTCGGCTGCGGCGAGCGGTATCAGCTCACTCAAAGGCGGTAATACGGTTA
TCCACAGAATCAGGGGATAACGCAGGAAAGAACATGTGAGCAAAAGGCCAGCAAAAGGCCAGGAACCG
TAAAAAGGCCGCGTTGCTGGCGTTTTTCCATAGGCTCCGCCCCCCTGACGAGCATCACAAAAATCGAC
GCTCAAGTCAGAGGTGGCGAAACCCGACAGGACTATAAAGATACCAGGCGTTTCCCCCTGGAAGCTCC
CTCGTGCGCTCTCCTGTTCCGACCCTGCCGCTTACCGGATACCTGTCCGCCTTTCTCCCTTCGGGAAG
CGTGGCGCTTTCTCATAGCTCACGCTGTAGGTATCTCAGTTCGGTGTAGGTCGTTCGCTCCAAGCTGG
GCTGTGTGCACGAACCCCCCGTTCAGCCCGACCGCTGCGCCTTATCCGGTAACTATCGTCTTGAGTCC
AACCCGGTAAGACACGACTTATCGCCACTGGCAGCAGCCACTGGTAACAGGATTAGCAGAGCGAGGTA
TGTAGGCGGTGCTACAGAGTTCTTGAAGTGGTGGCCTAACTACGGCTACACTAGAAGAACAGTATTTGG
TATCTGCGCTCTGCTGAAGCCAGTTACCTTCGGAAAAAGAGTTGGTAGCTCTTGATCCGGCAAACAAAC
CACCGCTGGTAGCGGTGGTTTTTTTGTTTGCAAGCAGCAGATTACGCGCAGAAAAAAAGGATCTCAAGA
AGATCCTTTGATCTTTTCTACGGGGTCTGACGCTCAGTGGAACGAAAACTCACGTTAAGGGATTTTGGT
CATGAGATTATCAAAAAGGATCTTCACCTAGATCCTTTTGCGGCCGGCCGCAAATCAATCTAAAGTATAT
ATGAGTAAACTTGGTCTGACAGTTACCAATGCTTAATCAGTGAGGCACCTATCTCAGCGATCTGTCTATT
TCGTTCATCCATAGTTGCCTGACTCCCCGTCGTGTAGATAACTACGATACGGGAGGGCTTACCATCTGG
CCCCAGTGCTGCAATGATACCGCGAGACCCACGCTCACCGGCTCCAGATTTATCAGCAATAAACCAGC
CAGCCGGAAGGGCCGAGCGCAGAAGTGGTCCTGCAACTTTATCCGCCTCCATCCAGTCTATTAATTGTT
GCCGGGAAGCTAGAGTAAGTAGTTCGCCAGTTAATAGTTTGCGCAACGTTGTTGCCATTGCTACAGGCA
TCGTGGTGTCACGCTCGTCGTTTGGTATGGCTTCATTCAGCTCCGGTTCCCAACGATCAAGGCGAGTTA
CATGATCCCCCATGTTGTGCAAAAAAGCGGTTAGCTCCTTCGGTCCTCCGATCGTTGTCAGAAGTAAGT

TGGCCGCAGTGTTATCACTCATGGTTATGGCAGCACTGCATAATTCTCTTACTGTCATGCCATCCGTAAG
ATGCTTTTCTGTGACTGGTGAGTACTCAACCAAGTCATTCTGAGAATAGTGTATGCGGCGACCGAGTTG
CTCTTGCCCGGCGTCAATACGGGATAATACCGCGCCACATAGCAGAACTTTAAAAGTGCTCATCATTGG
AAAACGTTCTTCGGGGCGAAAACTCTCAAGGATCTTACCGCTGTTGAGATCCAGTTCGATGTAACCCAC
TCGTGCACCCAACTGATCTTCAGCATCTTTTACTTTCACCAGCGTTTCTGGGTGAGCAAAAACAGGAAG
GCAAAATGCCGCAAAAAAGGGAATAAGGGCGACACGGAAATGTTGAATACTCATACTCTTCCTTTTTCAA
TATTATTGAAGCATTTATCAGGGTTATTGTCTCATGAGCGGATACATATTTGAATGTATTTAGAAAAATAA
ACAAATAGGGGTTCCGCGCACATTTC

>pRha-ABE8e-SpCas9-NG-TEV-1D4
agctcctgaaaatctcgataactcaaaaaatacgcccggtagtgatcttatttcattatggtgaaagttggaacctcttacgtgccgatcaagaagacggtc
aaaagcctccggtcggaggcttttgactttctgctatggaggtcaggtatgatttaaatggtcagtattgagcgatatctagagaattcgtccaccacaattca
gcaaattgtgaacatcatcacgttcatctttccctggttgccaatggcccattttcctgtcagtaacgagaaggtcgcgaattcaggcgctttttagactggtcg
taatgaaattctttttaagaaggagatatacatatgcaccatcaccatcatcatcatcacaaacgtaccgcagatggtagcgaatttgaaagcccgaaaa
aaaagcgtaaagttagcgaagttgaatttagccacgaatactggatgcgtcatgcgttgacgctggcgaagcgtgcgcgtgacgaacgcgaggtgccg
gtgggtgctgttctggtgctgaacaatcgtgtaatcggtgaaggttggaatcgcgcgatcggtctgcatgatccaaccgcgcacgcagaaatcatggcgc
tgcgtcagggtggcctggtgatgcaaaattaccgtctgatcgatgcgacgctgtatgtgaccttcgaaccgtgtgtcatgtgtgccggtgccatgatccaca
gccgcattggccgtgtggtctttggcgttcgtaacagcaaacgtggcgcagcaggctctctgatgaacgtcctgaattacccgggtatgaaccatcgtgttg
agattacggagggcatcttagcggatgaatgtgctgcgctgctgtgcgatttctatcgcatgccgcgtcaagtctttaacgcacagaagaaggcacaatct
agcattaactctggcggttcgtctggtggtagcagcggcagcgaaacgccgggtaccagcgagagcgcaaccccggaaagctccggtggcagctct
ggcggctctgacaaaaagtattcgattggcctggcgatcggcacgaacagcgtcggttgggctgttattaccgacgagtataaagtgccgagcaagaa
gtttaaagtcttgggtaacaccgatcgccatagcatcaaaaagaatctgattggtgccctgctgtttgatagcggcgaaacggcagaagcgacccgtctg
aaacgtaccgcacgtcgtcgctacactcgtcgtaaaaaccgcatttgctatttgcaagagatcttcagcaatgaaatggccaaagttgacgacagcttcttt
caccgtctggaggagagctttctggttgaagaggataagaaacacgagcgtcacccgatctttggtaatatcgttgacgaggtcgcgtatcacgagaagt
acccgacgatctaccacttgcgtaagaaactggtggacagcaccgataaagccgatctgcgtctgatttacttggcgttggcgcacatgatcaagttccg
cggtcatttcctgatcgagggtgacctgaacccagataattccgacgtcgacaagctgttcatccagctggttcagacctacaaccaattgtttgaagaaa
acccgattaatgcatctggtgtcgacgcgaaggcgattctgagcgcgcgtctgagcaagtctcgtcgtctggagaatctgatcgcgcaattgccgggtga
gaagaaaaatggtttgtttggcaacttgatcgcgctgagcctgggtctgaccccgaactttaagagcaatttcgacctggcagaagatgcgaaactgcag
ctgagcaaagacacctatgatgacgacctggataatctcctggcacaaattggtgaccagtatgcggacctgttcctggctgcgaaaaatctgtcggacg
ccattctgctgtcggacattctgcgcgttaacactgagatcacgaaagcaccgctgtctgcgagcatgattaaacgttatgacgagcaccatcaagacct
gaccctgctgaaggccctggtccgtcagcaactgccggagaagtataaagaaatttttttcgatcagagcaagaacggttacgcaggctatatcgatgg
cggtgcgagccaagaagagttttacaaattcattaaaccgattttggagaagatggacggtaccgaggagttgctggtgaaactgaatcgcgaagatct
gctgcgcaaacaacgtacgttcgataacggtagcatcccacaccagattcacctgggtgagctgcacgcgattctgcgtcgtcaggaagatttctaccca
ttcctgaaagataatcgtgaaaagattgagaaaatcttgacgttccgcattccgtactacgtcggtccgctggcacgtggcaacagccgtttcgcttggatg
acgcgcaagagcgaggaaactatcacgccgtggaactttgaggaagtcgtcgacaaaggtgcgagcgcgcaaagctttattgagcgcatgaccaac
tttgacaagaatctgccgaacgagaaagtcttgccaaagcacagcctgctgtacgagtatttcacggtgtacaatgaactgaccaaagttaagtatgtca
ccgagggtatgcgtaaaccagcgttcctgtccggtgaacagaagaaagcaattgtggatctgttgtttaagacgaaccgtaaagtcacggtgaagcagtt
gaaagaggattacttcaaaaagatcgaatgctttgacagcgtggaaattagcggtgttgaggaccgtttcaacgccagcttgggcacctaccatgatctg
ctgaaaatcattaaggacaaggacttcctggataacgaagaaaacgaggatattctggaggatattgtcctgacgctgaccctcttcgaagatcgcgaa
atgattgaagagagactgaaaacttacgcccacttgtttgacgataaagtcatgaaacaactgaagcgccgtcgttacaccggttggggtcgtctgagcc
gtaagctgatcaacggtatccgtgataagcaatctggcaaaacgatcctggactttctgaaaagcgatggtttcgcgaaccgcaacttcatgcagcttattc
acgacgattctctgacctttaaggaggacatccaaaaagcccaggttagcggtcagggcgattccctgcatgagcatattgctaacttagcgggtagccc
ggcaatcaaaaagggcattctgcaaaccgttaaggttgtggacgagttggttaaggtcatgggccgtcataagccggagaatatcgttattgagatggcg
cgcgagaatcagaccacgcagaaaggccaaaagaatagccgtgaacgtatgaaacgtattgaagagggtatcaaagagctgggcagccaaatctt
gaaggaacatccggtggagaacacgcagctgcagaatgaaaagctgtacctgtactatctgcaaaacggtcgtgatatgtacgttgaccaagagctgg
acatcaaccgcctgagcgattatgatgttgatcacattgtgccgcaatcttttctgaaggacgacagcatcgacaacaaagttttgacccgtagcgacaag
aatcgcggtaaaagcgataatgttccgagcgaagaggtcgttaagaagatgaaaaactattggcgtcagttgctgaatgcaaaactgattacccaacg
caaattcgacaatttgactaaagcggagcgtggtggtctgagcgagctggacaaggcgggtttcattaagcgtcaactggttgaaactcgtcagatcacg
aagcacgtggcgcagattctggacagccgcatgaatactaagtacgatgaaaatgataaactcattcgtgaggtcaaggtcattaccctgaagtctaag
ctcgtcagcgattttcgtaaagacttccagttctataaggtgcgcgaaatcaacaattaccaccacgcccatgatgcatatctgaacgccgtggtgggtac
ggctctgatcaagaaatatccgaaactggagtccgagtttgtctatggtgactacaaagtgtatgacgtccgcaaaatgatcgccaaatcggaacaaga
gattggcaaggcaaccgcgaagtactttttctattccaacattatgaactttttcaagaccgagatcaccctggcaaacggtgagatccgtaaacgtccgct
gatcgaaactaacggtgaaaccggtgaaatcgtgtgggacaaaggtcgtgactttgcaaccgtgcgtaaagtgctgtcgatgccacaggtcaacatcgt
taagaaaaccgaagttcaaactggtggctttagcaaggagagcattcgtcctaagcgcaacagcgacaagctgattgcacgtaagaaggactgggac
ccgaagaagtacggcggctttgtgtccccgacggttgcgtatagcgttctcgttgtggcgaaagtcgagaaaggcaagagcaaaaagctgaaaagcgt
taaagaattgctgggtatcacgattatggagcgtagcagcttcgaaaagaatcctattgatttcttggaagcaaaaggttacaaggaggtgaagaaaga
cttgatcatcaaactgccgaagtattccctgttcgagctggaaaacggccgcaaacgtatgctggccagcgctcgctttctgcaaaagggcaacgaact
ggctctgccgagcaaatatgtgaatttcctgtacctggccagccactacgagaaactgaagggctccccggaagataacgagcagaagcagctgttcg
ttgagcagcacaagcattacctggacgagattatcgagcagatcagcgagtttagcaaacgcgtgattctggcggatgcgaatttggataaagttctgtcg

gcctataataaacaccgcgacaaaccgattcgcgagcaggcggagaacattatccacttgttcaccctgaccaatctgggtgctccgcgtgctttcaaat
actttgataccacgattgatcgcaaagtgtatcgttccaccaaagaggtactggacgcgaccctgatccaccagagcatcaccggcttgtacgaaacgc
gcatcgatctgtctcaactgggcggtgacagcggtggttcgaaacgtaccgcggatggctctgaatttgaatctccgaagaagaaacgcaaagtcGAA
AATCTATATTTTCAATCAACTGAGACAAGCCAGGTGGCTCCGGCGtaataaggttagagcggccgccaccgctgagcaa
taactagcataaccccttggggcctctaaacgggtcttgaggggttttttgctgaaaggaggaactatatccgggtaacgaattcaagcttgatatcattcag
gacgagcctcagactccagcgtaactggactgcaatcaactcactggctcaccttcacgggtgggcctttcttcggtagaagtcttcttaataagatgatctt
cttgagatcgttttggtctgcgcgtaatctcttgctctgaaaacgaaaaaaccgccttgcagggcggtttttcgaaggttctctgagctaccaactctttgaacc
gaggtaactggcttggaggagcgcagtcaccaaaacttgtcctttcagtttagccttaaccggcgcatgacttcaagactaactcctctaaatcaattacca
gtggctgctgccagtggtgcttttgcatgtctttccgggttggactcaagacgatagttaccggataaggcgcagcggtcggactgaacggggggttcgtg
catacagtccagcttggagcgaactgcctacccggaactgagtgtcaggcgtggaatgagacaaacgcggccataacagcggaatgacaccggtaa
accgaaaggcaggaacaggagagcgcacgagggagccgccaggggggaaacgcctggtatctttatagtcctgtcgggtttcgccaccactgatttga
gcgtcagatttcgtgatgcttgtcaggggggcggagcctatggaaaaacggctttgccgcggccctctcacttccctgttaagtatcttcctggcatcttccag
gaaatctccgccccgttcgtaagccatttccgctcgccgcagtcgaacgaccgagcgtagcgagtcagtgagcgaggaagcggaatatatcctgtatca
catattctgctgacgcaccggtgcagcctttttttctcctgccacatgaagcacttcactgacaccctcatcagtgccaacatagtaagccagtatacactccg
ctagcgcagaaaggcccacccgaaggtgagccaggtgattacatttgggccctcattagaaaaactcatcgagcatcaaatgaaattgcaatttattcat
atcaggattatcaataccatatttttgaaaaagccgtttctgtaatgaaggagaaaactcaccgaggcagttccataggatggcaagatcctggtatcggtc
tgcgattccgactcgtccaacatcaatacaacctattaatttcccctcgtcaaaaataaggttatcaagtgagaaatcaccatgagtgacgactgaatccg
gtgagaatggcaaaagtttatgcatttctttccagacttgttcaacaggccagccattacgctcgtcatcaaaatcactcgcatcaaccaaaccgttattcatt
cgtgattgcgcctgagcgaggcgaaatacgcgatcgctgttaaaaggacaattacaaacaggaatcgagtgcaaccggcgcaggaacactgccagc
gcatcaacaatattttcacctgaatcaggatattcttctaatacctggaacgctgtttttccggggatcgcagtggtgagtaaccatgcatcatcaggagtac
ggataaaatgcttgatggtcggaagtggcataaattccgtcagccagtttagtctgaccatctcatctgtaacatcattggcaacgctacctttgccatgtttca
gaaacaactctggcgcatcgggcttcccatacaagcgatagattgtcgcacctgattgcccgacattatcgcgagcccatttatacccatataaatcagca
tccatgttggaatttaatcgcggcctcgacgtttcccgttgaatatggctcat

>pRha-PE2-TEV-1D4
agctcctgaaaatctcgataactcaaaaaatacgcccggtagtgatcttatttcattatggtgaaagttggaacctcttacgtgccgatcaagaagacggtc
aaaagcctccggtcggaggcttttgactttctgctatggaggtcaggtatgatttaaatggtcagtattgagcgatatctagagaattcgtccaccacaattca
gcaaattgtgaacatcatcacgttcatctttccctggttgccaatggcccattttcctgtcagtaacgagaaggtcgcgaattcaggcgctttttagactggtcg
taatgaaattctttttaagaaggagatatacatatgcaccatcaccatcatcatcatcacccaaagaagaagcggaaagtcgacaagaagtacagcatc
ggcctggacatcggcaccaactctgtgggctgggccgtgatcaccgacgagtacaaggtgcccagcaagaaattcaaggtgctgggcaacaccgac
cggcacagcatcaagaagaacctgatcggagccctgctgttcgacagcggcgaaacagccgaggccacccggctgaagagaaccgccagaaga
agatacaccagacggaagaaccggatctgctatctgcaagagatcttcagcaacgagatggccaaggtggacgacagcttcttccacagactggaag
agtccttcctggtggaagaggataagaagcacgagcggcaccccatcttcggcaacatcgtggacgaggtggcctaccacgagaagtaccccaccat
ctaccacctgagaaagaaactggtggacagcaccgacaaggccgacctgcggctgatctatctggccctggcccacatgatcaagttccggggccac
ttcctgatcgagggcgacctgaaccccgacaacagcgacgtggacaagctgttcatccagctggtgcagacctacaaccagctgttcgaggaaaacc
ccatcaacgccagcggcgtggacgccaaggccatcctgtctgccagactgagcaagagcagacggctggaaaatctgatcgcccagctgcccggc
gagaagaagaatggcctgttcggaaacctgattgccctgagcctgggcctgacccccaacttcaagagcaacttcgacctggccgaggatgccaaact
gcagctgagcaaggacacctacgacgacgacctggacaacctgctggcccagatcggcgaccagtacgccgacctgtttctggccgccaagaacct
gtccgacgccatcctgctgagcgacatcctgagagtgaacaccgagatcaccaaggcccccctgagcgcctctatgatcaagagatacgacgagca
ccaccaggacctgaccctgctgaaagctctcgtgcggcagcagctgcctgagaagtacaaagagattttcttcgaccagagcaagaacggctacgcc
ggctacattgacggcggagccagccaggaagagttctacaagttcatcaagcccatcctggaaaagatggacggcaccgaggaactgctcgtgaag
ctgaacagagaggacctgctgcggaagcagcggaccttcgacaacggcagcatcccccaccagatccacctgggagagctgcacgccattctgcg
gcggcaggaagatttttacccattcctgaaggacaaccgggaaaagatcgagaagatcctgaccttccgcatcccctactacgtgggccctctggccag
gggaaacagcagattcgcctggatgaccagaaagagcgaggaaaccatcaccccctggaacttcgaggaagtggtggacaagggcgcttccgccc
agagcttcatcgagcggatgaccaacttcgataagaacctgcccaacgagaaggtgctgcccaagcacagcctgctgtacgagtacttcaccgtgtat
aacgagctgaccaaagtgaaatacgtgaccgagggaatgagaaagcccgccttcctgagcggcgagcagaaaaaggccatcgtggacctgctgttc
aagaccaaccggaaagtgaccgtgaagcagctgaaagaggactacttcaagaaaatcgagtgcttcgactccgtggaaatctccggcgtggaagat
cggttcaacgcctccctgggcacataccacgatctgctgaaaattatcaaggacaaggacttcctggacaatgaggaaaacgaggacattctggaaga
tatcgtgctgaccctgacactgtttgaggacagagagatgatcgaggaacggctgaaaacctatgcccacctgttcgacgacaaagtgatgaagcagc
tgaagcggcggagatacaccggctggggcaggctgagccggaagctgatcaacggcatccgggacaagcagtccggcaagacaatcctggatttc
ctgaagtccgacggcttcgccaacagaaacttcatgcagctgatccacgacgacagcctgacctttaaagaggacatccagaaagcccaggtgtccg
gccagggcgatagcctgcacgagcacattgccaatctggccggcagccccgccattaagaagggcatcctgcagacagtgaaggtggtggacgag
ctcgtgaaagtgatgggccggcacaagcccgagaacatcgtgatcgaaatggccagagagaaccagaccacccagaagggacagaagaacagc
cgcgagagaatgaagcggatcgaagagggcatcaaagagctgggcagccagatcctgaaagaacaccccgtggaaaacacccagctgcagaac
gagaagctgtacctgtactacctgcagaatgggcgggatatgtacgtggaccaggaactggacatcaaccggctgtccgactacgatgtggacgctatc
gtgcctcagagctttctgaaggacgactccatcgacaacaaggtgctgaccagaagcgacaagaaccggggcaagagcgacaacgtgccctccga
agaggtcgtgaagaagatgaagaactactggcggcagctgctgaacgccaagctgattacccagagaaagttcgacaatctgaccaaggccgaga
gaggcggcctgagcgaactggataaggccggcttcatcaagagacagctggtggaaacccggcagatcacaaagcacgtggcacagatcctggac
tcccggatgaacactaagtacgacgagaatgacaagctgatccgggaagtgaaagtgatcaccctgaagtccaagctggtgtccgatttccggaagg

atttccagttttacaaagtgcgcgagatcaacaactaccaccacgcccacgacgcctacctgaacgccgtcgtgggaaccgccctgatcaaaaagtac
cctaagctggaaagcgagttcgtgtacggcgactacaaggtgtacgacgtgcggaagatgatcgccaagagcgagcaggaaatcggcaaggctac
cgccaagtacttcttctacagcaacatcatgaactttttcaagaccgagattaccctggccaacggcgagatccggaagcggcctctgatcgagacaaa
cggcgaaaccggggagatcgtgtgggataagggccgggattttgccaccgtgcggaaagtgctgagcatgccccaagtgaatatcgtgaaaaagac
cgaggtgcagacaggcggcttcagcaaagagtctatcctgcccaagaggaacagcgataagctgatcgccagaaagaaggactgggaccctaag
aagtacggcggcttcgacagccccaccgtggcctattctgtgctggtggtggccaaagtggaaaagggcaagtccaagaaactgaagagtgtgaaag
agctgctggggatcaccatcatggaaagaagcagcttcgagaagaatcccatcgactttctggaagccaagggctacaaagaagtgaaaaaggacc
tgatcatcaagctgcctaagtactccctgttcgagctggaaaacggccggaagagaatgctggcctctgccggcgaactgcagaagggaaacgaact
ggccctgccctccaaatatgtgaacttcctgtacctggccagccactatgagaagctgaagggctcccccgaggataatgagcagaaacagctgtttgt
ggaacagcacaagcactacctggacgagatcatcgagcagatcagcgagttctccaagagagtgatcctggccgacgctaatctggacaaagtgctg
tccgcctacaacaagcaccgggataagcccatcagagagcaggccgagaatatcatccacctgtttaccctgaccaatctgggagcccctgccgcctt
caagtactttgacaccaccatcgaccggaagaggtacaccagcaccaaagaggtgctggacgccaccctgatccaccagagcatcaccggcctgta
cgagacacggatcgacctgtctcagctgggaggtgactctggaggatctagcggaggatcctctggcagcgagacaccaggaacaagcgagtcagc
aacaccagagagcagtggcggcagcagcggcggcagcagcaccctaaatatagaagatgagtatcggctacatgagacctcaaaagagccagat
gtttctctagggtccacatggctgtctgattttcctcaggcctgggcggaaaccgggggcatgggactggcagttcgccaagctcctctgatcatacctctga
aagcaacctctacccccgtgtccataaaacaataccccatgtcacaagaagccagactggggatcaagccccacatacagagactgttggaccagg
gaatactggtaccctgccagtcccctggaacacgcccctgctacccgttaagaaaccagggactaatgattataggcctgtccaggatctgagagaag
tcaacaagcgggtggaagacatccaccccaccgtgcccaacccttacaacctcttgagcgggctcccaccgtcccaccagtggtacactgtgcttgattt
aaaggatgcctttttctgcctgagactccaccccaccagtcagcctctcttcgcctttgagtggagagatccagagatgggaatctcaggacaattgacctg
gaccagactcccacagggtttcaaaaacagtcccaccctgtttaatgaggcactgcacagagacctagcagacttccggatccagcacccagacttga
tcctgctacagtacgtggatgacttactgctggccgccacttctgagctagactgccaacaaggtactcgggccctgttacaaaccctagggaacctcgg
gtatcgggcctcggccaagaaagcccaaatttgccagaaacaggtcaagtatctggggtatcttctaaaagagggtcagagatggctgactgaggcca
gaaaagagactgtgatggggcagcctactccgaagacccctcgacaactaagggagttcctagggaaggcaggcttctgtcgcctcttcatccctgggt
ttgcagaaatggcagccccccctgtaccctctcaccaaaccggggactctgtttaattggggcccagaccaacaaaaggcctatcaagaaatcaagcaa
gctcttctaactgccccagccctggggttgccagatttgactaagccctttgaactctttgtcgacgagaagcagggctacgccaaaggtgtcctaacgca
aaaactgggaccttggcgtcggccggtggcctacctgtccaaaaagctagacccagtagcagctgggtggcccccttgcctacggatggtagcagcca
ttgccgtactgacaaaggatgcaggcaagctaaccatgggacagccactagtcattctggccccccatgcagtagaggcactagtcaaacaacccccc
cgaccgctggctttccaacgcccggatgactcactatcaggccttgcttttggacacggaccgggtccagttcggaccggtggtagccctgaacccggct
acgctgctcccactgcctgaggaagggctgcaacacaactgccttgatatcctggccgaagcccacggaacccgacccgacctaacggaccagccg
ctcccagacgccgaccacacctggtacacggatggaagcagtctcttacaagagggacagcgtaaggcgggagctgcggtgaccaccgagaccga
ggtaatctgggctaaagccctgccagccgggacatccgctcagcgggctgaactgatagcactcacccaggccctaaagatggcagaaggtaagaa
gctaaatgtttatactgatagccgttatgcttttgctactgcccatatccatggagaaatatacagaaggcgtgggtggctcacatcagaaggcaaagagat
caaaaataaagacgagatcttggccctactaaaagccctctttctgcccaaaagacttagcataatccattgtccaggacatcaaaagggacacagcg
ccgaggctagaggcaaccggatggctgaccaagcggcccgaaaggcagccatcacagagactccagacacctctaccctcctcatagaaaattcat
caccctctggcggctcaaaaagaaccgccgacggcagcgaattcgagcccaagaagaagaggaaagtcGAAAATCTATATTTTCAAT
CAACTGAGACAAGCCAGGTGGCTCCGGCGtaagcggccgccaccgctgagcaataactagcataaccccttggggcctctaaac
gggtcttgaggggttttttgctgaaaggaggaactatatccgggtaacgaattcaagcttgatatcattcaggacgagcctcagactccagcgtaactgga
ctgcaatcaactcactggctcaccttcacgggtgggcctttcttcggtagaagtcttcttaataagatgatcttcttgagatcgttttggtctgcgcgtaatctcttg
ctctgaaaacgaaaaaaccgccttgcagggcggtttttcgaaggttctctgagctaccaactctttgaaccgaggtaactggcttggaggagcgcagtca
ccaaaacttgtcctttcagtttagccttaaccggcgcatgacttcaagactaactcctctaaatcaattaccagtggctgctgccagtggtgcttttgcatgtcttt
ccgggttggactcaagacgatagttaccggataaggcgcagcggtcggactgaacggggggttcgtgcatacagtccagcttggagcgaactgcctac
ccggaactgagtgtcaggcgtggaatgagacaaacgcggccataacagcggaatgacaccggtaaaccgaaaggcaggaacaggagagcgca
cgagggagccgccagggggaaacgcctggtatctttatagtcctgtcgggtttcgccaccactgatttgagcgtcagatttcgtgatgcttgtcaggggggc
ggagcctatggaaaaacggctttgccgcggccctctcacttccctgttaagtatcttcctggcatcttccaggaaatctccgccccgttcgtaagccatttccg
ctcgccgcagtcgaacgaccgagcgtagcgagtcagtgagcgaggaagcggaatatatcctgtatcacatattctgctgacgcaccggtgcagcctttttt
tctcctgccacatgaagcacttcactgacaccctcatcagtgccaacatagtaagccagtatacactccgctagcgcagaaaggcccacccgaaggtg
agccaggtgattacatttgggccctcattagaaaaactcatcgagcatcaaatgaaattgcaatttattcatatcaggattatcaataccatatttttgaaaaa
gccgtttctgtaatgaaggagaaaactcaccgaggcagttccataggatggcaagatcctggtatcggtctgcgattccgactcgtccaacatcaataca
acctattaatttcccctcgtcaaaaataaggttatcaagtgagaaatcaccatgagtgacgactgaatccggtgagaatggcaaaagtttatgcatttctttc
cagacttgttcaacaggccagccattacgctcgtcatcaaaatcactcgcatcaaccaaaccgttattcattcgtgattgcgcctgagcgaggcgaaatac
gcgatcgctgttaaaaggacaattacaaacaggaatcgagtgcaaccggcgcaggaacactgccagcgcatcaacaatattttcacctgaatcaggat
attcttctaatacctggaacgctgtttttccggggatcgcagtggtgagtaaccatgcatcatcaggagtacggataaaatgcttgatggtcggaagtggcat
aaattccgtcagccagtttagtctgaccatctcatctgtaacatcattggcaacgctacctttgccatgtttcagaaacaactctggcgcatcgggcttcccat
acaagcgatagattgtcgcacctgattgcccgacattatcgcgagcccatttatacccatataaatcagcatccatgttggaatttaatcgcggcctcgacg
tttcccgttgaatatggctcat

>pCMV-MMLVgag-3xNES-Cre
atgggccagactgttaccactcccttaagtttgaccttaggtcactggaaagatgtcgagcggatcgctcacaaccagtcggtagatgtcaagaagagac
gttgggttaccttctgctctgcagaatggccaacctttaacgtcggatggccgcgagacggcacctttaaccgagacctcatcacccaggttaagatcaag

gtcttttcacctggcccgcatggacacccagaccaggtcccctacatcgtgacctgggaagccttggcttttgacccccctccctgggtcaagccctttgtac
accctaagcctccgcctcctcttcctccatccgccccgtctctcccccttgaacctcctcgttcgaccccgcctcgatcctccctttatccagccctcactccttc
tctaggcgccaaacctaaacctcaagttctttctgacagtgggggggccgctcatcgacctacttacagaagacccccgccttatagggacccaagacc
accccccttccgacagggacggaaatggtggagaagcgacccctgcgggagaggcaccggacccctccccaatggcatctcgcctacgtgggagac
gggagcccccctgtggccgactccactacctcgcaggcattcccctccgcgcaggaggaaacggacagcttcaatactggccgttctcctcttctgacctt
tacaactggaaaaataataacccttctttttctgaagatccaggtaaactgacagctctgatcgagtctgtcctcatcacccatcagcccacctgggacgac
tgtcagcagctgttggggactctgctgaccggagaagaaaaacaacgggtgctcttagaggctagaaaggcggtgcggggcgatgatgggcgcccc
actcaactgcccaatgaagtcgatgccgcttttcccctcgagcgcccagactgggattacaccacccaggcaggtaggaaccacctagtccactatcgc
cagttgctcctagcgggtctccaaaacgcgggcagaagccccaccaatttggccaaggtaaaaggaataacacaagggcccaatgagtctccctcgg
ccttcctagagagacttaaggaagcctatcgcaggtacactccttatgaccctgaggacccagggcaagaaactaatgtgtctatgtctttcatttggcagt
ctgccccagacattgggagaaagttagagaggttagaagatttaaaaaacaagacgcttggagatttggttagagaggcagaaaagatctttaataaa
cgagaaaccccggaagaaagagaggaacgtatcaggagagaaacagaggaaaaagaagaacgccgtaggacagaggatgagcagaaaga
gaaagaaagagatcgtaggagacatagagagatgagcaagctattggccactgtcgttagtggacagaaacaggatagacagggaggagaacga
aggaggtcccaactcgatcgcgaccagtgtgcctactgcaaagaaaaggggcactgggctaaagattgtcccaagaaaccacgaggacctcgggg
accaagaccccagacctccctcctgaccctagatgactctggcggctcacttcaactgcctccacttgaaagactgacactgggatcattacaattacctc
ctttagaacgattaacactcggttcactacagcttccgcctcttgagagattgacattaacgtccacgctgctaatggagaactcgtctggagattacaaag
acgatgacgataagaaacggacagccgacggaagcgagttcgagtcaccaaagaagaagcggaaagtcagcaatttactgaccgtacaccaaaa
tttgcctgcattaccggtcgatgcaacgagtgatgaggttcgcaagaacctgatggacatgttcagggatcgccaggcgttttctgagcatacctggaaaa
tgcttctgtccgtttgccggtcgtgggcggcatggtgcaagttgaataaccggaaatggtttcccgcagaacctgaagatgttcgcgattatcttctatatcttc
aggcgcgcggtctggcagtaaaaactatccagcaacatttgggccagctaaacatgcttcatcgtcggtccgggctgccacgaccaagtgacagcaat
gctgtttcactggttatgcggcggatccgaaaagaaaacgttgatgccggtgaacgtgcaaaacaggctctagcgttcgaacgcactgatttcgaccag
gttcgttcactcatggaaaatagcgatcgctgccaggatatacgtaatctggcatttctggggattgcttataacaccctgttacgtatagccgaaattgcca
ggatcagggttaaagatatctcacgtactgacggtgggagaatgttaatccatattggcagaacgaaaacgctggttagcaccgcaggtgtagagaag
gcacttagcctgggggtaactaaactggtcgagcgatggatttccgtctctggtgtagctgatgatccgaataactacctgttttgccgggtcagaaaaaat
ggtgttgccgcgccatctgccaccagccagctatcaactcgcgccctggaagggatttttgaagcaactcatcgattgatttacggcgctaaggatgactct
ggtcagagatacctggcctggtctggacacagtgcccgtgtcggagccgcgcgagatatggcccgcgctggagtttcaataccggagatcatgcaagc
tggtggctggaccaatgtaaatattgtcatgaactatatccgtaacctggatagtgaaacaggggcaatggtgcgcctgctggaagatggcgactaaatc
tttttccctctgccaaaaattatggggacatcatgaagccccttgagcatctgacttctggctaataaaggaaatttattttcattgcaatagtgtgttggaattttttt
gtgtctctcactcggaaggacatatgggagggcaaatcatttaaaacatcagaatgagtatttggtttagagtttggcaacatatgcccatatgctggctgcc
atgaacaaaggttggctataaagaggtcatcagtatatgaaacagccccctgctgtccattccttattccatagaaaagccttgacttgaggttagatttttttt
atattttgttttgtgttattttttttctttaacatccctaaaattttccttacatgttttactagccagatttttcctcctctcctgactactcccagtcatagctgtccctcttctc
ttatggagatccctcgacggatcggccgcaattcgtaatcatgtcatagctgtttcctgtgtgaaattgttatccgctcacaattccacacaacatacgagcc
ggaagcataaagtgtaaagcctggggtgcctaatgagtgagctaactcacattaattgcgttgcgctcactgcccgctttccagtcgggaaacctgtcgtg
ccagctgcattaatgaatcggccaacgcgcggggagaggcggtttgcgtattgggcgctcttccgcttcctcgctcactgactcgctgcgctcggtcgttcg
gctgcggcgagcggtatcagctcactcaaaggcggtaatacggttatccacagaatcaggggataacgcaggaaagaacatgtgagcaaaaggcc
agcaaaaggccaggaaccgtaaaaaggccgcgttgctggcgtttttccataggctccgcccccctgacgagcatcacaaaaatcgacgctcaagtca
gaggtggcgaaacccgacaggactataaagataccaggcgtttccccctggaagctccctcgtgcgctctcctgttccgaccctgccgcttaccggatac
ctgtccgcctttctcccttcgggaagcgtggcgctttctcatagctcacgctgtaggtatctcagttcggtgtaggtcgttcgctccaagctgggctgtgtgcac
gaaccccccgttcagcccgaccgctgcgccttatccggtaactatcgtcttgagtccaacccggtaagacacgacttatcgccactggcagcagccact
ggtaacaggattagcagagcgaggtatgtaggcggtgctacagagttcttgaagtggtggcctaactacggctacactagaagaacagtatttggtatct
gcgctctgctgaagccagttaccttcggaaaaagagttggtagctcttgatccggcaaacaaaccaccgctggtagcggtggtttttttgtttgcaagcagc
agattacgcgcagaaaaaaaggatctcaagaagatcctttgatcttttctacggggtctgacgctcagtggaacgaaaactcacgttaagggattttggtc
atgagattatcaaaaaggatcttcacctagatccttttaaattaaaaatgaagttttaaatcaatctaaagtatatatgagtaaacttggtctgacagttaccaa
tgcttaatcagtgaggcacctatctcagcgatctgtctatttcgttcatccatagttgcctgactccccgtcgtgtagataactacgatacgggagggcttacc
atctggccccagtgctgcaatgataccgcgagacccacgctcaccggctccagatttatcagcaataaaccagccagccggaagggccgagcgcag
aagtggtcctgcaactttatccgcctccatccagtctattaattgttgccgggaagctagagtaagtagttcgccagttaatagtttgcgcaacgttgttgccatt
gctacaggcatcgtggtgtcacgctcgtcgtttggtatggcttcattcagctccggttcccaacgatcaaggcgagttacatgatcccccatgttgtgcaaaa
aagcggttagctccttcggtcctccgatcgttgtcagaagtaagttggccgcagtgttatcactcatggttatggcagcactgcataattctcttactgtcatgc
catccgtaagatgcttttctgtgactggtgagtactcaaccaagtcattctgagaatagtgtatgcggcgaccgagttgctcttgcccggcgtcaatacggga
taataccgcgccacatagcagaactttaaaagtgctcatcattggaaaacgttcttcggggcgaaaactctcaaggatcttaccgctgttgagatccagttc
gatgtaacccactcgtgcacccaactgatcttcagcatcttttactttcaccagcgtttctgggtgagcaaaaacaggaaggcaaaatgccgcaaaaaag
ggaataagggcgacacggaaatgttgaatactcatactcttcctttttcaatattattgaagcatttatcagggttattgtctcatgagcggatacatatttgaat
gtatttagaaaaataaacaaataggggttccgcgcacatttccccgaaaagtgccacctaaattgtaagcgttaatattttgttaaaattcgcgttaaatttttg
ttaaatcagctcattttttaaccaataggccgaaatcggcaaaatcccttataaatcaaaagaatagaccgagatagggttgagtgttgttccagtttggaa
caagagtccactattaaagaacgtggactccaacgtcaaagggcgaaaaaccgtctatcagggcgatggcccactacgtgaaccatcaccctaatca
agttttttggggtcgaggtgccgtaaagcactaaatcggaaccctaaagggagcccccgatttagagcttgacggggaaagccggcgaacgtggcga
gaaaggaagggaagaaagcgaaaggagcgggcgctagggcgctggcaagtgtagcggtcacgctgcgcgtaaccaccacacccgccgcgctta
atgcgccgctacagggcgcgtcccattcgccattcaggctgcgcaactgttgggaagggcgatcggtgcgggcctcttcgctattacgccagctggcga
aaggggggatgtgctgcaaggcgattaagttgggtaacgccagggttttcccagtcacgacgttgtaaaacgacggccagtgagcgcgcgtaatacgac

tcactatagggcgaattggagctccaccgcggtggcggccgctctagagagcttggcccattgcatacgttgtatccatatcataatatgtacatttatattgg
ctcatgtccaacattaccgccatgttgacattgattattgactagttattaatagtaatcaattacggggtcattagttcatagcccatatatggagttccgcgtta
cataacttacggtaaatggcccgcctggctgaccgcccaacgacccccgcccattgacgtcaataatgacgtatgttcccatagtaacgccaataggga
ctttccattgacgtcaatgggtggagtatttacggtaaactgcccacttggcagtacatcaagtgtatcatatgccaagtacgccccctattgacgtcaatga
cggtaaatggcccgcctggcattatgcccagtacatgaccttatgggactttcctacttggcagtacatctacgtattagtcatcgctattaccatggtgatgc
ggttttggcagtacatcaatgggcgtggatagcggtttgactcacggggatttccaagtctccaccccattgacgtcaatgggagtttgttttggcaccaaaa
tcaacgggactttccaaaatgtcgtaacaactccgccccattgacgcaaatgggcggtaggcgtgtacggtgggaggtctatataagcagagctcgttta
gtgaaccgtcagatcgcctggagacgccatccacgctgttttgacctccatagaagacaccgggaccgatccagcctccggtcgaccgatcctgagaa
cttcagggtgagtttggggacccttgattgttctttctttttcgctattgtaaaattcatgttatatggagggggcaaagttttcagggtgttgtttagaatgggaag
atgtcccttgtatcaccatggaccctcatgataattttgtttctttcactttctactctgttgacaaccattgtctcctcttattttcttttcattttctgtaactttttcgttaaa
ctttagcttgcatttgtaacgaatttttaaattcacttttgtttatttgtcagattgtaagtactttctctaatcactttttttcaaggcaatcagggtatattatattgtactt
cagcacagttttagagaacaattgttataattaaatgataaggtagaatatttctgcatataaattctggctggcgtggaaatattcttattggtagaaacaact
acaccctggtcatcatcctgcctttctctttatggttacaatgatatacactgtttgagatgaggataaaatactctgagtccaaaccgggcccctctgctaacc
atgttcatgccttcttctctttcctacagctcctgggcaacgtgctggttgttgtgctgtctcatcattttggcaaagaattcctcgagggccggatccgaattcctg
tatttgtctgaaaac

Supplementary references

1. Chen, K., *et al.* Engineering self-deliverable ribonucleoproteins for genome editing in the brain. *Nat Commun* **15**, 1727 (2024).
2. Camperi, J., Moshref, M., Dai, L. & Lee, H.Y. Physicochemical and Functional Characterization of Differential CRISPR-Cas9 Ribonucleoprotein Complexes. *Anal Chem* **94**, 1432-1440 (2022).
3. Lapinaite, A., *et al.* DNA capture by a CRISPR-Cas9-guided adenine base editor. *Science* **369**, 566-571 (2020).
4. Foss, D.V., *et al.* Peptide-mediated delivery of CRISPR enzymes for the efficient editing of primary human lymphocytes. *Nat Biomed Eng* **7**, 647-660 (2023).
5. Zhang, Z., *et al.* Efficient engineering of human and mouse primary cells using peptide-assisted genome editing. *Nat Biotechnol* **42**, 305-315 (2024).
6. Israelachvili, J.N. & Mitchell, D.J. A model for the packing of lipids in bilayer membranes. *Biochim Biophys Acta* **389**, 13-19 (1975).
7. Regan, D., Williams, J., Borri, P. & Langbein, W. Lipid Bilayer Thickness Measured by Quantitative DIC Reveals Phase Transitions and Effects of Substrate Hydrophilicity. *Langmuir* **35**, 13805-13814 (2019).
8. Wei, T., Cheng, Q., Min, Y.L., Olson, E.N. & Siegwart, D.J. Systemic nanoparticle delivery of CRISPR-Cas9 ribonucleoproteins for effective tissue specific genome editing. *Nat Commun* **11**, 3232 (2020).
9. Onuma, H., Sato, Y. & Harashima, H. Lipid nanoparticle-based ribonucleoprotein delivery for in vivo genome editing. *J Control Release* **355**, 406-416 (2023).

Captions for the supplementary videos

Video	Caption
Supplementary Video 1	3D volume reconstruction demonstrating the distribution of eGFP-positive cells in the posterior eye segment of mT/mG mouse treated with Cre AAV, imaged by two-photon microscopy. Numbers next to axes represent micrometers; RPE is the top most layer.
Supplementary Video 2	3D volume reconstruction demonstrating the distribution of eGFP-positive cells in the posterior eye segment of mT/mG mouse treated with Cre-VSV VLP, imaged by two-photon microscopy. Numbers next to axes represent micrometers; RPE is the top most layer.
Supplementary Video 3	3D volume reconstruction demonstrating the distribution of eGFP-positive cells in the posterior eye segment of mT/mG mouse treated with free Cre recombinase, imaged by two-photon microscopy. Numbers next to axes represent micrometers; RPE is the top most layer.
Supplementary Video 4	3D volume reconstruction demonstrating the distribution of eGFP-positive cells in the posterior eye segment of mT/mG mouse treated with Cre recombinase complexed with Lipofectamine 3000, imaged by two-photon microscopy. Numbers next to axes represent micrometers; RPE is the top most layer.
Supplementary Video 5	3D volume reconstruction demonstrating the distribution of eGFP-positive cells in the posterior eye segment of mT/mG mouse treated with Cre recombinase with non-covalent 6xHis-CM18-PTD4 cell penetrating peptide, imaged by two-photon microscopy. Numbers next to axes represent micrometers; RPE is the top most layer.
Supplementary Video 6	3D volume reconstruction demonstrating the distribution of eGFP-positive cells in the posterior eye segment of mT/mG mouse treated with CPP5-Cre, imaged by two-photon microscopy. Numbers next to axes represent micrometers; RPE is the top most layer.
Supplementary Video 7	3D volume reconstruction demonstrating the distribution of eGFP-positive cells in the posterior eye segment of mT/mG mouse treated with TAT-Cre, imaged by two-photon microscopy. Numbers next to axes represent micrometers; RPE is the top most layer.
Supplementary Video 8	3D volume reconstruction demonstrating the distribution of eGFP-positive cells in the posterior eye segment of mT/mG mouse treated with ANTP-Cre, imaged by two-photon microscopy. Numbers next to axes represent micrometers; RPE is the top most layer.
Supplementary Video 9	Pupillary reflex of a wild-type C57BL/6 mouse.
Supplementary Video 10	Pupillary reflex of a *rd12* mouse.
Supplementary Video 11	Pupillary reflex of an ABE RNP LNP-treated *rd12* mouse.
