# Supplementary material for: Safer and efficient base editing and prime editing via ribonucleoproteins delivered through optimized lipid-nanoparticle formulations
Source: Nat Biomed Eng. 2024 Nov 28;9(1):57–78. doi: 10.1038/s41551-024-01296-2 (PMC11754100; doi:10.1038/s41551-024-01296-2)
Supplement: Supplementary file 2 — Reporting Summary [file 41551_2024_1296_MOESM2_ESM.pdf]

nature portfolio

Corresponding author(s): David Liu, Philip Felgner and Krzysztof Palczewski

Last updated by author(s): Oct 21, 2024

Reporting Summary

Nature Portfolio wishes to improve the reproducibility of the work that we publish. This form provides structure for consistency and transparency in reporting. For further information on Nature Portfolio policies, see our Editorial Policies and the Editorial Policy Checklist.

Statistics

For all statistical analyses, confirm that the following items are present in the figure legend, table legend, main text, or Methods section.

n/a	Confirmed	
☐	☒	The exact sample size (*n*) for each experimental group/condition, given as a discrete number and unit of measurement
☐	☒	A statement on whether measurements were taken from distinct samples or whether the same sample was measured repeatedly
☐	☒	The statistical test(s) used AND whether they are one- or two-sided<br>*Only common tests should be described solely by name; describe more complex techniques in the Methods section.*
☒	☐	A description of all covariates tested
☐	☒	A description of any assumptions or corrections, such as tests of normality and adjustment for multiple comparisons
☐	☒	A full description of the statistical parameters including central tendency (e.g. means) or other basic estimates (e.g. regression coefficient) AND variation (e.g. standard deviation) or associated estimates of uncertainty (e.g. confidence intervals)
☒	☐	For null hypothesis testing, the test statistic (e.g. *F*, *t*, *r*) with confidence intervals, effect sizes, degrees of freedom and *P* value noted<br>*Give P values as exact values whenever suitable.*
☒	☐	For Bayesian analysis, information on the choice of priors and Markov chain Monte Carlo settings
☒	☐	For hierarchical and complex designs, identification of the appropriate level for tests and full reporting of outcomes
☒	☐	Estimates of effect sizes (e.g. Cohen's *d*, Pearson's *r*), indicating how they were calculated

Our web collection on statistics for biologists contains articles on many of the points above.

Software and code

Policy information about availability of computer code

Data collection	MatLab 2021 (visual cortex recording and stimualtion); Malvern ZS Xplorer 3.30 (dynamic light scattering (DLS) and particle sizing); Espion 6.61.12 (ERG data acquisition and analysis); NovoExpress 1.6.2 (flow cytometry); Agilent Chemstation 11 (HPLC data acquisition); Illumina MiSeq Reporter 2.6 (generating fastq NGS files); BioLogic DuoFlow 5.30 (fast protein liquid chromatography); NanoDrop 1000 3.8.1 (DNA/RNA/protein quantification); SoftMax Pro 7.1 (ELISA and protein quantification); Bio-Rad CFX Maestro 4.1 (differential scanning fluorimetry); BioRad ImageLab Touch 2.4 (DNA/protein gel imaging); Keyence BZ-X800 Viewer 1.1.1.3 (fluorescence microscopy)
Data analysis	GraphPad Prism 10 (general data analysis and statistical testing); CRISPResso2 2.2.7 (NGS analysis); ImageLab 6.1.0 (DNA/protein gel and blot analysis); XnView 2.51.4 (gel photograph processing); Active Presenter 9 (pupillometry); Adobe Premiere Rush 2024 (pupillometry quantification); Espion 6.61.12 (ERG analysis); Microsoft Excel 365 (general data organization and analysis); BZ-X800 Analyzer 1.1.1.2 (fluorescence microscopy); ChemDraw 22.2.0.3300 (chemical-structure figures).

For manuscripts utilizing custom algorithms or software that are central to the research but not yet described in published literature, software must be made available to editors and reviewers. We strongly encourage code deposition in a community repository (e.g. GitHub). See the Nature Portfolio guidelines for submitting code & software for further information.

Data

Policy information about availability of data

All manuscripts must include a data availability statement. This statement should provide the following information, where applicable:

- Accession codes, unique identifiers, or web links for publicly available datasets
- A description of any restrictions on data availability
- For clinical datasets or third party data, please ensure that the statement adheres to our policy

High-throughput sequencing data are available from the National Center for Biotechnology Information Sequence Read Archive database, under accession PRJNA1124167. Source data for the figures are provided with this paper. The raw and analysed datasets generated during the study are available for research purposes from the corresponding authors on reasonable request.

Research involving human participants, their data, or biological material

Policy information about studies with human participants or human data. See also policy information about sex, gender (identity/presentation), and sexual orientation and race, ethnicity and racism.

Reporting on sex and gender	The study did not involve human research participants.
Reporting on race, ethnicity, or other socially relevant groupings	–
Population characteristics	–
Recruitment	–
Ethics oversight	–

Note that full information on the approval of the study protocol must also be provided in the manuscript.

Field-specific reporting

Please select the one below that is the best fit for your research. If you are not sure, read the appropriate sections before making your selection.

☒ Life sciences ☐ Behavioural & social sciences ☐ Ecological, evolutionary & environmental sciences

For a reference copy of the document with all sections, see nature.com/documents/nr-reporting-summary-flat.pdf

Life sciences study design

All studies must disclose on these points even when the disclosure is negative.

Sample size	No sample sizes were predetermined. Animals were treated by litter cohorts (5–9 animals per litter, 1–2 eyes per animal). Sample sizes were at least 5 animals per group. In vitro cell studies included at least 3 replicates per experiment.
Data exclusions	Mice with failed subretinal injections were excluded on the basis of no recordable ERG a-wave or b-wave by a blinded observer. This exclusion criteria was pre-established.
Replication	The in vitro experiments were replicated at least three times independently. All animal experiments were replicated at least five times independently.
Randomization	In all studies, samples and animals were assigned randomly to treatment groups.
Blinding	Deep sequencing, retinoid and ERG analysis were conducted blindly. The other experiments were not conducted blindly.

Reporting for specific materials, systems and methods

We require information from authors about some types of materials, experimental systems and methods used in many studies. Here, indicate whether each material, system or method listed is relevant to your study. If you are not sure if a list item applies to your research, read the appropriate section before selecting a response.

Materials & experimental systems

n/a	Involved in the study	
☐	☒	Antibodies
☐	☒	Eukaryotic cell lines
☒	☐	Palaeontology and archaeology
☐	☒	Animals and other organisms
☒	☐	Clinical data
☒	☐	Dual use research of concern
☒	☐	Plants

Methods

n/a	Involved in the study	
☒	☐	ChIP-seq
☐	☒	Flow cytometry
☒	☐	MRI-based neuroimaging

Antibodies

Antibodies used	Mouse 1D4 1:1000-1:10000 (in-house), mouse anti-RPE65 1:100-1:1000 (in-house), mouse anti-SpCas91:1000-1:5000 (clone 7A9, Biolegend #844302), rabbit-anti-beta-actin 1:2000 (polyclonal, Cell Signaling Technology #4970S), horse anti-mouse-IgG-HRP 1:2500-1:5000 (Vector Laboratories, #PI-2000-1), goat anti-rabbit-IgG 1:2500 (Cell Signaling Technology, 7074S), rabbit anti-ZO-1 1:100 (polyclonal, Thermo #617300), mouse anti-Cre recombinase 1:1000 (Biolegend #908002), Alexa Fluor 555 goat anti-mouse IgG 1:200 (Thermo #A11032), Alexa Fluor 647 goat anti-rabbit IgG 1:200 (Thermo #A21245)
Validation	Each commercial antibody has been validated for species, application and specificity, as indicated by the manufacturer's website and relevant citations listed by the manufacturer. In-house 1D4 and RPE65 antibodies have been validated in multiple publications from PI's and others' laboratories (PMID 24943310, 2006550, 2485225, 20100834, 33077938).

Eukaryotic cell lines

Policy information about cell lines and Sex and Gender in Research

Cell line source(s)	NIH/3T3 (ATCC), HEK293-loxP-GFP-RFP (Bsd) (GenTarget SC018-Bsd)
Authentication	The cell lines were authenticated by the supplier, with no further authentication performed.
Mycoplasma contamination	The cell lines were not tested for mycoplasma contamination.
Commonly misidentified lines (See ICLAC register)	No commonly misidentified cell lines were used.

Animals and other research organisms

Policy information about studies involving animals; ARRIVE guidelines recommended for reporting animal research, and Sex and Gender in Research

Laboratory animals	C547BL/6J, B6(A)-Rpe65 rd12/J (rd12), and B6.129(Cg)-Gt(ROSA)26Sortm4(ACTB-tdTomato,-EGFP)Luo/J (mTmG) mice were purchased from Jackson Laboratories (Bar Harbor, ME, USA), and mice were used between 4–12 weeks of age. Both male and female mice were used in equal numbers, with body weights between 15 and 25 grams, and were housed in the animal facility of the University of California, Irvine in a 12-hour-light (<10lux)/12-hour-dark cyclic environment. The temperature ranged from 75–76 °F, humidity from 30–40%, and the animals were given food and water ad libitum.
Wild animals	The study did not involve wild animals.
Reporting on sex	No sex-based stratification or sex-based analyses were performed. Both male and female mice were used.
Field-collected samples	The study did not involve samples collected from the field.
Ethics oversight	All animal procedures were approved by the IACUC of the University of California, Irvine, and conformed to the ARVO Statement for the Use of Animals in Ophthalmic and Vision Research and AAALAC guidelines.

Note that full information on the approval of the study protocol must also be provided in the manuscript.

Flow Cytometry

Plots

Confirm that:

☒ The axis labels state the marker and fluorochrome used (e.g. CD4-FITC).

☒ The axis scales are clearly visible. Include numbers along axes only for bottom left plot of group (a 'group' is an analysis of identical markers).

☒ All plots are contour plots with outliers or pseudocolor plots.

☒ A numerical value for number of cells or percentage (with statistics) is provided.

Methodology

Sample preparation	Cultured cell lines in tissue culture were detached by trypsinization and washed with PBS two times with 2% FBS. DAPI was added to the wash buffer for viability gating. No other staining was performed.
Instrument	Agilent NovoCyte Quanteon
Software	Agilent NovoExpress
Cell population abundance	No cell sorting was performed in this study.
Gating strategy	Cells were gated by size (FSC-A by SSC-A), singlets (FSC-A by FSC-H), viable cells by DAPI (Pacific Blue filter). GFP versus mCherry/tdTomato were acquired using FITC and PE filters, respectively. A gating strategy is shown in the Supplementary Information.

☒ Tick this box to confirm that a figure exemplifying the gating strategy is provided in the Supplementary Information.
